# Burden of Disease Caused by Otitis Media: Systematic Review and Global Estimates

**DOI:** 10.1371/journal.pone.0036226

**Published:** 2012-04-30

**Authors:** Lorenzo Monasta, Luca Ronfani, Federico Marchetti, Marcella Montico, Liza Vecchi Brumatti, Alessandro Bavcar, Domenico Grasso, Chiara Barbiero, Giorgio Tamburlini

**Affiliations:** 1 Epidemiology and Biostatistics Unit, Institute for Maternal and Child Health - IRCCS “Burlo Garofolo”, Trieste, Italy; 2 Department of Paediatrics, Institute for Maternal and Child Health - IRCCS “Burlo Garofolo”, Trieste, Italy; 3 Research Direction, Institute for Maternal and Child Health - IRCCS “Burlo Garofolo”, Trieste, Italy; 4 Unit for Health Services Research and International Health, Institute for Maternal and Child Health - IRCCS “Burlo Garofolo”, Trieste, Italy; 5 Ear, Nose and Throat Unit, Department of Paediatric Surgery, Institute for Maternal and Child Health - IRCCS “Burlo Garofolo”, Trieste, Italy; University of Massachusetts Medical School, United States of America

## Abstract

**Background:**

Otitis media (OM) is a leading cause of health care visits and drugs prescription. Its complications and sequelae are important causes of preventable hearing loss, particularly in developing countries. Within the Global Burden of Diseases, Injuries, and Risk Factors Study, for the year 2005 we estimated the incidence of acute OM, chronic suppurative OM, and related hearing loss and mortality for all ages and the 21 WHO regional areas.

**Methods:**

We identified risk factors, complications and sequelae of OM. We carried out an extensive literature review (Medline, Embase, Lilacs and Wholis) which lead to the selection of 114 papers comprising relevant data. Data were available from 15 of the 21 WHO regions. To estimate incidence and prevalence for all countries we adopted a two stage approach based on risk factors formulas and regression modelling.

**Results:**

Acute OM incidence rate is 10.85% i.e. 709million cases each year with 51% of these occurring in under-fives. Chronic suppurative OM incidence rate is 4.76‰ i.e. 31million cases, with 22.6% of cases occurring annually in under-fives. OM-related hearing impairment has a prevalence of 30.82 per ten-thousand. Each year 21thousand people die due to complications of OM.

**Conclusions:**

Our study is the first attempt to systematically review the available information and provide global estimates for OM and related conditions. The overall burden deriving from AOM, CSOM and their sequelae is considerable, particularly in the first five years of life and in the poorest countries. The findings call for incorporating OM-focused action within preventive and case management strategies, with emphasis on the more affected.

## Introduction

Acute otitis media (AOM) is a very common condition and a leading cause of health care visits and antibiotic prescription [1]. Studies carried out in developed countries show that by their third birthday 80% of children will have experienced at least one episode of AOM [2], [3] and 40% will have six or more recurrences by the age of seven years [4]. As with most infectious diseases, the burden of AOM varies substantially across countries, the main differences residing in the frequency of suppurative complications such as mastoiditis and meningitis and of sequelae such as hearing loss due to chronic suppurative otitis media (CSOM) [1], [3]. CSOM is an important cause of preventable hearing loss, particularly in the developing world [5], and a reason of serious concern, particularly in children, because it may have long-term effects on early communication, language development, auditory processing, psychosocial and cognitive development, and educational progress and achievement [6].

Based on prevalence surveys, which vary widely in disease definition, sampling methods, and methodological quality, WHO estimated that 28 thousand deaths every year are attributable to complications of OM [6]. OM and CSOM can lead to death mainly through meningitis and brain abscess. In addition WHO estimated that between 65 and 330 million individuals suffer from CSOM (show signs of CSOM), 50% of whom suffer from hearing impairment [6].

In industrialised countries, hearing loss (both conductive and sensorineural) is known to be the third most prevalent chronic condition in older adults after hypertension and arthropathy with considerable implications on physical and mental health [7], [8]. Information on the adult population of less industrialised countries is scanty [5]. A better knowledge of the incidence and prevalence of AOM and its complications across ages and geographical regions is necessary to adequately assess the need for interventions aimed at reducing its health, social and economic burden.

As members of the Expert Group on pneumonia, meningitis, sepsis, otitis media, pertussis and influenza of the Global Burden of Diseases, Injuries, and Risk Factors Study, we estimated the global burden of otitis media for all ages for all of the 21 WHO regional areas [9], for the year 2005, through a systematic, transparent and comprehensive literature review.

## Methods

All aspects of the methods are described in more detail in Text S1.

**Figure 1:**
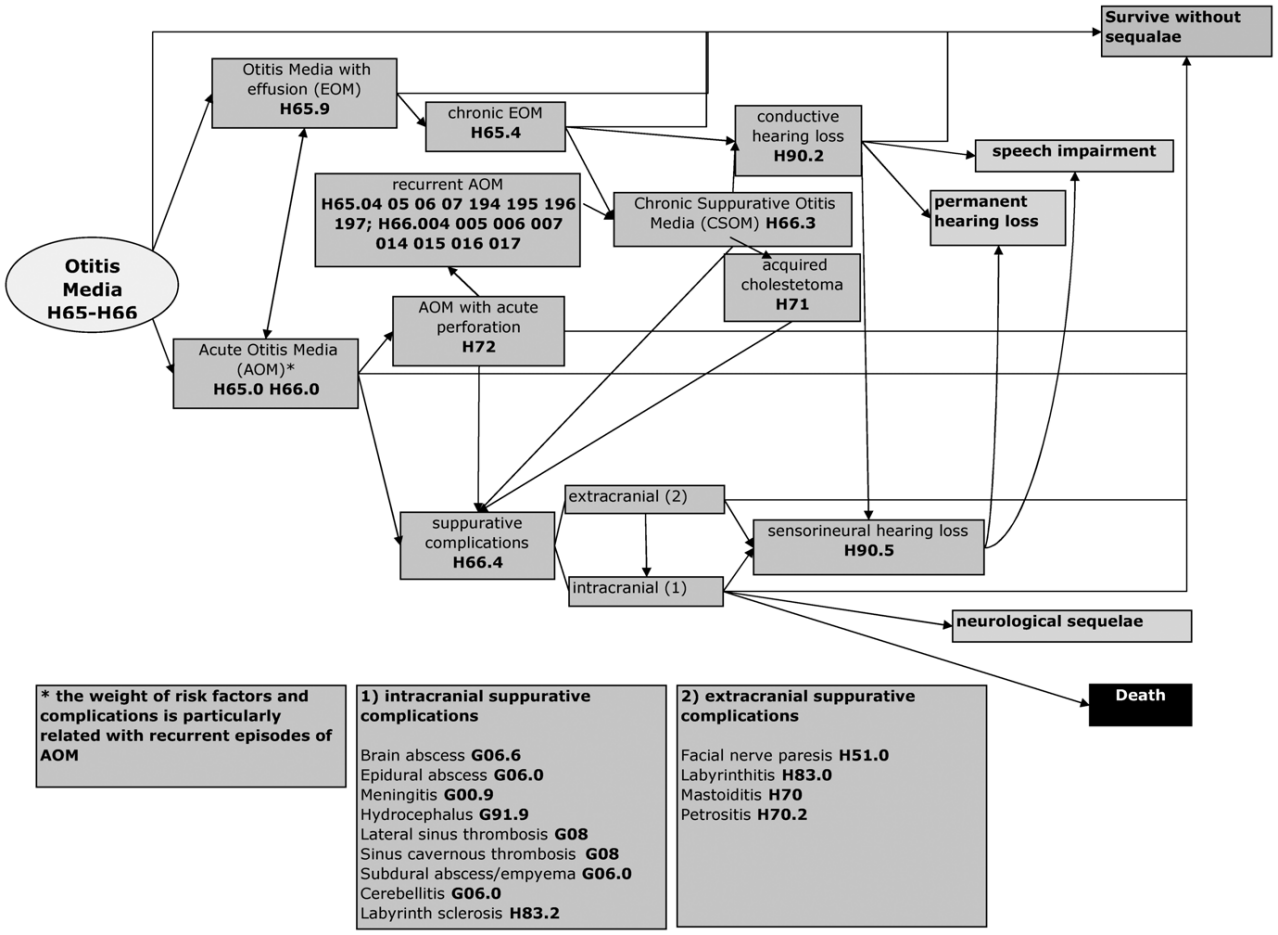
Sequelae of OM (complete scheme) with ICD-10-CM diagnosis codes.

Identification of risk factors, complications and sequelae of OM was the starting point (Figure 1, Table 1). Given the complexity of the causal pathways, the frequent overlapping of conditions, the lack of clear and consistent definitions and the fact that conditions included may have a mild and temporary nature or be rare, we focused in our review on AOM, CSOM, and on permanent Hearing Impairment (HI, both conductive and sensorineural) caused by OM (Figure S1). This simplification is consistent with that adopted in previous estimates of the OM GBD [10].

**Table 1:** Definitions of main conditions involved in the sequelae of otitis media.

**Otitis media** is the generic term for all types of inflammation of the middle ear [60], [61].
**Acute otitis media (AOM)** is usually a short-term inflammation of the middle ear, characterised by the rapid onset of one or more signs or symptoms of acute inflammation in the middle ear such as earache, tugging at the ear, fever, or irritability in the presence of a middle-ear effusion. It is often preceded by upper respiratory symptoms, including a cough and rhinorrhoea [39], [60], [61].
**Chronic suppurative otitis media (CSOM)** is defined as a persistent inflammatory process associated with a perforated tympanic membrane draining exudate for more than 6 weeks [62]. It is often associated with a cholesteatoma.
**Hearing impairment (HI):** is the total or partial inability to hear sound in one or both ears [63]. WHO defines disabling hearing impairment as having permanent unaided hearing threshold in the better ear of more than 30 dB in children aged up to 15 years, or more than 40 dB in adults at frequencies of 0.5, 1, 2, and 4 kHz [64].
**- Conductive hearing loss (CHL)** is the total or partial inability to hear sound in one or both ears because of some mechanical problem in the external or middle ear. The three tiny bones of the ear (ossicles) may fail to conduct sound to the cochlea, or the eardrum may fail to vibrate in response to sound. Fluid in the middle ear can cause CHL [63].
**- Sensorineural hearing loss (SNHL)** is the total or partial inability to hear sound in one or both ears resulting from a dysfunction of the inner ear. It most often occurs when the tiny hair cells (called cilia) that transmit sound through the ear are injured. This type of hearing loss is sometimes called “nerve damage,” although this is not accurate [63].

**Figure 2:**
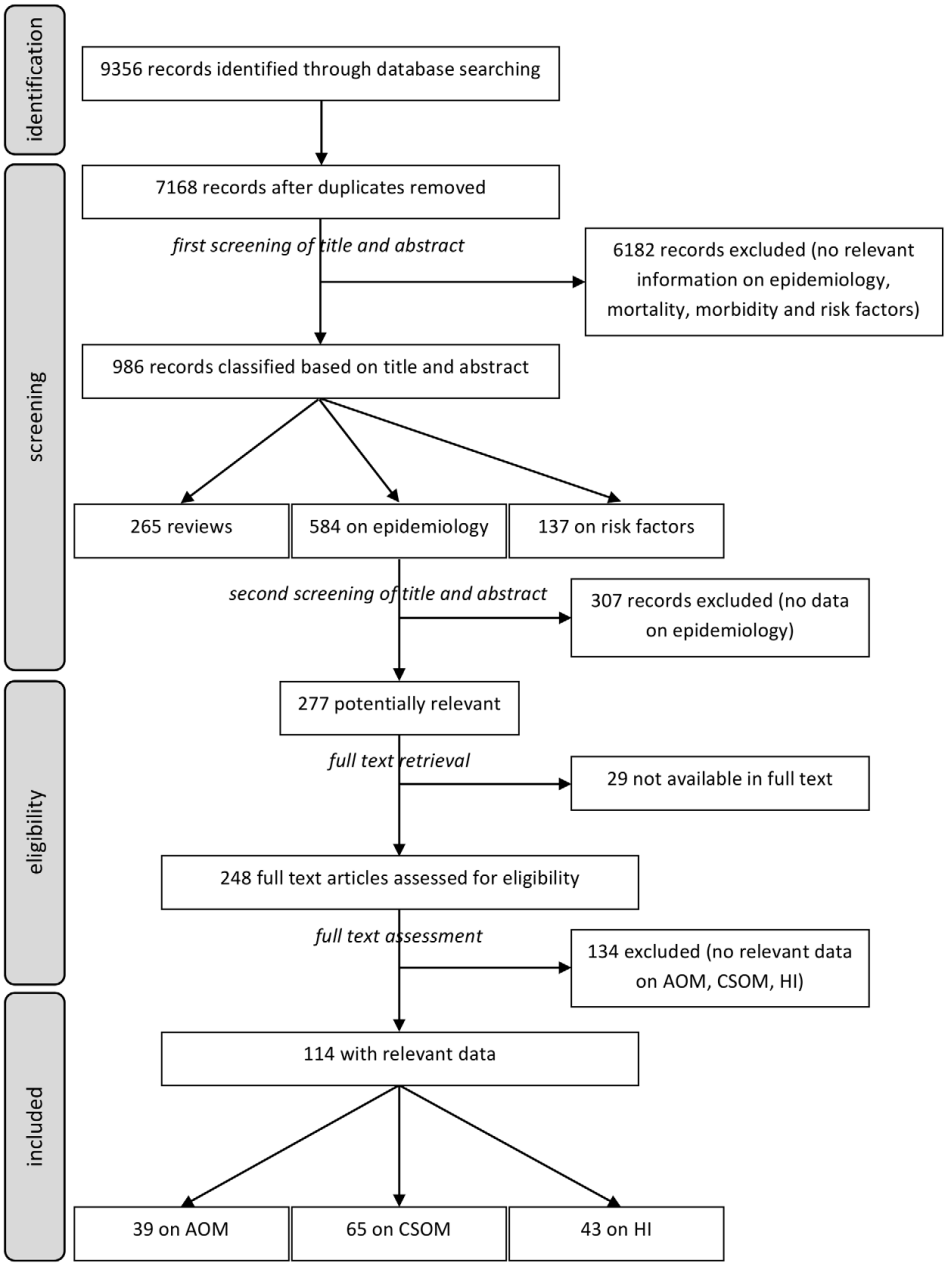
Screening process of articles on epidemiology of AOM, CSOM and HI, according to the PRISMA 2009 Flow Diagram. AOM: Acute Otitis Media; CSOM: Chronic Suppurative Otitis Media; HI: Hearing Impairment.

We searched for all articles presenting original data on incidence or prevalence of AOM or CSOM, and linking HI data with CSOM (proportion of HI caused by CSOM, or proportion of CSOM cases causing HI), published from 1980 to 2008. We did not formally define nor register a review protocol. The systematic review covering AOM, CSOM, HI and related risk factors was carried out on Medline (PubMed, last search 11/08/2008), Embase (last search 23/07/2008), Lilacs (last search 11/08/2008), Wholis (last search 11/08/2008), without any language restriction (details in Table S1). We retrieved 9356 records that became 7168 after duplication removal (Figure 2 for the complete flow diagram). We carried out a two-step evaluation to identify relevant papers:

1. by titles, abstract and keywords, aimed at identifying papers on epidemiology and risk factors of OM and reviews on OM (Figure S2). Papers on risk factors were used to review and complete the risk factors diagram (Figure S3).
2. by full text, when available, aimed at identifying papers on epidemiology of AOM, CSOM and HI. All articles with original data on AOM, CSOM or HI incidence or prevalence were included.

Information on mortality was collected through WHO vital registration databases, when available and reliable, or estimated for countries and areas where data were unavailable or unreliable.

Both the selection of relevant papers and data extraction were carried out independently by two of the authors (respectively by LM and AB or CB, and by LM and MM, LVB or CB). Any disagreement were resolved through discussion involving a third author (LR).

### Estimation process: incidence of AOM and CSOM

When only prevalence data were available, prevalence was transformed into incidence based on the average duration of AOM and CSOM. Although studies show that clinically symptomatic AOM usually has a shorter duration [11], [12] we assumed a duration of 21 days because when both prevalence and incidence data were available the actual duration, including the effusion was shown to be around 21 days [13]. This was the only real evidence on duration of AOM we could rely on. The average duration of CSOM in adults is 10 years [14]–[19].

The estimation process was divided into two phases. In the first and second phase all estimations were done by country within a specific area, the only exception being Oceania, for which we extrapolated country specific data to the whole region. As described below, while phase one estimations were carried out using a formula based on risk factors, the second phase was based on exponential regression models. Phase one, in fact, generated estimates for countries in regions in which we had original data. Phase two allowed us to expand the estimates to regions with no data and in general to fine tune all previously elaborated estimates.

Estimates were done by all age groups [9]. We did not calculate estimates by gender, since evidence on gender differences is scanty and conflicting.

#### AOM and CSOM estimates: first phase

The first phase was carried out by the following formula based on risk factors:

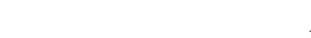

Were N_e/cy_ is the number of cases, Pop_new_ is the population, Inc_Ref_ is the incidence in the reference population, Prev_RF.new.i_ is the prevalence of a specific risk factor i in the new population, Prev_RF.Ref.i_ is the prevalence of the i risk factor in the reference population, RR_RF.i_ is the risk ratio for risk factor i.

Incidence in the reference population refers to the incidence for those countries for which data were available from the literature.

Risk estimates were extracted from the literature review [20]–[22] and in particular from day care center attendance (RR 2.45), exclusive bottle-feeding vs. exclusive breastfeeding up to six months (RR 2.00), parental smoking (RR 1.66), and malnutrition (RR 3.48).

The choice of limiting the list of risk factors to the ones above was determined, among the more relevant, by two fundamental reasons. The first being that we needed good quality data on risk ratios and, thus, we focused on data from meta-analyses. Malnutrition was an exception but we considered it to be a key factor in developing countries. The second reason is that we needed risk factors for which we could have reliable information for each country. Malnutrition was included only for developing countries (no data on malnutrition is available for developed countries and malnutrition is certainly more important in developing countries), while day-care centre attendance was only included for developed countries (no data on day-care centre is available for developing countries and is certainly more important in developed countries).

We used two different sets of risk factors. For Western Europe, North America High Income, Asia Pacific High Income and Australasia we used the following risk factors and associated Relative Risks (RRs): adults smoking (RR 1.66), exclusive breastfeeding at six months (RR 2.00), day-care attendance (RR 2.45). Data for these risk factors, for the year 2005, were taken from WHO, UNICEF and OSCE reports and databases [23]–[27] (www.oecd.org/els/social/family/database). For all other areas, day-care was substituted with under five children underweight (RR 3.48).

#### AOM and CSOM estimates: second phase

Areas for which we did not have any data were: Europe Central, Europe Eastern, Oceania, Asia East, Asia Central, Latin America Southern and Latin America Andean. To be able to cover these areas and to fine tune the estimates elaborated in phase one, we adopted a more robust strategy.

Phase two was based on exponential regression models. A model for AOM and a model for CSOM incidence rates were built for less industrialised areas. These models were based on the following factors: [23]–[25] prevalence of Acute Respiratory Infections (ARI) in children under five (ARI-U5), underweight prevalence in under five children (U-U5), under five mortality rate (MR-U5), proportion of children under six months exclusively breastfed (EBF), prevalence of adults smoking (AS). These models were based on data and phase one estimates from 45 countries and helped estimate AOM and CSOM incidence for the age group 1–4 years and validate the estimates of all countries. These models had an R^2^ of 0.83 for AOM and 0.85 for CSOM:

- AOM = 35.1859 * (1.0235^ARI-U5^) * (1.0060^U-U5^) * (0.9979^AS^) * (1.0079^MR-U5^) * (0.9846^EBF^)
- CSOM = 4.8451 * (1.0152^ARI-U5^) * (1.0298^U-U5^) * (1.0055^AS^) * (1.0021^MR-U5^) * (0.9872^EBF^)

We should not be surprised to see that single risk factors have coefficients that vary from >1 to <1 in different models (i.e. AS in the models above and EBF in the ones below), if we take into account the possibility of different interrelations between risk factors considered simultaneously.

For more industrialised areas (Europe Western, North America High Income and Australasia) we built exponential models to estimate AOM and CSOM for 1–4 years old children based on breastfeeding, adults smoking, day-care attendance (DCA) and under five mortality rate. The models were based on 22 countries Europe Western countries and helped re-estimate the other countries and validate the estimates of all countries. The R^2^ for these models were 0.61 for AOM and 0.79 for CSOM:

- AOM _1–4_ = 20.9264 * (0.9882^EBF^) * (1.0124^DCA^) * (1.0008^AS^) * (0.9998^MR-U5∧3^)
- CSOM _1–4_ = 0.8572 * (1.0010^EBF^) * (1.0110^DCA^) * (1.0123^AS^) * (1.0022^MR-U5∧3^)

The estimates of AOM and CSOM for Australia, Greenland and New Zealand were calculated as a weighted average of the estimates for aboriginals and non-aboriginals, with weight based on proportion of aboriginals to non-aboriginals by age group. For Asia Pacific High Income we kept the estimates based on the first phase model.

### Estimation process: prevalence of Hearing Impairment

According to the WHO definitions [28], [29] (Table S2), we estimated the prevalence of HI for all four degrees (slight, moderate, severe and profound). We used the following assumption to extend the estimates to the four degrees of HI or to estimate the WHO thresholds from other thresholds used (pg.6): “Where non-WHO thresholds used, the prevalence of hearing impairment at the WHO thresholds was interpolated assuming the log of the cumulative prevalence is linear with threshold. This relationship holds reasonably well in most studies [29].”

Again, a two phases estimation process was adopted in order to elaborate robust estimates. Details on the assumptions made and on both estimation phases are described in the Text S1.

#### HI estimates: first phase

We estimated cases of HI due to otitis media on the basis of the Oman age distribution of HI [30]. The Oman study was the only one reporting a complete age distribution of HI due to OM. The Oman distribution included HI caused by presbyacusis and accidents, thus we corrected this distribution to only include HI due to CSOM, and on the basis of the 0.19% of the population affected by HI due to CSOM calculated from Oman but adjusted to Europe, using Finland (0.001232%).

- Prevalence of HI>35 dB in best ear was extrapolated to countries on the basis of cases of CSOM per age group.
- Prevalence was cumulated to calculate cumulated cases and then cases by age group.
- Total for Western Europe were then calculated adding all cases per country and age group.

This was done for all regions for which we had countries with data: Brazil [19], China [31], India [32], [33], Malaysia [34], Nigeria [35], Oman [30] and South Africa [36].

#### HI estimates: second phase

Two regression models were used in the second phase: one model was used to estimate the prevalence of slight HI (25 dB to 40 dB), and another to estimate moderate HI (40 dB to 60 dB), using estimates from respectively 11 and 10 countries (China, India, Japan, Malaysia, Australia, Oman, Finland, Brazil, United States of America, South Africa, Nigeria) for which we had more reliable data and/or estimates. In the 40 dB model we used estimates from the same countries but excluding Malaysia, which did not have a good fit in the model compared with the other countries. The models, with regressors and coefficients, are reported in Text S1.

### Mortality estimates

We selected a number of countries for which we had reliable WHO 2005 Vital Registration data. Using these data and regressors such as incidence of AOM, CSOM, AOM in 1–4, CSOM in 1–4 and under five mortality rate, we developed and evaluated three different regression models. Models and reasons for choosing different models for different areas are described in detail in Text S1.

The 1^st^ model was based on overall otitis mortality rate ×10million from 12 countries and four regressors (U5 mortality, overall CSOMx1000, CSOM 1–4, AOM 1–4) had a R^2^ of 0.954 and a Standard Error (SE) of 8.09:

- MORT_1_ = −107.3699+0.0804*U5-MR+34.0711*CSOM−0.3516*CSOM1–4−0.4326*AOM1–4

The 2^nd^ was based on nine countries and two regressors (U5 Mortality rate and adult mortality): had a R^2^ of 0.996 and an SE of 2.60:

- MORT_2_ = 8.4023+0.5101*U5-MR−0.1222*Adult-MR+0.0168*(U5-MR)^2^

The 3^rd^ model was based on seven countries and the same four regressors as model one: had a R^2^ of 0.997 and an SE of 0.32:

- MORT_3_ = −7.8715+0.5709*U5-MR+2.3664*CSOM+0.0776*CSOM1–4−0.0382*AOM1–4

For all areas but five we selected the average of two models (1^st^ and 3^rd^). For Asia Pacific High Income we selected an average between the 2^nd^ and the 3^rd^ models. For Latin America Andean we used the 3^rd^ model. Australasia used the 2^nd^ model. The choice of the models to adopt for each area were made on the basis of models fitting vital registration data when available – even if less reliable than the ones used for the generation of the models – or on the basis of unreliable or negative estimates generated by the other models.

### Uncertainty bounds

For AOM and CSOM incidence and HI prevalence, uncertainty bounds were calculated using the standard error (SE) of the second phase regression models. Further sources of uncertainty for AOM and CSOM estimates derive from the data selected from original studies, from the adoption of estimates generated by the model used in phase one, from the decision on how to estimate the age distributions, and from the approach taken to convert prevalence to incidence. For HI estimates, uncertainty also derives from the use of previously estimated AOM and CSOM incidence, the adoption of WHO formulas to estimate different degrees of HI, the decisions on how to transpose original data on worst to best year, the use of data selected from original studies (including the heterogeneity in methods used to establish HI in different studies and the uncertainty of survey data) and finally the adoption of the Oman distribution in the estimation of HI by age. For mortality estimates, uncertainty bounds were calculated using the SE of the regression models. Further uncertainty derives from the methodology used to estimate the age distribution, and from the use of estimates of AOM and CSOM as regressors, which for their nature are subject to imprecision. To account for all sources of uncertainty, we increased the confidence level to 99%. It is, however, important to emphasise that the increase from 95% to 99% should be considered as a way to include other sources of uncertainty and that by no means these bounds should be considered to express 99% confidence.

## Results

Out of 584 papers on OM epidemiology, 307 were excluded because they were clearly not providing information relevant to our study. Out of the remaining 277, 29 were not available in full text. We then evaluated the 248 papers available in full text and excluded further 134 because not relevant for our study. Out of the remaining 114 papers, 39 contained data on AOM, 65 on CSOM and 43 on HI (Figure 2).

Data on AOM were available from six regions, the majority of the studies (32 of 44) being carried out in Europe Western and North America High Income (Table S3). Data on HI/CSOM were available for a greater number of regions. We were able to find data on CSOM from 15 regions, data on proportion of CSOM causing HI from 10 regions and data on proportion of HI caused by OM from 11 regions. Data covering all four classes of indicators were available only for three regions, whilst for six regions no data were found. Overall, our review was able to collect some data on AOM, CSOM or HI from 15 out of 21 regions, while only three areas had data on all three conditions: Europe Western, North Africa/Middle East and North America High Income.

**Figure 3:**
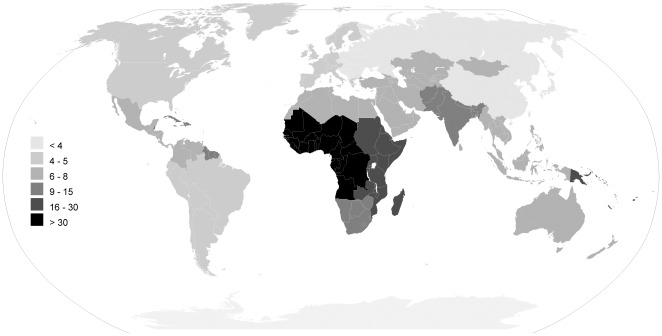
AOM incidence rate estimates for the year 2005 per hundred people, by the 21 WHO regions.

**Figure 4:**
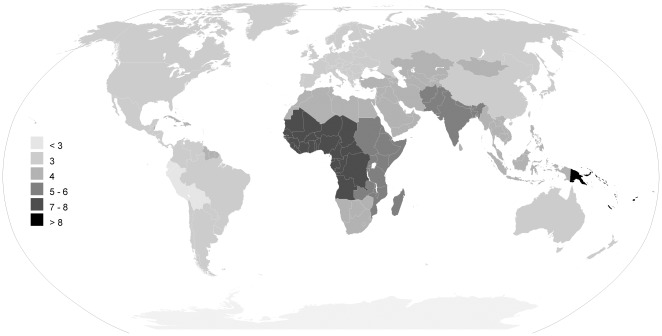
CSOM incidence rate estimates for the year 2005 per thousand people, by the 21 WHO regions.

**Figure 5:**
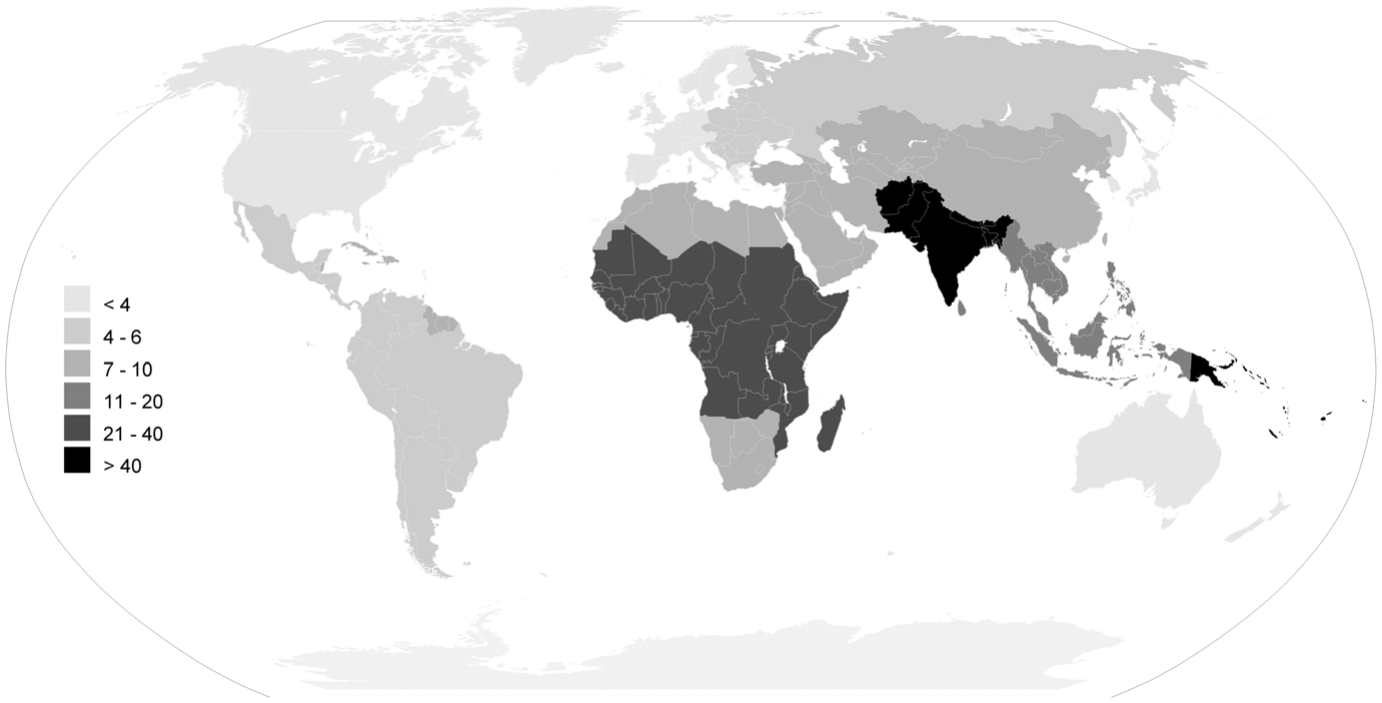
OM-associated HI (>25 dB for best ear) prevalence rate estimates for the year 2005 per ten thousand people, by the 21 WHO regions.

**Figure 6:**
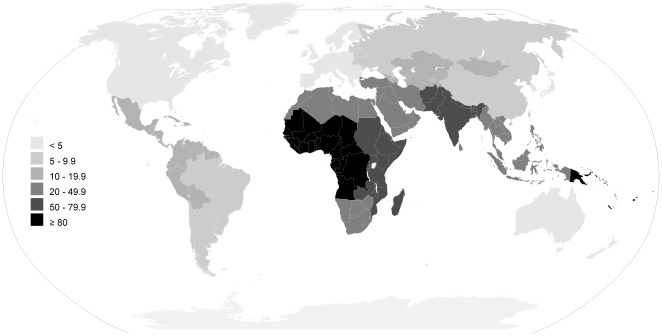
OM-associated mortality estimates for the year 2005 per ten million people, by the 21 WHO regions.

In Tables 2 and 3 (Table S4) and Figures 3, 4, 5, 6 we report global estimates for AOM, CSOM, HI and attributable mortality.

**Table 2:** AOM and CSOM incidence rate, HI prevalence and mortality estimates for the year 2005, by WHO areas.

Areas	AOM% incidence			CSOM‰ incidence			HI>25 dB best ear °/°°° prevalence			Deaths °/°°°°°°		
	AOM	MIN	MAX	CSOM	MIN	MAX	HI	MIN	MAX	Deaths	MIN	MAX
Asia Central	7.90	7.21	8.59	4.05	3.53	4.56	8.60	7.96	9.24	18.88	12.24	30.98
Asia East	3.93	3.24	4.61	3.67	3.16	4.18	9.69	9.05	10.32	9.32	9.04	9.62
Asia Pacific High Income	3.75	3.31	4.19	3.02	2.17	3.88	1.91	1.28	2.54	1.72	1.00	2.49
Asia South	14.52	13.84	15.21	6.56	6.05	7.08	97.04	96.37	97.71	68.88	68.21	69.02
Asia South East	8.15	7.47	8.84	4.69	4.17	5.20	14.67	14.03	15.31	27.16	25.18	29.81
Australasia	7.25	6.81	7.69	3.41	2.56	4.26	1.36	0.75	1.99	3.69	0.00	9.83
Caribbean	9.08	8.40	9.77	4.18	3.67	4.70	7.32	6.68	7.96	18.60	12.57	78.84
Europe Central	3.64	2.95	4.32	3.69	3.17	4.20	5.02	4.39	5.65	4.97	2.61	16.08
Europe Eastern	3.96	3.28	4.65	3.75	3.24	4.26	5.30	4.67	5.93	5.72	4.73	9.31
Europe Western	5.91	5.88	5.99	3.39	3.24	3.57	1.34	0.72	1.96	2.82	2.53	3.11
Latin America Andean	5.39	5.02	6.07	1.70	1.19	2.21	5.30	4.64	5.96	12.72	12.32	12.72
Latin America Central	6.78	6.10	7.47	3.92	3.40	4.43	5.28	4.65	5.91	12.36	9.16	16.96
Latin America Southern	4.25	3.56	4.93	3.60	3.09	4.12	5.15	4.52	5.79	6.00	3.94	13.53
Latin America Tropical	5.90	5.21	6.59	3.74	3.23	4.25	4.95	4.32	5.58	9.60	8.82	10.58
North Africa Middle East	8.67	7.98	9.35	4.41	3.90	4.92	8.63	7.99	9.27	21.96	18.48	27.17
North America High Income	5.46	5.40	5.53	3.06	2.82	3.30	1.41	0.79	2.04	1.63	1.23	1.90
Oceania	28.56	27.87	29.24	9.37	8.86	9.88	51.23	50.57	51.88	101.07	86.63	112.17
Sub-Saharan Africa Central	43.37	42.69	44.06	7.56	7.04	8.07	30.20	29.57	30.84	96.20	90.27	102.71
Sub-Saharan Africa East	22.81	22.13	23.50	6.06	5.55	6.57	31.91	31.26	32.56	73.49	68.30	78.20
Sub-Saharan Africa Southern	14.71	14.02	15.40	4.79	4.27	5.30	9.37	8.73	10.00	33.96	29.11	43.66
Sub-Saharan Africa West	43.36	42.68	44.05	7.22	6.71	7.74	34.37	33.74	35.00	91.82	85.26	98.34
Total	10.85	10.25	11.46	4.76	4.27	5.24	30.82	30.18	31.47	32.78	31.07	35.30

**Table 3:** Global AOM and CSOM incidence rate, HI prevalence and mortality estimates for the year 2005, by WHO age groups.

Age Groups	AOM% incidence			CSOM‰ incidence			HI>25 dB best ear °/°°° prevalence			Deaths °/°°°°°°		
	AOM	MIN	MAX	CSOM	MIN	MAX	HI	MIN	MAX	Deaths	MIN	MAX
0–11 m.	45.28	44.63	45.93	15.40	14.82	15.97	9.34	8.72	9.99	85.42	73.06	85.10
1–4	60.99	60.32	61.65	10.13	9.59	10.67	22.84	22.20	23.49	90.54	86.46	97.28
5–9	22.15	21.51	22.80	8.31	7.77	8.84	26.28	25.63	26.93	38.86	37.21	41.85
10–14	18.50	17.86	19.15	3.89	3.35	4.43	26.95	26.30	27.59	32.50	31.00	34.75
15–19	3.53	2.90	4.16	3.36	2.88	3.85	26.87	26.22	27.51	19.47	19.01	21.17
20–24	3.14	2.52	3.77	4.80	4.25	5.38	29.68	29.04	30.32	13.72	12.86	14.84
25–34	1.56	0.95	2.17	3.31	2.80	3.82	30.61	29.96	31.25	13.56	12.88	14.72
35–44	1.49	0.90	2.08	3.17	2.71	3.64	32.85	32.21	33.49	19.12	18.02	20.91
45–54	1.77	1.20	2.35	4.10	3.62	4.60	35.32	34.68	35.96	14.82	14.00	16.04
55–64	1.92	1.37	2.47	3.74	3.34	4.14	39.58	38.94	40.22	23.36	22.14	25.28
65–74	2.10	1.57	2.65	2.47	2.12	2.77	45.05	44.42	45.69	49.26	46.88	53.72
75–84	2.34	1.86	2.83	2.55	2.28	2.77	43.24	42.60	43.88	156.19	149.09	168.15
85+	2.27	1.86	2.68	2.73	2.56	2.83	37.73	37.10	38.37	179.01	167.26	200.77
Total	10.85	10.25	11.46	4.76	4.27	5.24	30.82	30.18	31.47	32.78	31.07	35.30

### AOM

Global AOM incidence rate (new episodes per hundred people per year) is, according to our estimates, 10.85% i.e. 709 million cases each year with 51% of these occurring in children under five years of age (U5) (Tables 2 and 3). Global incidence rate ranges from 3.64 for Europe Central (40% of cases occurring in children 0–5) to 43.36 and 43.37 for Sub-Saharan Africa West (56% in U5) and Central (58% in U5) respectively. Other areas with incidence lower than 5 are Asia Pacific High Income (3.75), Asia East (3.93), Europe Eastern (3.96) and Latin America Southern (4.25).

Global incidence rate is highest in the age group 1–4 (60.99%) and in the first year of life (45.28%). Incidence lowers to a minimum of 1.49 in the age group 35–44 and raises again to 2.3% after 75 years of age.

Areas with an incidence rate higher than 100% in the 1–4 age group are Oceania (114.98%), Sub-Saharan Africa Central (143.87%) and West (154.12%). An incidence rate of over 100% means we expect children in this age group to have an average of more than one episode of AOM in a one year time. In such age group four areas have an incidence below 30%: Latin America Andean (29.39%) and Southern (25.56%), Asia East (27.38%) and Asia Pacific High Income (24.21%).

### CSOM

Global estimated CSOM incidence rate is 4.76 per thousand people for a total of 31 million cases, with 22.6% of these cases occurring annually in U5 children. Latin America Andean is the area with the lowest incidence (1.70 per thousand), followed by Asia Pacific High Income (3.02) and North America High Income (3.06). Oceania has the highest incidence with 9.37, while two other areas have an incidence higher than 7: Sub-Saharan Africa Central (7.56) and West (7.22).

Globally, CSOM incidence rate is highest is the first year of life (15.40 per thousand) and reaches its lowest value after 65 years of age (2.51).

In the first year of life, Asia Pacific High Income has the lowest incidence rate (1.59 per thousand), while Oceania has the highest (35.96). The proportion of cases occurring in U5 vary from 1.8% in the Asia Pacific High Income, to 38.9% in Oceania and 41.0% in Sub-Saharan Africa Central.

### Hearing Impairment

HI caused by OM, defined as permanent hearing loss for best ear >25 dB, has a prevalence of 30.82 per ten thousand, with Europe Western, Australasia, North America High Income and Asia Pacific High Income all having a prevalence below 2 per ten thousand. Asia South has by far the highest prevalence (97.04), followed by Oceania (51.23) and then by Sub Sahara Africa West, East and Central all in the range of 30 to 35.

Prevalence increases with age, with 9.34 per ten thousand in the first year of life and a highest prevalence of 45.05 in the age group 65–74.

In Asia South, by the age of five, six out of one thousand children (6.02 per thousand) have a HI of at least 25 dB in the best ear caused by sequelae of OM. Oceania follows with 3.02 per thousand, then Sub-Saharan Africa West, East and Central with 2.21, 1.95 and 1.92 respectively. On the opposite extreme, less than one child per ten thousand suffers from HI by the age of five in Europe Western (0.55 per ten thousand), Australasia (0.62) North America High Income (0.64) and Asia Pacific High Income (0.80).

### Mortality

According to our estimates, globally, each year approximately 21 thousand people, i.e. 33 per 10 million people, die due to the complications of OM. Mortality is higher in the first year of life (85.4 per 10 million) and in the age group 1–4 years of age (90.5). it lowers to 13.6 in the 25–34 years age group and raises again to the highest values after 75 years of age (160.5).

North America High Income shows the lowest mortality (1.6 per 10 million), followed by Asia Pacific High Income (1.7), Europe Western (2.8) and Australasia (3.7). Oceania has the highest mortality with 101.1 deaths each year per 10 million, followed by Sub-Saharan Africa Central (96.2) and West (91.8). Other areas with a mortality higher than 50 per 10 million are Sub-Saharan Africa East (73.49) and Asia South (68.88).

## Discussion

Our study is the first attempt to systematically review the available information and provide global estimates for the incidence of AOM and CSOM, the prevalence of OM-related HI, and for OM-related mortality.

In our review we had to face a number of difficulties, the main ones being the scarcity or absence of data from some geographical areas and age groups and the heterogeneity of the available information, due to heterogeneity in study design, measurements and definitions. We provide estimates for 21 geographical sub-regions and 13 age groups. Since we were able to collect data on either AOM, CSOM or HI from 15 out of 21 regions, some of our estimates are based on modelling. In other cases we extrapolated to sub-regional level data that were collected in only a few countries. Data were particularly scarce on incidence/prevalence of AOM and CSOM in adult and elderly age groups.

While there are no other global estimates of AOM, our findings related to CSOM, HI and OM-related mortality can be compared with the estimates released by the WHO in 2004 [6]. It is noteworthy that our point estimate for CSOM prevalence (200.8 million cases) (Table S4) falls in between the range of estimates provided by WHO (65.5 to 328.2 million cases). Since we had no access to methods used by WHO to calculate previous estimates and we avoided any comparison or adjustment with WHO data while elaborating our methods and estimates, this may be considered as a reciprocal validation of both estimates. Our HI prevalence estimates, on the contrary, are much lower than those reported by WHO. Adding up the HI prevalent cases for all HI degrees (25 dB, 40 dB, 60 dB and 80 dB for best ear) we calculated a global estimate of 20.1 million cases. The WHO report provides an estimate, of 164.1 million people with CSOM-associated, but this was obtained by simply calculating 50% of CSOM prevalence. Our estimates of HI are based on a more complex and, in our opinion, more reliable model. In addition, our definition of HI includes only permanent HI while the WHO report does not explicitly exclude temporary HI. Finally, our mortality estimate (21.4 thousand deaths due to AOM, CSOM and related complications) is close to that reported by WHO (28 thousand) [6] which includes only mortality due to CSOM, but much higher than the one reported in the GBD 2001 and 2004 update (4 and 5 thousand respectively) [10], [37].

Not surprisingly, the incidence of AOM and, to a lesser extent, of CSOM is particularly high in the first five years – when its incidence is more than double that of pneumonia [38] – and in Sub-Saharan Africa and South Asia. The prevalence of HI follows a similar geographical distribution, but although it becomes significant quite early, it increases gradually to reach its maximum in the elderly population. Mortality attributable to OM is relatively low as compared to other causes. However, the overall burden deriving from the combination of AOM, CSOM and their sequelae is considerable, particularly in the first five years of life and in the poorest countries. AOM, CSOM and HI significantly contribute to temporary disability, suffering, use of health services and drug prescription and self-medication, particularly of non-steroidal anti-inflammatory drugs and antibiotics [39]. The direct and indirect costs of AOM, CSOM and related HI for health systems and households, the consequences on learning and working performances [40], [41] and the cost and adverse effects of drugs at both individual and population level represent an important burden for societies.

The findings of our review represent a serious challenge for health authorities. Early diagnosis and treatment of AOM, including the rational use of antibiotics should be improved, by incorporating clinical algorithms in current outpatient guidelines and by supporting the use of otoscopy in primary care practice. By doing this, prevention of complications and long term sequelae of AOM will be greatly strengthened. This is especially important for children under five, who bear 51% of all cases of AOM, and are particularly affected by the consequences on language development and school performance of early onset HI [42]–[44]. It is also particularly relevant for the poorest countries. We found that AOM incidence in Sub-Saharan Africa, South Asia and Oceania is two to eight times higher than in the remaining regions (Table 2, Figure 3). Here, co-morbidity with malnutrition, HIV and exposure to contaminated water greatly increases the risk of developing CSOM and its complications [45].

Health policies and programmes should incorporate preventive interventions as well as improved care seeking and access to effective treatment for common conditions such as AOM and CSOM.

Breastfeeding, smoking avoidance during and after pregnancy, and reduction of exposure to indoor air pollution are the pillars of prevention of AOM and its complications and sequelae [46]–[48] as well as of many other infant and child conditions. Vaccines against Streptococcus pneumoniae and HiB have been introduced to reduce the burden of child mortality consequent to meningitis and pneumonia. Although Hib vaccines (anti-capsular) do not target NTHi (non-capsular) which cause OM, a recent anti-Streptococcus pneumoniae capsular vaccine conjugated to a NTHi protein may be expected to reduce the incidence of AOM [49], and this should be taken into account when estimating and comparing the cost-effectiveness of vaccines [50] as well as of other preventive strategies.

Early diagnosis and treatment based on prompt care-seeking remain crucial particularly in the first years of life, also as a way to prevent HI. In developing countries, there have been attempts to standardize diagnosis and treatment of sick children through the IMCI strategy [51], [52]. Although IMCI does not focus explicitly on AOM, it ensures, when effectively implemented, effective treatment on a pragmatic basis to children with nonspecific signs and symptoms that may be caused by AOM, but it does not call for attention to the ear when examining the child to search for ear discharge. The family and community component of IMCI is supposed to improve care-seeking [52] for children with nonspecific signs and symptoms. Improved care-seeking needs to be combined with affordable access and effective care, which are still an utopia in most of the poorest countries and for the most disadvantaged groups [53]. There is the need to implement in clinical practice what we know about indications to antibiotic treatment of AOM in children, by adapting evidence from research studies [54]–[56] to local health system and risk factors. However, appropriately designed studies are necessary in order to assess to which extent strategies proven effective in developed countries (i.e. the wait and see strategy) [57], [58] are applicable in totally different contexts. Uncertainties still existing about treatment of CSOM in children should also be addressed [59].

In conclusion, our review calls for incorporating OM-focused action within preventive and case management strategies, with emphasis on high burden countries and for more research on epidemiology of OM and related conditions, focusing on high burden countries.

## Supporting Information

Text S1 **Expanded Methods Section.**
(PDF)

Figure S1 **Sequelae of OM, simplified scheme.**
(PDF)

Figure S2 **1^st^ screening by titles, abstract and keywords.**
(PDF)

Figure S3 **Risk factor diagram.**
(PDF)

Table S1 **Search strategies and results.**
(PDF)

Table S2 **WHO grades of hearing impairment.**
(PDF)

Table S3 **Data coverage.**
(PDF)

Table S4 **AOM and CSOM incidence and prevalence, HI cases and prevalence estimates, and Mortality estimates, by age groups and WHO region.**
(PDF)
