# Supplementary material for: Burden of Disease Caused by Otitis Media: Systematic Review and Global Estimates
Source: PLoS One. 2012 Apr 30;7(4):e36226. doi: 10.1371/journal.pone.0036226 (PMC3340347; doi:10.1371/journal.pone.0036226)
Supplement: Text S1 — Expanded Methods Section. (PDF) [file pone.0036226.s001.pdf]

Methods

Identification of risk factors, complications and sequelae of OM was the starting point.(Figure S1, Table S1). Given the complexity of the causal pathways, the frequent overlapping of conditions, the lack of clear and consistent definitions and the fact that conditions included may have a mild and temporary nature or be rare, we focused in our review on AOM, CSOM, and on permanent Hearing Impairment (HI, both conductive and sensorineural) caused by OM (Figure S2). This simplification is consistent with that adopted in previous estimates of the OM GBD.[1]

We searched for all articles presenting original data on incidence or prevalence of AOM or CSOM, and linking HI data with CSOM (proportion of HI caused by CSOM, or proportion of CSOM cases causing HI). The systematic review covering AOM, CSOM, HI and related risk factors was carried out on Medline (PubMed, last search 11/08/2008), Embase (last search 23/07/2008), Lilacs (last search 11/08/2008), Wholis (last search 11/08/2008), without any language restriction (detailed search strategies in Table S2). We retrieved 9356 records that became 7168 after duplication removal. We carried out a two-step evaluation to identify relevant papers:

1) **1st step**: by titles, abstract and keywords, aimed at identifying papers on epidemiology and risk factors of OM and reviews on OM (Figure S3). Papers on risk factors were used to review and complete the risk factors diagram (Figure S4).

2) **2nd step:** by full text, when available, aimed at identifying papers on epidemiology of AOM, CSOM and HI. All articles with original data on AOM, CSOM or HI incidence or prevalence were included.

Information on mortality was collected through WHO vital registration databases, when available and reliable, or estimated for countries and areas where data were unavailable or unreliable.

Relevant data were extracted and transcribed into records and divided by country and WHO region. Records included a reference code and information on the condition (AOM, CSOM or HI), the year the data were collected, whether they related to incidence or prevalence, the age of the subjects covered by the study, and whether data were stratified by urban/rural or by socio-economic status. Records also comprised all age ranges and data were inserted accordingly. The last columns of the database included information on HI when such data were available: proportion of cases of CSOM with HI, dB (decibel), and whether the data were for unilateral or bilateral HI.

Both the selection of relevant papers and data extraction were carried out independently by two of the authors (respectively by LM and AB, and by LM and MM or LVB). Any disagreement were resolved through discussion involving a third author (LR).

Estimation process: incidence of AOM and CSOM

When only prevalence data were available, prevalence was transformed into incidence based on the average duration of AOM and CSOM. Although studies show that clinically symptomatic AOM usually has a shorter duration [2,3] we assumed a duration of 21 days because when both prevalence and incidence data were available the actual duration, including the effusion was shown to be around 21 days.[4] This was the only real evidence on duration of AOM we could rely on. The average duration of CSOM in adults is 10 years.[5–10] For adolescents and young children, however, in the absence of data, and lacking in literature a method to convert prevalence to incidence when duration of illness is longer than age, we created an arbitrary duration scale for each age group: one year in the first year of life, two years duration in the 1-4 age group, 3 in the 5-9, 6 in the 10-14, 8 in the 15-19 and 9 in the 20-24.

The estimation process was divided into two phases. In the first and second phase all estimations were done by country within a specific area, the only exception being Oceania, for which we extrapolated country specific data to the whole region.

Estimates were generated for all age groups.[11] We did not calculate estimates by gender, since evidence on gender differences is scanty and conflicting.

Estimation of AOM and CSOM incidence: first phase

The first phase was carried out by the following formula based on risk factors:

$$\mathbf{N_{e/cy} = (Pop_{new}) \times (Inc_{Ref}) \times \{1 + \Sigma_{(i=1\rightarrow n)}[(Prev_{RF.new.i} - Prev_{RF.Ref.i}) \times (RR_{RF.i} - 1)]\}}$$

Were $\mathbf{N_{e/cy}}$ is the number of cases, $\mathbf{Pop_{new}}$ is the population we want to estimate the cases for, $\mathbf{Inc_{Ref}}$ is the incidence in the reference population, $\mathbf{Prev_{RF.new.i}}$ is the prevalence of a specific risk factor **i** in the new population, $\mathbf{Prev_{RF.Ref.i}}$ is the prevalence of the **i** risk factor in the reference population, $\mathbf{RR_{RF.i}}$ is the risk ratio for risk factor **i**.

Incidence in the reference population refers to the incidence for those countries for which data were available from the literature.

Risk estimates were extracted from the literature review and in particular from:

– Day-care centre attendance (RR)	2.45 (1.51-3.98)	Meta-analysis [12]
– Exclusive bottle-feeding vs. exclusive breastfeeding up to six months (RR)	2.00 (1.43-2.78)	Meta-analysis [13]
– Parental smoking (RR)	1.66 (1.33-2.06)	Meta-analysis [12]
– Malnutrition (RR)	3.48 (1.63-7.42)	Nigeria[14]

The choice of limiting the list of risk factors to the ones above was determined, among the more relevant, by two fundamental reasons. The first being that we needed good quality data on risk ratios and, thus, we focused on data from meta-analyses. The second reason is that we needed risk factors for which we could have reliable information for each country. Malnutrition was an exception but we considered it to be a key factor in developing countries. Malnutrition was included only for developing countries (no data on malnutrition is available for developed countries and malnutrition is certainly more important in developing countries), while day-care centre attendance was only included for developed countries (no data on day-care centre is available for developing countries and is certainly more important in developed countries).

We used two different sets of risk factors. For Western Europe, North America High Income, Asia Pacific High Income and Australasia we used the following risk factors and associated Relative Risks (RRs): adults smoking (RR 1.66), exclusive breastfeeding at six months (RR 2.00), day-care attendance (RR 2.45). Data for these risk factors, for the year 2005, were taken from WHO, UNICEF and OSCE reports and databases (www.oecd.org/els/social/family/database). [15–19] For all other areas, day-care was substituted with under five children underweight (RR 3.48).

For Western Europe, AOM incidence was estimated from data on incidence from Finland [20] and Spain [21] for ages up to 15 years. For older ages we used the Dutch College of General Practitioners (NHG) practice guideline from the Netherlands (note 2).[22] After plausibility checks, and extension of estimates to all age groups, “northern” countries were estimated with Finland while “southern” countries were estimated with Spain.

For CSOM we started with data from Israel,[23,24] Finland,[25] UK [26,27] and Greenland.[28–30]

Age distributions were not complete for the four countries, thus incidence estimates for the missing age groups were reciprocally estimated using available data. We then took the complete distribution of incidence for Finland and UK and build a combined average distribution. This combined distribution was used to estimate all other Western European Countries – with the exception of Israel and Greenland – with the RRs model. For the combined distribution we used average RRs from Finland and UK.

For North America High Income we extended the estimates we had for Western Europe after checking for coherence with data from literature which was, however, quite old for 2005 estimates.[4,31–34] For Asia Pacific High Income we used data from 1991 [35] and 1981 [36] with data on AOM and COM on all age groups from Rep of Korea and estimated 2005 with the risk formula and then all other countries in the area. Australasia was estimated with Western Europe with the risk formula but for Australia aboriginals were estimated separately. [37]

For the areas specified below, estimates were generated from original data from at least one country and then extended to the other countries with the risk factors formula. In case the age distribution of incidence of AOM and CSOM was not available, a hybrid distribution generated from data from Democratic Rep of Congo (90%),[38] South Africa (4%),[39,40] Korea (3%) [35,36] and Western Europe (3%). The age distribution is however irrelevant, considering that during phase two, two other age distributions were elaborated. It was relevant only when original data were not available for the 1-4 age group and incidence for this age group had to be estimated from other age groups.

For Asia South, CSOM estimates for years 1-4 were elaborated from data from Bangladesh[41,42] adjusted for both low and high Socio Economic Status with the differential expressed in data from India.[43] AOM for years 1-4 was also estimated with data from Bangladesh.[44] For North Africa / Middle East, CSOM and AOM 1-4 years, CSOM estimates were calculated from data on Saudi Arabia.[45] Data on AOM were also calculated from data from Saudi Arabia.[46,47] Both for CSOM and AOM the data were adjusted to the age group 1-4 with the help of the distribution on incidence from Australasia which was quite similar for the ages for which we had data. For Asia South East, CSOM were estimated from prevalence data Malaysia, Philippines, Thailand and Vietnam.[48–52] AOM estimates were

derived from Thailand prevalence data on ages 5-13 [49] shaped on ages 1-4 with the help of the distribution from Asia South and Western Europe. For Sub-Saharan Africa East CSOM was calculated from Tanzania 1994 on ages 5-19 [53] and shaped on age 1-4 with the hybrid distribution by age. For Sub-Saharan Africa Central, CSOM 1-4 was estimated with data from Democratic Republic of the Congo.[38] AOM 1-4 estimate was calculated on the ratio between AOM and CSOM cases from Zambia. For Sub-Saharan Africa West, CSOM were based on data from Nigeria [54,55]. AOM estimates were based on data from Nigeria [54]. For Sub-Saharan Africa Southern, estimates for CSOM were based on data from South Africa [40] and Swaziland [56] on children from 5-15 years of age, and then shaped on 1-4 years using the hybrid distribution by age. For the Caribbean, CSOM 1-4 was estimated from Jamaican data on OME prevalence in 5-7 years old,[57] translating OME to CSOM and AOM with data from Saudi Arabia [47]. These data were shaped to years 1-4 with the hybrid distribution by age. For Latin America Tropical estimate for CSOM in 1-4 was generated from data from Brazil on COM prevalence in 6 to 18 years old.[58] CSOM and AOM incidence for all age groups were then estimated by comparing CSOM incidence on 6-18 years old from Brazil with the same incidence in Dem Pop Rep of Korea chosen for their similarities. For Latin America Central, AOM incidence was estimated on the basis of Mexican data on ages 0-14.[59,60]

AOM and CSOM estimates: Second phase

Areas for which we did not have any data were: Europe Central, Europe Eastern, Oceania, Asia East, Asia Central, Latin America Southern and Latin America Andean. To be able to cover these areas and to fine tune the estimates elaborated in phase one, we adopted a more robust strategy.

Phase two was based on exponential regression models. A model for AOM and a model for CSOM incidence rates were built for less industrialised areas. These models were based on the following factors:[15–17] prevalence of Acute Respiratory Infections (ARI) in children under five (ARI-U5), underweight prevalence in under five children (U-U5), under five mortality rate (MR-U5), proportion of children under six months exclusively breastfed (EBF), prevalence of adults smoking (AS).

These models were based on data and phase one estimates from 45 countries (Sub-Saharan Africa East: Kenya, Uganda, Mozambique, Zambia, Ethiopia, Tanzania, Malawi. Sub-Saharan Africa Central: Democratic Republic of the Congo. Sub-Saharan Africa West: Nigeria, Cameroon, The Gambia, Mali, Senegal, Côte D'Ivoire, Sao Tome and Principe, Mauritania, Ghana, Chad, Burkina Faso. Sub-Saharan Africa Southern: Namibia, South Africa, Zimbabwe. Asia South East: Cambodia, Myanmar, Indonesia, Laos, Maldives, Philippines, and Vietnam. Caribbean: Dominican Republic and Jamaica. Latin America Tropical: Paraguay. Latin America Central: Guatemala) and helped estimate AOM and CSOM incidence for the age group 1-4 years and validate the estimates of all countries. These models had an R^2 of 0.83 for AOM and 0.85 for CSOM:

- $AOM = 35.1859 * (1.0235^{ARI\text{-}U5}) * (1.0060^{U\text{-}U5}) * (0.9979^{AS}) * (1.0079^{MR\text{-}U5}) * (0.9846^{EBF})$
- $CSOM = 4.8451 * (1.0152^{ARI\text{-}U5}) * (1.0298^{U\text{-}U5}) * (1.0055^{AS}) * (1.0021^{MR\text{-}U5}) * (0.9872^{EBF})$

We should not be surprised to see that single risk factors have coefficients that vary from >1 to <1 in different models (i.e. AS in the models above and EBF in the ones below), if we take into account the possibility of different interrelations between risk factors considered simultaneously.

For more industrialised areas (Europe Western, North America High Income and Australasia) we built exponential models to estimate AOM and CSOM for 1-4 years old children based on breastfeeding, adults smoking, day-care attendance (DCA) and under five mortality rate. The models were based on 22 countries Europe Western countries (Austria, Belgium, Cyprus, Denmark, Finland, France, Germany, Greece, Greenland, Iceland, Ireland, Israel, Italy, Luxembourg, Malta, The Netherlands, Norway, Portugal, Spain, Sweden, Switzerland, United Kingdom) and helped re-estimate the other countries and validate the estimates of all countries. The R^2 for these models were 0.61 for AOM and 0.79 for CSOM:

- $AOM_{1\text{-}4} = 20.9264 * (0.9882^{EBF}) * (1.0124^{DCA}) * (1.0008^{AS}) * (0.9998^{MR\text{-}U5\wedge 3})$
- $CSOM_{1\text{-}4} = 0.8572 * (1.0010^{EBF}) * (1.0110^{DCA}) * (1.0123^{AS}) * (1.0022^{MR\text{-}U5\wedge 3})$

The estimates of AOM and CSOM for Australia, Greenland and New Zealand were calculated as a weighted average of the estimates for aboriginals and non-aboriginals, with weight based on proportion of aboriginals to non-aboriginals by age group. For Asia Pacific High income we kept the estimates based on the first phase model.

To estimate the complete country distribution of incidence for all age groups, for AOM we selected two countries with extreme distributions (Chad, and an average between the Netherlands and Rep of Korea). The Netherlands and Republic of Korea had low incidence of AOM and robust age distribution data and estimates. The age distribution of AOM

incidence in Chad was particularly high and had been estimated in phase one with data from Nigeria on 0 to 14 years of age [54] and extended to older age groups following the proportion between AOM and CSOM incidence. For each age group, the two extreme incidence distributions were connected with linear regression, thus each intermediate age distribution of incidence could be estimated.

For CSOM we selected three countries with extreme distributions in terms of incidence rates (Democratic Rep of the Congo, Netherlands, Rep. of Korea) having the Democratic Rep. of the Congo high incidence and the Netherlands and Rep. of Korea low incidence. In this case, however, the Netherlands and the Rep. of Korea were kept separated because we noticed a different pattern in the age distribution of CSOM that could be explained as a further evolution in the reduction of incidence from the Netherlands to the Rep. of Korea Again, for the Netherlands and the Rep. of Korea we had solid age distribution estimates, while from the Democratic Republic of the Congo, we had original data on all age groups.[38] The three distributions were linked with linear regression two by two, i.e. Dem. Rep. of the Congo with the Netherlands and the Netherlands with Rep. of Korea.

Uncertainty bounds for AOM and CSOM estimates

Uncertainty bounds were calculated using the standard error (SE) of the second phase regression models. For Asia Pacific High Income, which were estimated by model from phase one, we assumed the SE to be the same as in the second phase regression models. Further sources of uncertainty derive from the data selected from original studies, from the estimates generated by the adoption of the model used in phase one, from the decision on how to estimate the age distributions, from the approach taken to convert prevalence to incidence. To account for all sources of uncertainty, which would be difficult to include in the model, we increased the confidence level to 99%. Thus, even if we kept the SE from the model developed in phase two, we calculated the uncertainty bounds for a confidence level of 99%. It is, however, important to emphasise that the increase from 95% to 99% should be considered as a way to include other sources of uncertainty and that by no means these bounds should be considered to express 99% confidence.

Estimation process: prevalence of Hearing Impairment

According to the WHO definitions [61,62] (Table S3), we estimated the prevalence of HI for all four degrees.

We used the following assumption to extend the estimates to the four degrees of HI or to estimate the WHO thresholds from other thresholds used (pg.6): “Where non-WHO thresholds used, the prevalence of hearing impairment at the WHO thresholds was interpolated assuming the log of the cumulative prevalence is linear with threshold. This relationship holds reasonably well in most studies.” [62]

If we only had data on HI in single ears, we considered 25% of HI in single ears to be HI in best ear: we calculated this proportion as an average of what we found in the data extracted from the literature.[47,63–65]
For Western Europe:

- In Finland 6.4% of CSOM has HI >35db in single ear;[66]
- Incidence of CSOM per 100 children per year = 0.077;
- Estimated 1.6% of CSOM has HI>35db in best ear (25% of 6.4%);
- This means 1.6% of 0.077% with CSOM = 0.001232%.

We estimated cases of HI due to otitis media on the basis of the Oman [67] age distribution of HI. The Oman study was the only one reporting a complete age distribution of HI due to OM. The Oman distribution included HI caused by presbyacusis and accidents, thus we corrected this distribution to only include HI due to CSOM, and on the basis of the 0.19% of the population affected by HI due to CSOM calculated from Oman but adjusted to Europe, using Finland (0.001232%).

- Prevalence of HI>35dB in best ear was extrapolated to countries on the basis of cases of CSOM per age group.
- Prevalence was cumulated to calculate cumulated cases and then cases by age group.
- Total for Western Europe were then calculated adding all cases per country and age group.

This was done for all regions for which we had countries with data: Brazil,[10] China,[68] India,[69,70] Malaysia,[50] Nigeria,[55] Oman [67] and South Africa.[40]

Once again, regression models were used in the second phase: one model was used to estimate the prevalence of slight HI (25dB to 40dB), and another to estimate moderate HI (40dB to 60dB), using estimates from respectively 11 and 10 countries (China, India, Japan, Malaysia, Australia, Oman, Finland, Brazil, United States of America, South Africa, Nigeria) for which we had more reliable data and/or estimates. In the 40dB model we used estimates from the same countries but excluding Malaysia, which did not have a good fit in the model compared with the other countries. The

two were exponential regression models using transformations of total incidence of AOM (ln(AOM)) and CSOM (ln(CSOM) and √(CSOM)), CSOM incidence in age group 1-4 ($(CSOM1\text{-}4)^3$), under five mortality rate (ln(U5-MR)) and mortality due to otitis media (√(Mort)) as regressors:

- $HI\text{-}25dB = 1.5130 * (0.2382^{\ln(AOM)}) * (0.7873^{\sqrt{CSOM}}) * (3.6676^{\sqrt{MORT}}) * (1.4317^{\ln(U5\text{-}MR)}) * (1.0002^{(CSOM1\text{-}4)^{\wedge}3}) * (3.1712^{\ln(CSOM)})$
- $HI\text{-}40dB = 440.51 * (1.1202^{\ln(AOM)}) * (0.0078^{\sqrt{CSOM}}) * (14.2427^{\sqrt{MORT}}) * (1.4525^{\ln(U5\text{-}MR)}) * (1.0002^{(CSOM1\text{-}4)^{\wedge}3}) * (66.1908^{\ln(CSOM)})$

Both models had a R^2 of 0.99. The other WHO thresholds were estimated using WHO assumptions on the relation between different degrees of HI.[62]

Unfortunately, we could only count on one complete age distribution of prevalence of HI [67] and we used this distribution to estimate the specific HI for all age groups from the general HI estimates with the regression models.

Uncertainty bounds for HI

Uncertainty bounds were calculated using the SE (SE=0.216823 for the 25dB model and SE=0.238823 for the 40dB model) of the second phase regression models for all areas. Other sources of uncertainty were the use of previously estimated AOM and CSOM incidence, the adoption formulas to transpose estimations (the WHO model to transpose 25, 40, 60 and 80dB) and decisions on how to transpose original data on worst to best year, the data selected from original studies (including the heterogeneity in methods used to establish HI in different studies and the uncertainty of survey data) and finally the adoption of the Oman distribution by age. Considering these other sources of uncertainty, as previously done with AOM and CSOM uncertainty bounds, we decided to increase the confidence level to 99%. Thus, even if we kept the SE from the estimation model, we calculated the uncertainty bounds for a confidence level of 99%. It is, however, important to emphasise that the increase from 95% to 99% should be considered as a way to include other sources of uncertainty and that by no means these bounds should be considered to express 99% confidence.

Mortality estimates

We selected a number of countries for which we had reliable WHO 2005 Vital Registration data (Argentina, Australia, Brazil, Colombia, Cuba, France, Hungary, Japan, Mexico, Netherlands, Rep. of Korea, Rep. of Moldova, South Africa, United Kingdom, United States of America, Venezuela), plus mortality estimates for China and India for the year 1990.[52] Using these data and regressors such as incidence of AOM, CSOM, AOM in 1-4, CSOM in 1-4 and under five mortality rate, we developed and evaluated three different regression models.

The 1st model was based on overall otitis mortality rate x 10million from 12 countries (Argentina, Brazil, China, Colombia, Cuba, Hungary, India, Mexico, Rep. of Moldova, South Africa, Venezuela) and four regressors (U5 mortality, overall CSOMx1000, CSOM 1-4, AOM 1-4) had a R^2 of 0.954 and a Standard Error (SE) of 8.09:

- $MORT_1 = –107.3699 + 0.0804*U5\text{-}MR + 34.0711*CSOM – 0.3516*CSOM1\text{-}4 – 0.4326*AOM1\text{-}4$

The 2nd was based on nine countries (Japan, Netherlands, USA, Venezuela, Mexico, Brazil, India, China and Western Europe) and two regressors (U5 Mortality rate and adult mortality): had a R^2 of 0.996 and an SE of 2.60:

- $MORT_2 = 8.4023 + 0.5101*U5\text{-}MR – 0.1222*Adult\text{-}MR + 0.0168*(U5\text{-}MR)^2$

The 3rd model was based on seven countries (Japan, Rep. of Korea, USA, Australia, Western Europe, Hungary and Rep. of Moldova) and the same four regressors as model one: had a R^2 of 0.997 and an SE of 0.32:

- $MORT_3 = –7.8715 + 0.5709*U5\text{-}MR + 2.3664*CSOM + 0.0776*CSOM1\text{-}4 – 0.0382*AOM1\text{-}4$

For all areas but five we selected the average of two models (1st and 3rd). For Asia Pacific High Income we selected an average between the 2nd and the 3rd models. For Latin America Andean we used the 3rd model. Australasia used the 2nd model. The choice of the models to adopt for each area were made on the basis of models fitting vital registration data when available – even if less reliable than the ones used for the generation of the models – or on the basis of unreliable or negative estimates generated by the other models.

These estimates were for the overall mortality. We then had to divide this mortality in all of the age groups. For Western Europe the age distribution of deaths was estimated on the basis of the proportion between deaths and AOM+CSOM cases in the following countries: France, the Netherlands and the U.K. For Latin America Central, the

deaths reported for Mexico were used to generate the distribution by age for the region. For Latin America Tropical, the same was done with Brazil. For North America High Income the distribution by age was shaped on an average of actual death registered between 1990 and 2005. For Sub-Saharan Africa Southern the age distribution was shaped on the actual deaths reported for South Africa for 2005.

For all other regions where data were unavailable or unreliable we followed a different approach. The age distribution was shaped according to the proportion between mortality and AOM+CSOM cases in Western Europe for the following regions: Europe Central, Europe Eastern, Asia Pacific High Income and Australasia. It was done following the proportion between mortality and AOM+CSOM cases in Latin America Central for the following regions: Caribbean, Latin America Southern, Asia East, Asia Central, Oceania, Asia South East, Asia South, Latin America Andean, North Africa Middle East, Sub-Saharan Africa Central, East and West. The choice of the areas to be estimated by using as reference Western Europe or Latin America Central was made on the basis of similarities in AOM and CSOM incidence distribution.

Uncertainty bounds for mortality estimates

Uncertainty bounds were calculated using the SE of the regression models. Further uncertainty derives from the methodology used to estimate the age distribution, and from the fact we used as regressors the estimates of AOM and CSOM, which for their nature are subject to imprecision due to estimation models and arbitrary decisions on how to perform them. Considering these other sources of uncertainty, as previously done with AOM, CSOM and HI uncertainty bounds, we decided to increase the confidence level and we calculated the uncertainty bounds for a confidence level of 99%. As previously mentioned, however, by no means these bounds should be considered to express 99% confidence. For North America High Income, the lowest limit was considered the actual data reported and the highest was calculated proportionally.

Figure S1. Sequelae of OM (complete scheme) with ICD-10-CM diagnosis codes

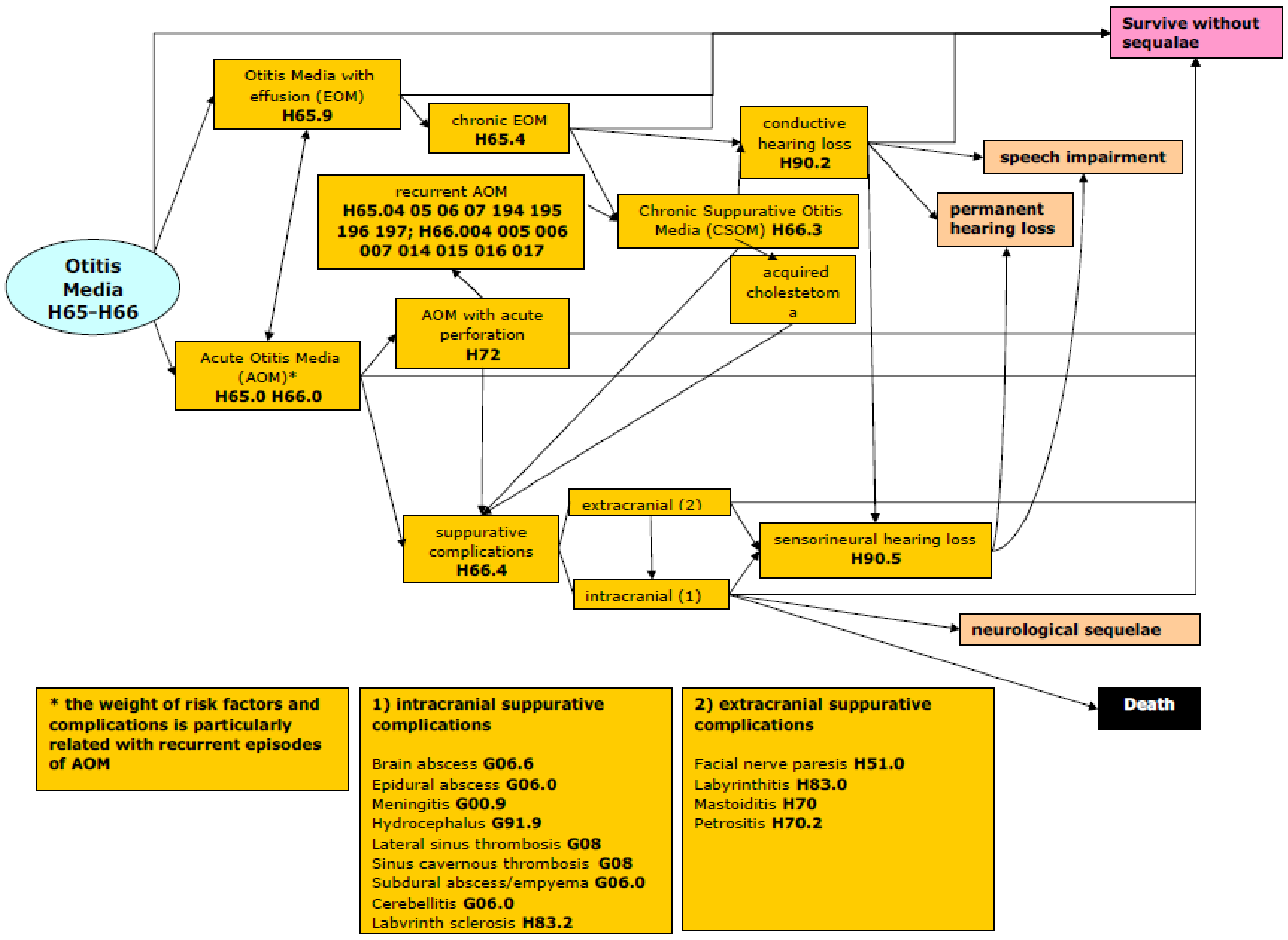

Figure S2. Sequelae of OM, simplified scheme

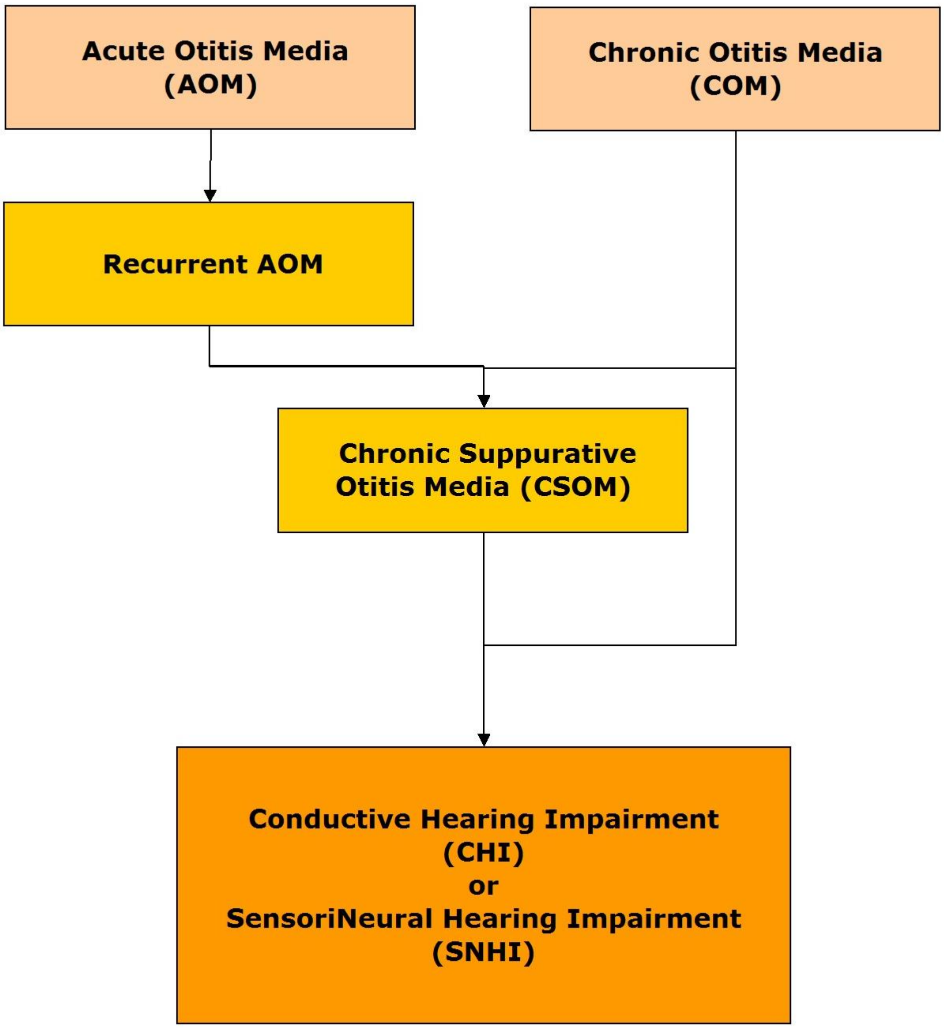

Figure S3. 1st screening by titles, abstract and keywords

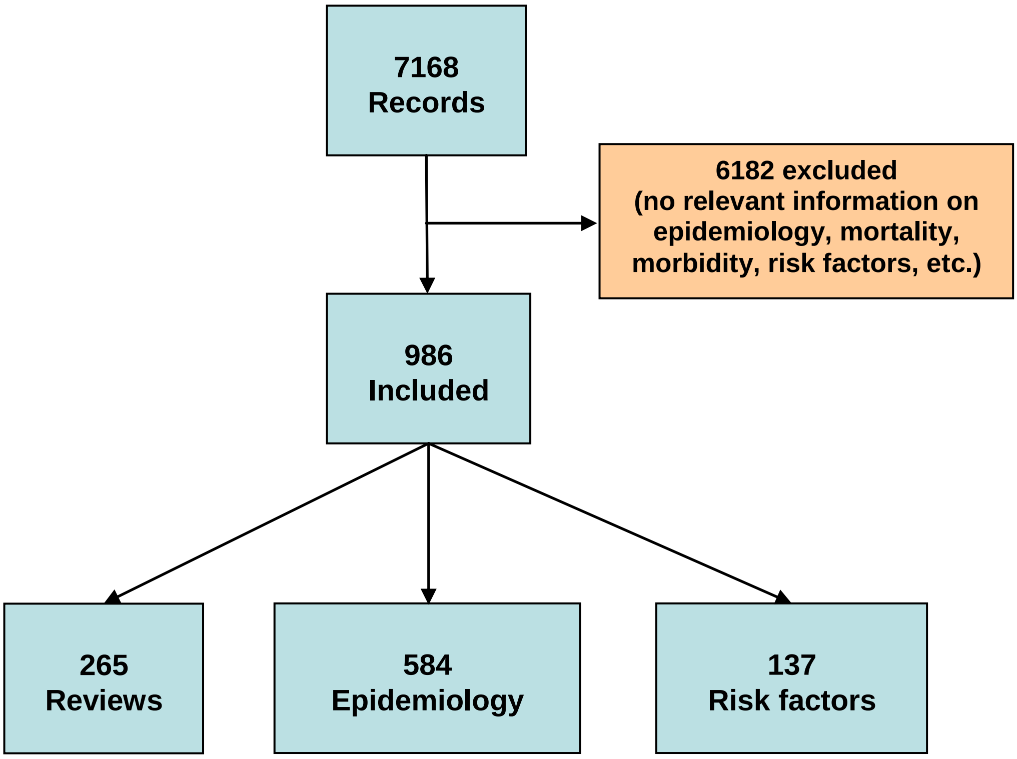

Figure S4. Risk factors diagram

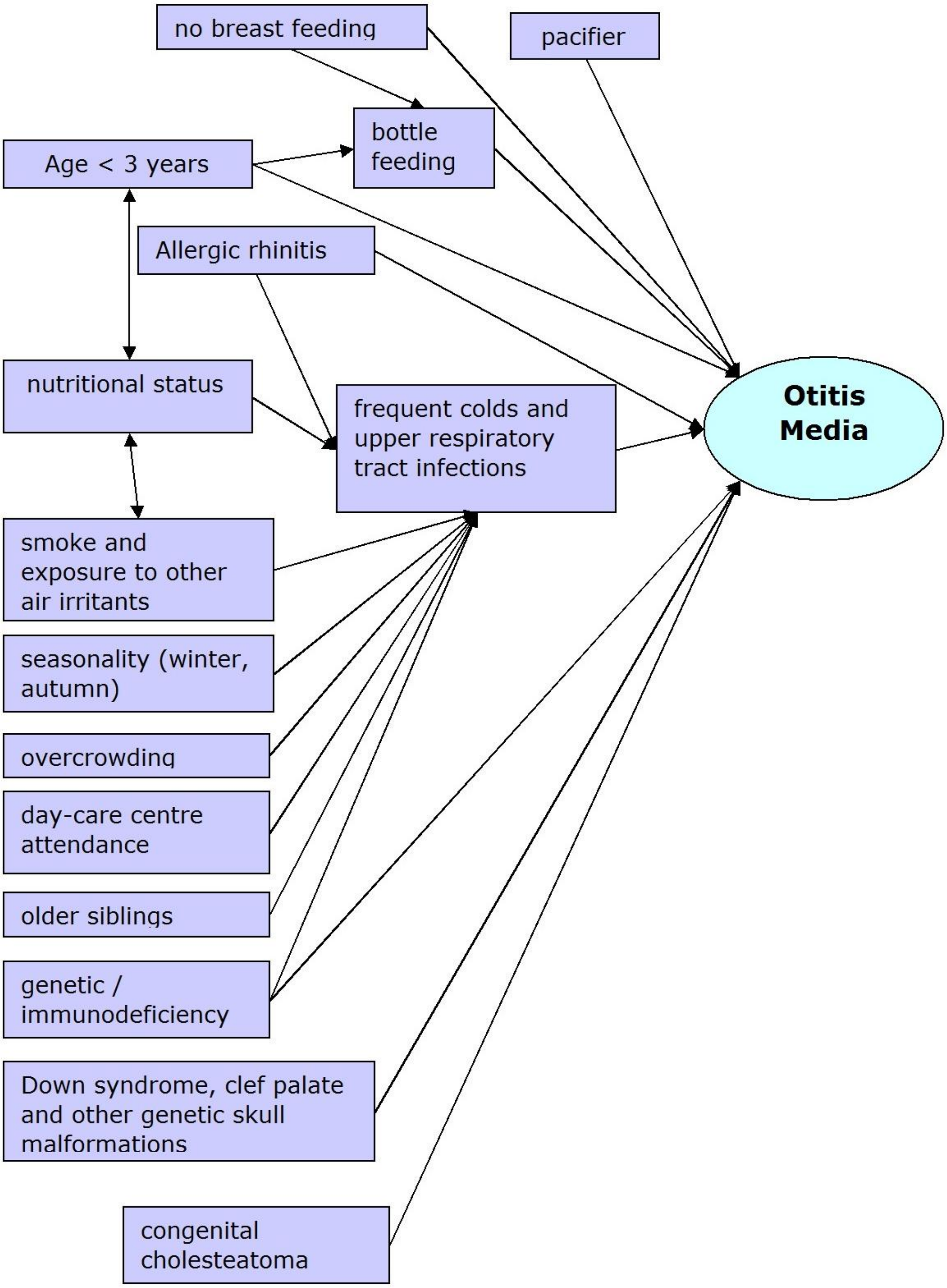

Table S1. Definitions of main conditions involved in the sequelae of otitis media

- **Otitis media** is the generic term for all types of inflammation of the middle ear [71,72].
- **Acute otitis media (AOM)** is usually a short-term inflammation of the middle ear, characterised by the rapid onset of one or more signs or symptoms of acute inflammation in the middle ear such as earache, tugging at the ear, fever, or irritability in the presence of a middle-ear effusion. It is often preceded by upper respiratory symptoms, including a cough and rhinorrhoea [71–73].
- **Otitis media with effusion (OME),** also called glue ear, can be defined as a middle-ear effusion without signs of acute inflammation or infection. It is often asymptomatic, and in particular earache is relatively uncommon. OME can arise *de novo* or following AOM [71–73].
- **Chronic OME (COME)** is diagnosed when middle-ear effusion (MEE) fails to resolve within 3 months. The chance of spontaneous resolution decreases the longer the effusion persists [74].
- **Recurrent AOM** refers to frequent episodes of the illness within a period of 6 or 12 months. The National Institute for Health and Clinical Excellence (NICE) has previously defined this as more than four episodes in 6 months [75]. An American guideline defines it as three or more episodes in 6 months, or four or more episodes in a year [76].
- **Chronic suppurative otitis media (CSOM)** is defined as a persistent inflammatory process associated with a perforated tympanic membrane draining exudate for more than 6 weeks [76]. It is often associated with a cholesteatoma.
- **Hearing impairment (HI):** is the total or partial inability to hear sound in one or both ears [77]. WHO defines disabling hearing impairment as having permanent unaided hearing threshold in the better ear of more than 30 dB in children aged up to 15 years, or more than 40 dB in adults at frequencies of 0.5, 1, 2, and 4 kHz [78].
    - **Conductive hearing loss** (CHL) is the total or partial inability to hear sound in one or both ears because of some mechanical problem in the external or middle ear. The three tiny bones of the ear (ossicles) may fail to conduct sound to the cochlea, or the eardrum may fail to vibrate in response to sound. Fluid in the middle ear can cause CHL [77].
    - **Sensorineural hearing loss** (SNHL) is the total or partial inability to hear sound in one or both ears resulting from a dysfunction of the inner ear. It most often occurs when the tiny hair cells (called cilia) that transmit sound through the ear are injured. This type of hearing loss is sometimes called "nerve damage," although this is not accurate [77].

Table S2. Search strategies and results

Engine	Strategy	Cites
**PubMed**	("Otitis Media"[Mesh] OR Otitis media OR "Otitis Media/epidemiology"[Mesh]) AND (("Epidemiology"[Mesh] OR epidemiology) OR ("Prevalence"[Mesh] OR prevalence) OR ("Incidence"[Mesh] OR incidence) OR "Epidemiologic Studies"[Mesh] OR ("Mortality"[Mesh] OR mortality OR fatality) OR ("Morbidity"[Mesh] OR morbidity) OR case fatality OR sequela* OR risk factor*)<br>Limits: 01/01/1980 to 11/08/2008	**5736**
**Embase**	(“Otitis media” [Emtree] OR Otitis media.mp) AND ((Epidemiology [Emtree] OR Epidemiology.mp) OR (Prevalence [Emtree] OR prevalence.mp) OR (Incidence [Emtree] OR incidence.mp) OR Epidemiologic studies.mp OR (Mortality [Emtree] OR Mortality.mp) OR (Fatality [Emtree] OR case fatality.mp) OR sequela*.mp OR risk factor*.mp)<br>Limits: 01/01/1980 to 23/07/2008	**3455**
**Lilacs**	Otitis AND epidemiology (**Results 74**)<br>Otitis AND mortality (**Results 11**)<br>Otitis AND morbidity (**Results 7**)<br>Otitis AND prevalence (**Results 19**)<br>Otitis AND incidence (**Results 15**)<br>Otitis AND sequela$ (**Results 9**)<br>Otitis AND fatality (**Results 0**)<br>Otitis AND risk factor$ (**Results 21**)	**156**
**WHOLIS**	Otitis media OR otitis	**9**

Table S3. WHO grades of hearing impairment

Grade of Impairment (better ear)	Audiometric ISO value (average of 0.5, 1, 2, 4 kHz)	Impairment description
No impairment	≤25 dBHL	No or very slight hearing problems. Able to hear whispers
Slight impairment	26-40 dBHL	Able to hear and repeat words spoken in normal voice at 1 metre
Moderate impairment	41-60 dBHL	Able to hear and repeat words using raised voice at 1 metre
Severe impairment	61-80 dBHL	Able to hear some words when shouted into better ear
Profound impairment	>80 dBHL	Unable to hear and understand even including a shouted voice

Source: [61,62]

Reference List

1. Mathers CD, Lopez AD, Murray CJL (2006) The burden of disease and mortality by condition: data, methods and results for 2001. In: Lopez AD, Mathers CD, Ezzati M, Jamison DT, Murray CJL, editors. Global Burden of Disease and Risk Factors. Washington (DC): World Bank. pp. 45-240.
2. Little P, Gould C, Williamson I, Moore M, Warner G, Dunleavey J (2001) Pragmatic randomised controlled trial of two prescribing strategies for childhood acute otitis media. BMJ 322: 336-342.
3. NICE Short Clinical Guidelines Technical Team (2008) Respiratory tract infections – antibiotic prescribing. Prescribing of antibiotics for self-limiting respiratory tract infections in adults and children in primary care. London: National Institute for Health and Clinical Excellence.
4. Mandel EM, Doyle WJ, Winther B, Alper CM (2008) The incidence, prevalence and burden of OM in unselected children aged 1-8 years followed by weekly otoscopy through the "common cold" season. Int J Pediatr Otorhinolaryngol 72: 491-499.
5. Osma U, Cureoglu S, Hosoglu S (2000) The complications of chronic otitis media: report of 93 cases. J Laryngol Otol 114: 97-100.
6. Cusimano F, Cocita VC, D'Amico A (1989) Sensorineural hearing loss in chronic otitis media. J Laryngol Otol 103: 158-163.
7. Sade J, Halevy A (1976) The natural history of chronic otitis media. J Laryngol Otol 90: 743-751.
8. Papp Z, Rezes S, Jokay I, Sziklai I (2003) Sensorineural hearing loss in chronic otitis media. Otol Neurotol 24: 141-144.
9. Tüz M, Dogru H, Uygur K, Aynali G (2006) Sensorineural Hearing Loss Associated with Chronic Otitis Media. S D Ü Týp Fak Derg 13: 1-2.
10. Fernandes de Azevedo A, Pinto DC, de Souza NJ, Greco DB, Goncalves DU (2007) Sensorineural hearing loss in chronic suppurative otitis media with and without cholesteatoma. Braz J Otorhinolaryngol 73: 671-674.
11. Harvard University, Institute for Health Metrics and Evaluation at the University of Washington, Johns Hopkins University, University of Queensland, World Health Organization (2009) GBD Study Operations Manual – Final Draft Jan. 2009.
12. Uhari M, Mantysaari K, Niemela M (1996) A meta-analytic review of the risk factors for acute otitis media. Clin Infect Dis 22: 1079-1083.
13. Ip S, Chung M, Raman G, Chew P, Magula N, DeVine D, Trikalinos T, Lau J (2007) Breastfeeding and maternal and infant health outcomes in developed countries. Evid Rep Technol Assess (Full Rep) 1-186.

14. Lasisi AO, Olaniyan FA, Muibi SA, Azeez IA, Abdulwasiu KG, Lasisi TJ, Imam ZO, Yekinni TO, Olayemi O (2007) Clinical and demographic risk factors associated with chronic suppurative otitis media. Int J Pediatr Otorhinolaryngol 71: 1549-1554.
15. World Health Organization (2008) World Health Statistics 2008.
16. World Health Organization (2009) World Health Statistics 2009.
17. UNICEF (2006) The State of the World's Children 2007: The Double Dividend of Gender Equality.
18. OECD (2008) OECD in Figures 2008. Available: http://www.oecd.org/dataoecd/44/17/41733586.pdf. Accessed 2012 Mar 19.
19. OECD (2008) OECD Health Data 2008. Available: http://stats.oecd.org/index.aspx?DataSetCode=HEALTH_STAT. Accessed 2012 Mar 19.
20. Joki-Erkkila VP, Laippala P, Pukander J (1998) Increase in paediatric acute otitis media diagnosed by primary care in two Finnish municipalities--1994-5 versus 1978-9. Epidemiol Infect 121: 529-534.
21. Garces-Sanchez M, ez-Domingo J, varez de LT, Planelles V, Graullera M, Baldo JM, Garcia Llop LA, Garcia LM, Peris VA, Gallego G, Ballester SA, Peidro C, Villarroya J, JuBert A, Colomer RJ, Casani C (2004) [Epidemiology and burden of acute otitis media in Valencia (Spain)]. An Pediatr (Barc) 60: 125-132.
22. Appelman CLM, van Balen FAM, van de Lisdonk EH, van Weert HCLM, Eizenga WH (1999) NHG-standaard Otitis media acuta (eerste herziening). Huisarts Wet 42: 362-366.
23. Cohen D, Tamir D (1989) The prevalence of middle ear pathologies in Jerusalem school children. Am J Otol 10: 456-459.
24. Podoshin L, Fradis M, Ben-David Y, Margalit A, Tamir A, Epstein L (1986) Cholesteatoma: an epidemiologic study among members of kibbutzim in northern Israel. Ann Otol Rhinol Laryngol 95: 365-368.
25. Virolainen E, Puhakka H, Aantaa E, Tuohimaa P, Ruuskanen O, Meurman OH (1980) Prevalence of secretory otitis media in seven to eight year old school children. Ann Otol Rhinol Laryngol Suppl 89: 7-10.
26. Berzon DB (1983) Ear disease in a group general practice. A review of world communities. J Laryngol Otol 97: 817-824.
27. Browning GG, Gatehouse S (1992) The prevalence of middle ear disease in the adult British population. Clin Otolaryngol Allied Sci 17: 317-321.
28. Pedersen CB, Zachau-Christiansen B (1988) Chronic otitis media and sequelae in the population of Greenland. Scand J Soc Med 16: 15-19.
29. Homoe P, Christensen RB, Bretlau P (1996) Prevalence of otitis media in a survey of 591 unselected Greenlandic children. Int J Pediatr Otorhinolaryngol 36: 215-230.

30. Homoe P (1999) Present aspects of otitis media among children in Greenland. Int J Pediatr Otorhinolaryngol 49 Suppl 1:S169-72.: S169-S172.
31. Klein JO, Teele DW, Rosner B, Allen C, Bratton L, Fisch G, Stringham P, Starobin S, Tarlin L (1990) Epidemiology of otitis media in Boston children: Observed from birth to 7 years of age. Annals of Otology, Rhinology and Laryngology 99(7 II SUPPL 149)()(pp 27-28), 1990 Date of Publication: 1990 27-28.
32. Teele DW, Klein JO, Rosner BA (1980) Epidemiology of otitis media in children. Ann Otol Rhinol Laryngol Suppl 89: 5-6.
33. Casselbrant ML, Mandel EM, Kurs-Lasky M, Rockette HE, Bluestone CD (1995) Otitis media in a population of black American and white American infants, 0-2 years of age. Int J Pediatr Otorhinolaryngol 33: 1-16.
34. Teele DW, Klein JO, Rosner B (1989) Epidemiology of otitis media during the first seven years of life in children in greater Boston: a prospective, cohort study. J Infect Dis 160: 83-94.
35. Kim CS, Jung HW, Yoo KY (1993) Prevalence of otitis media and allied diseases in Korea--results of a nation-wide survey, 1991. J Korean Med Sci 8: 34-40.
36. Noh KT, Kim CS (1985) The changing pattern of otitis media in Korea. Int J Pediatr Otorhinolaryngol 9: 77-87.
37. Mackenzie GA, Carapetis JR, Leach AJ, Morris PS (2009) Pneumococcal vaccination and otitis media in Australian Aboriginal infants: comparison of two birth cohorts before and after introduction of vaccination. BMC Pediatr 9: 14.
38. Matanda RN, Muyunga KC, Sabue MJ, Creten W, Van De HP (2005) Chronic suppurative otitis media and related complications at the University Clinic of Kinshasa. B-ENT 1: 57-62.
39. Nel M, Odendaal W, Hurter M, Meyer S, van der MA (1988) [The prevalence and nature of hearing problems and middle ear dysfunctions in a group of black urban children in grade 1]. S Afr J Commun Disord 35:25-9.: 25-29.
40. Prescott CA, Kibel MA (1991) Ear and hearing disorders in rural grade 2 (Sub B) schoolchildren in the Western Cape. S Afr Med J %19;79: 90-93.
41. Kamal N, Joarder AH, Chowdhury AA, Khan AW (2004) Prevalence of chronic suppurative otitis media among the children living in two selected slums of Dhaka City. Bangladesh Med Res Counc Bull 30: 95-104.
42. Biswas AC, Joarder AH, Siddiquee BH (2005) Prevalence of CSOM among rural school going children. Mymensingh Med J 14: 152-155.
43. Mann SB, Sharma SC, Gupta AK, Nagarkar AN, Dharamvir (1998) Incidence of hearing impairment among rural and urban school going children: a survey. Indian J Pediatr 65: 141-145.
44. Roy E, Hasan K, Haque F, Siddique AK, Sack RB (2007) Acute otitis media during the first two years of life in a rural community in Bangladesh: a prospective cohort study. J Health Popul Nutr 25: 414-421.

45. Zakzouk SM, Hajjaj MF (2002) Epidemiology of chronic suppurative otitis media among Saudi children--a comparative study of two decades. Int J Pediatr Otorhinolaryngol 62: 215-218.
46. Zakzouk SM, Jamal TS, Daghistani KJ (2002) Epidemiology of acute otitis media among Saudi children. Int J Pediatr Otorhinolaryngol 62: 219-222.
47. Abolfotouh MA, Ghieth MM, Badawi IA (1995) Hearing loss and other ear problems among schoolboys in Abha, Saudi Arabia. Ann Saudi Med 15: 323-326.
48. Balle VH, Tos M, Dang HS, Nhan TS, Le T, Tran KP, Tran TT, Vu MT (2000) Prevalence of chronic otitis media in a randomly selected population from two communes in southern Vietnam. Acta Otolaryngol Suppl 543:51-3.: 51-53.
49. Chayarpham S, Stuart J, Chongsuvivatwong V, Chinpairoj S, Lim A (1996) A study of the prevalence of and risk factors for ear diseases and hearing loss in primary school children in Hat Yai, Thailand. J Med Assoc Thai 79: 468-472.
50. Elango S, Purohit GN, Hashim M, Hilmi R (1991) Hearing loss and ear disorders in Malaysian school children. Int J Pediatr Otorhinolaryngol 22: 75-80.
51. Antarasena S, Antarasena N, Lekagul S, Lutadul D (1988) The epidemiology of deafness in Thailand. Otolaryngol Head Neck Surg 3: 9-13.
52. Acuin, J. (2004) Chronic suppurative otitis media - Burden of Illness and Management Options. Geneva: World Health Organization.
53. Minja BM, Machemba A (1996) Prevalence of otitis media, hearing impairment and cerumen impaction among school children in rural and urban Dar es Salaam, Tanzania. Int J Pediatr Otorhinolaryngol 37: 29-34.
54. Amusa YB, Ijadunola IK, Onayade OO (2005) Epidemiology of otitis media in a local tropical African population. West Afr J Med 24: 227-230.
55. Lasisi AO, Sulaiman OA, Afolabi OA (2007) Socio-economic status and hearing loss in chronic suppurative otitis media in Nigeria. Ann Trop Paediatr 27: 291-296.
56. Swart SM, Lemmer R, Parbhoo JN, Prescott CA (1995) A survey of ear and hearing disorders amongst a representative sample of grade 1 schoolchildren in Swaziland. Int J Pediatr Otorhinolaryngol 32: 23-34.
57. Lyn C, Jadusingh WA, Ashman H, Chen D, Abramson A, Soutar I (1998) Hearing screening in Jamaica: prevalence of otitis media with effusion. Laryngoscope 108: 288-290.
58. Godinho RN, Goncalves TM, Nunes FB, Becker CG, Becker HM, Guimaraes RE, Sanfins F, Colosimo EA, Oliveira RG, Lamounier JA (2001) Prevalence and impact of chronic otitis media in school age children in Brazil.

First epidemiologic study concerning chronic otitis media in Latin America. Int J Pediatr Otorhinolaryngol 61: 223-232.

59. Manjarrez ME, Rosete DP, Rincon M, Villalba J, Cravioto A, Cabrera R (2003) Comparative viral frequency in Mexican children under 5 years of age with and without upper respiratory symptoms. J Med Microbiol 52: 579-583.
60. Arevalo-Silva CA, Villasenor-Sierra A, Kuri-Morales P, Tapia-Conyer R, Santos-Preciado JI (1999) [Acute otitis media in Mexico: cases reported during the period from 1995 to 1998]. Gac Med Mex 135: 541-543.
61. WHO-PDH (1991) Report of the Informal Working Group on Prevention of Deafness and Hearing Impairment, Programme Planning. Geneva, 18-21 June 1991. WHO/PDH/91.1.
62. Mathers CD, Smith A, Concha M (2003) Global Burden of hearing loss in the year 2000 - GBD 2000 Working Paper.
63. Ashoor A (1983) Hearing levels of school children in Dammam. J Laryngol Otol 97: 37-41.
64. Abdel-Hamid O, Khatib OM, Aly A, Morad M, Kamel S (2007) Prevalence and patterns of hearing impairment in Egypt: a national household survey. East Mediterr Health J 13: 1170-1180.
65. Giles M, O'Brien P (1991) The prevalence of hearing impairment amongst Maori schoolchildren. Clin Otolaryngol Allied Sci 16: 174-178.
66. Vartiainen E, Vartiainen J (1995) Age and hearing function in patients with chronic otitis media. J Otolaryngol 24: 336-339.
67. Al Khabori M, Khandekar R (2004) The prevalence and causes of hearing impairment in Oman: a community-based cross-sectional study. Int J Audiol 43: 486-492.
68. Liu C, Bu XK, Xing GQ, Zhou L, Xu X, Wang DY, Chen ZB, Zhou H, Tian HQ, Li XL, Lu L, Zhao XN, Li FL, Tan CQ (2006) [Epidemiologic study on hearing impairment and ear diseases in old people]. Zhonghua Er Bi Yan Hou Tou Jing Wai Ke Za Zhi 41: 661-664.
69. Bansal R, Raj A (1998) Hearing loss in rural population: The etiology. Indian Journal of Otolaryngology and Head and Neck Surgery 50(2)()(pp 147-155), 1998 Date of Publication: 1998 147-155.
70. Jacob A, Rupa V, Job A, Joseph A (1997) Hearing impairment and otitis media in a rural primary school in south India. Int J Pediatr Otorhinolaryngol 39: 133-138.
71. SIGN (2003) Diagnosis and management of childhood otitis media in primary care - A national clinical guideline.
72. Gates GA, Klein JO, Lim DJ, Mogi G, Ogra PL, Pararella MM, Paradise JL, Tos M (2002) Recent advances in otitis media. 1. Definitions, terminology, and classification of otitis media. Ann Otol Rhinol Laryngol Suppl 188: 8-18.

73. Rovers MM, Schilder AG, Zielhuis GA, Rosenfeld RM (2004) Otitis media. Lancet 363: 465-473.

74. Gungor A, Bluestone CD (2001) Antibiotic theory in otitis media. Curr Allergy Asthma Rep 1: 364-372.

75. NICE (2000) Persistent otitis media with effusion (glue ear) in young children (Referral practice project - Version under pilot).

76. Alberta Clinical Practice Guidelines Working Group (2000) Diagnosis and Treatment of Acute Otitis Media in Children - 2008 Update.

77. Medline plus enciclopedia (2011 November) Hearing loss. National Library of Medicine - National Institutes of Health. Available: http://www.nlm.nih.gov/medlineplus/ency/article/003044.htm. Accessed 2012 Mar 19.

78. WHO (2001) International classification of functioning, disability and health: ICF.
