# Supplementary figures and images for: Burden of Disease Caused by Otitis Media: Systematic Review and Global Estimates
Source: PLoS One. 2012 Apr 30;7(4):e36226. doi: 10.1371/journal.pone.0036226 (PMC3340347; doi:10.1371/journal.pone.0036226)

**Figure S2. Sequelae of OM, simplified scheme**

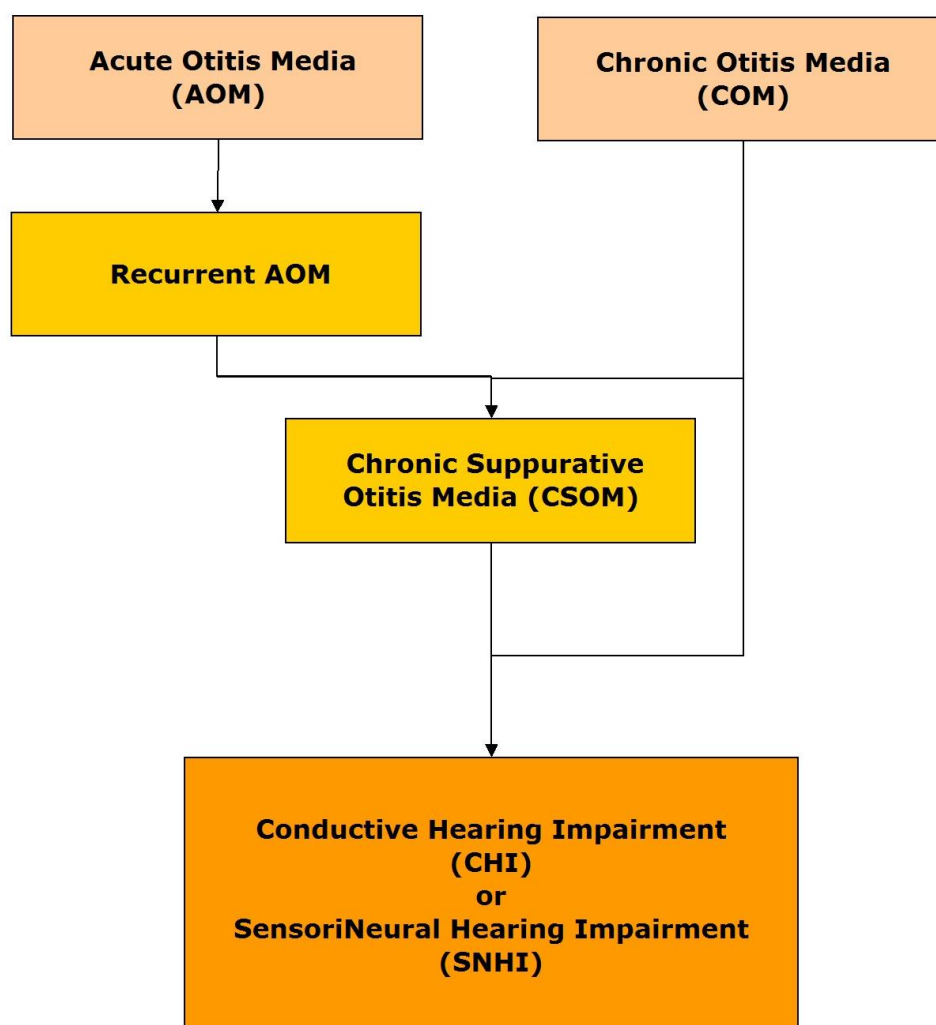

Supplement: Figure S1 — Sequelae of OM, simplified scheme. (PDF) [file pone.0036226.s002.pdf]

Figure S3. 1<sup>st</sup> screening by titles, abstract and keywords

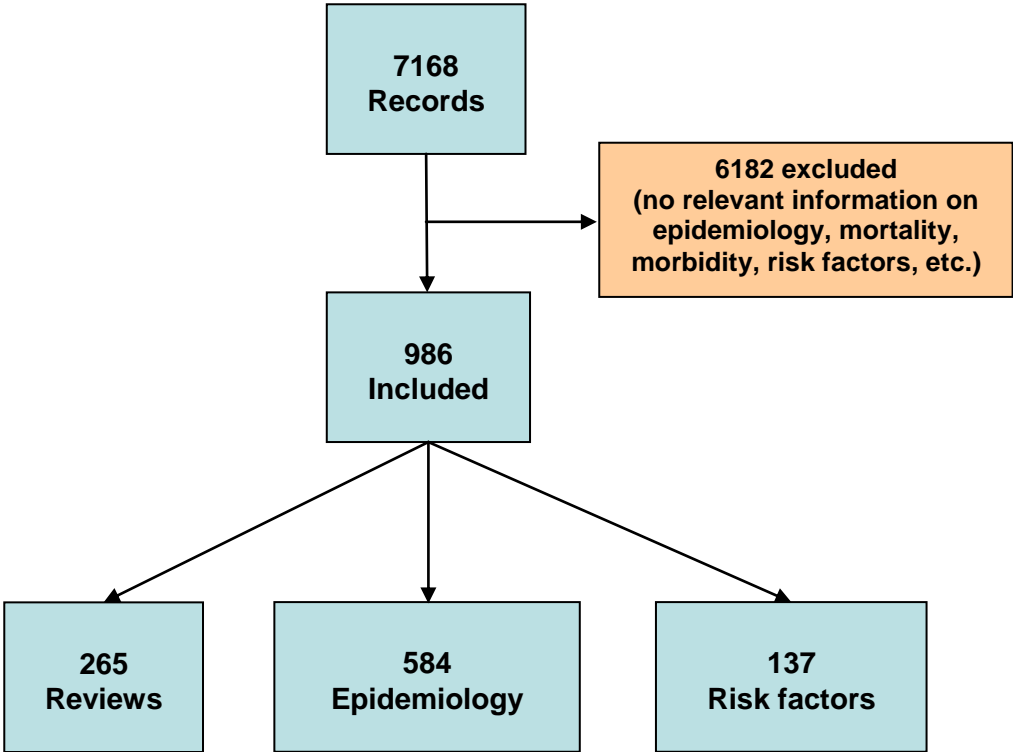

Supplement: Figure S2 — 1st screening by titles, abstract and keywords. (PDF) [file pone.0036226.s003.pdf]

Figure S4. Risk factors diagram

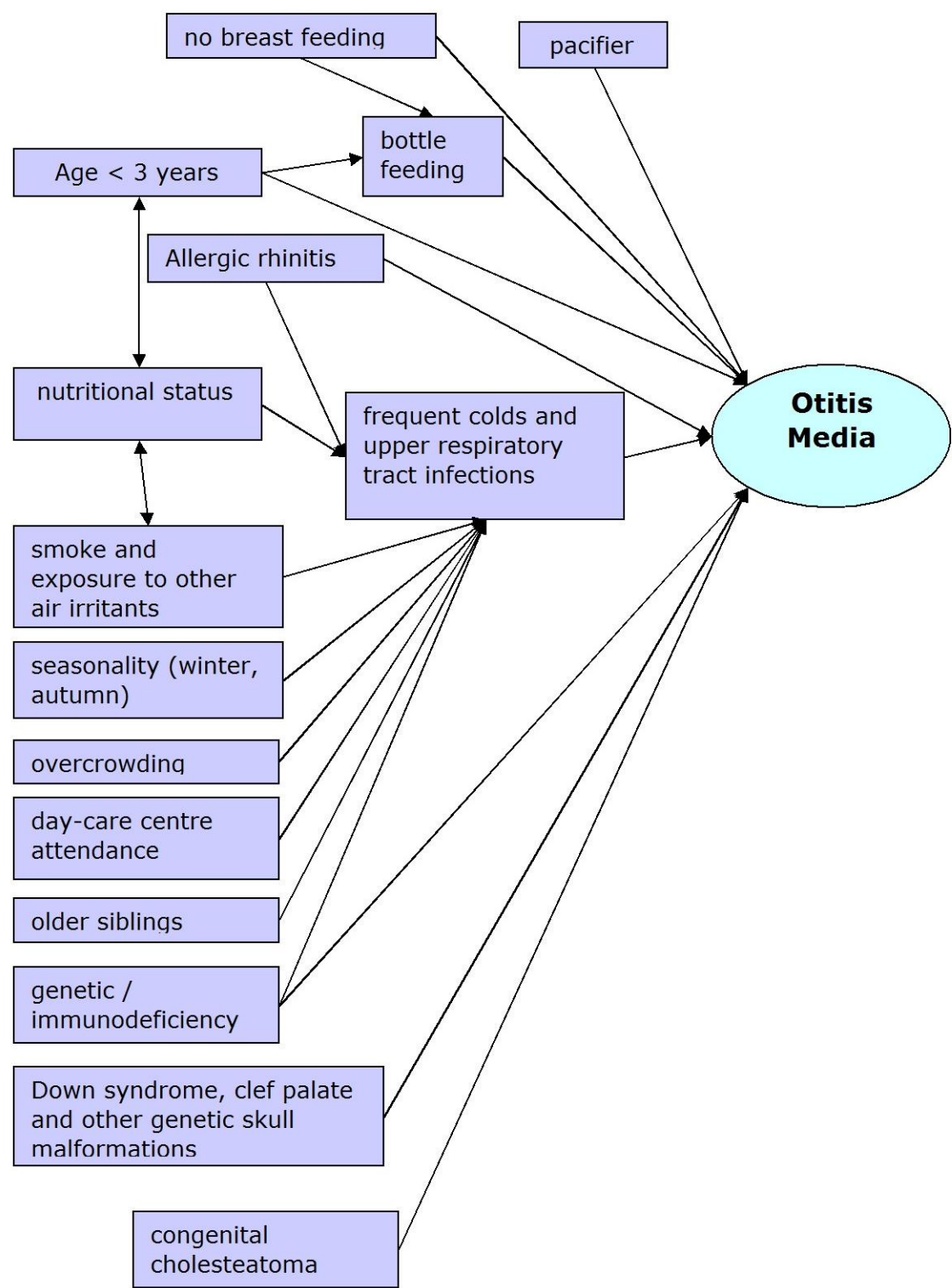

Supplement: Figure S3 — Risk factor diagram. (PDF) [file pone.0036226.s004.pdf]
