# Supplementary material for: Burden of Disease Caused by Otitis Media: Systematic Review and Global Estimates
Source: PLoS One. 2012 Apr 30;7(4):e36226. doi: 10.1371/journal.pone.0036226 (PMC3340347; doi:10.1371/journal.pone.0036226)
Supplement: Table S1 — Search strategies and results. (PDF) [file pone.0036226.s005.pdf]

Table S2. Search strategies and results

Engine	Strategy	Cites
**PubMed**	("Otitis Media"[Mesh] OR Otitis media OR "Otitis Media/epidemiology"[Mesh]) AND (("Epidemiology"[Mesh] OR epidemiology) OR ("Prevalence"[Mesh] OR prevalence) OR ("Incidence"[Mesh] OR incidence) OR "Epidemiologic Studies"[Mesh] OR ("Mortality"[Mesh] OR mortality OR fatality) OR ("Morbidity"[Mesh] OR morbidity) OR case fatality OR sequela* OR risk factor*)<br>Limits: 01/01/1980 to 11/08/2008	**5736**
**Embase**	("Otitis media" [Emtree] OR Otitis media.mp) AND ((Epidemiology [Emtree] OR Epidemiology.mp) OR (Prevalence [Emtree] OR prevalence.mp) OR (Incidence [Emtree] OR incidence.mp) OR Epidemiologic studies.mp OR (Mortality [Emtree] OR Mortality.mp) OR (Fatality [Emtree] OR case fatality.mp) OR sequela*.mp OR risk factor*.mp)<br>Limits: 01/01/1980 to 23/07/2008	**3455**
**Lilacs**	Otitis AND epidemiology (**Results 74**)<br>Otitis AND mortality (**Results 11**)<br>Otitis AND morbidity (**Results 7**)<br>Otitis AND prevalence (**Results 19**)<br>Otitis AND incidence (**Results 15**)<br>Otitis AND sequela$ (**Results 9**)<br>Otitis AND fatality (**Results 0**)<br>Otitis AND risk factor$ (**Results 21**)	**156**
**WHOLIS**	Otitis media OR otitis	**9**
