# Supplementary material for: Burden of Disease Caused by Otitis Media: Systematic Review and Global Estimates
Source: PLoS One. 2012 Apr 30;7(4):e36226. doi: 10.1371/journal.pone.0036226 (PMC3340347; doi:10.1371/journal.pone.0036226)
Supplement: Table S2 — WHO grades of hearing impairment. (PDF) [file pone.0036226.s006.pdf]

Table S3. WHO grades of hearing impairment

Grade of Impairment (better ear)	Audiometric ISO value (average of 0.5, 1, 2, 4 kHz)	Impairment description
No impairment	≤25 dBHL	No or very slight hearing problems. Able to hear whispers
Slight impairment	26-40 dBHL	Able to hear and repeat words spoken in normal voice at 1 metre
Moderate impairment	41-60 dBHL	Able to hear and repeat words using raised voice at 1 metre
Severe impairment	61-80 dBHL	Able to hear some words when shouted into better ear
Profound impairment	>80 dBHL	Unable to hear and understand even including a shouted voice

Source:

- WHO-PDH (1991) Report of the Informal Working Group on Prevention of Deafness and Hearing Impairment, Programme Planning. Geneva, 18-21 June 1991. WHO/PDH/91.1.

- Mathers CD, Smith A, Concha M (2003) Global Burden of hearing loss in the year 2000 - GBD 2000 Working Paper.
