# Supplementary material for: Burden of Disease Caused by Otitis Media: Systematic Review and Global Estimates
Source: PLoS One. 2012 Apr 30;7(4):e36226. doi: 10.1371/journal.pone.0036226 (PMC3340347; doi:10.1371/journal.pone.0036226)
Supplement: Table S3 — Data coverage. (PDF) [file pone.0036226.s007.pdf]

Table S4. Data coverage

**Regions**	**AOM** Incidence or prevalence	**CSOM** Incidence or prevalence	**% CSOM causing HI**	**% HI caused by OM**	**Regions with data**
Asia Pacific, High Income	1	2	0	0	yes
Asia, Central	0	0	0	0	no
Asia, East	1	1	0	1	yes
Asia, South	0	2	1	1	yes
Asia, Southeast	0	5	1	3	yes
Australasia	2	3	0	0	yes
Caribbean	0	1	1	1	yes
Europe, Central	0	0	0	0	no
Europe, Eastern	0	0	0	0	no
Europe, Western	20	8	2	5	yes
Latin America, Andean	0	0	0	0	no
Latin America, Central	0	0	0	0	no
Latin America, Southern	0	0	0	0	no
Latin America, Tropical	0	2	1	2	yes
North Africa / Middle East	2	5	2	7	yes
North America, High Income	12	6	1	1	yes
Oceania	0	3	0	0	yes
Sub-Saharan Africa, Central	0	2	2	2	yes
Sub-Saharan Africa, East	0	5	4	1	yes
Sub-Saharan Africa, Southern	0	2	1	0	yes
Sub-Saharan Africa, West	0	2	0	2	yes
**Regions covered**	6	15	10	11	15
**% Regions covered**	**29%**	**71%**	**48%**	**52%**	**71%**
