# Supplementary material for: Burden of Disease Caused by Otitis Media: Systematic Review and Global Estimates
Source: PLoS One. 2012 Apr 30;7(4):e36226. doi: 10.1371/journal.pone.0036226 (PMC3340347; doi:10.1371/journal.pone.0036226)
Supplement: Table S4 — AOM and CSOM incidence and prevalence, HI cases and prevalence estimates, and Mortality estimates, by age groups and WHO region. (PDF) [file pone.0036226.s008.pdf]

Table S5. AOM incidence and prevalence estimates for the year 2005, by age group

		incidence: cases			incidence %			prevalence: cases			prevalence %		
Age Groups	Total pop.	AOM	MIN	MAX	AOM	MIN	MAX	AOM	MIN	MAX	AOM	MIN	MAX
0-11m.	127842788	57887310	57051574	58721916	45.28	44.63	45.93	3330503	3282419	3378521	2.61	2.57	2.64
1 - 4	497107358	303180190	299874049	306486330	60.99	60.32	61.65	17443243	17253029	17633460	3.51	3.47	3.55
5 - 9	610073590	135149474	131233936	139085467	22.15	21.51	22.80	7775723	7550446	8002178	1.27	1.24	1.31
10 - 14	613180667	113451292	109527558	117437511	18.50	17.86	19.15	6527335	6301586	6756679	1.06	1.03	1.10
15 - 19	610164733	21524535	17685883	25373212	3.53	2.90	4.16	1238398	1017544	1459829	0.20	0.17	0.24
20 - 24	560473407	17608587	14121144	21109373	3.14	2.52	3.77	1013097	812449	1214512	0.18	0.14	0.22
25 - 34	1007681602	15699065	9595138	21868114	1.56	0.95	2.17	903234	552049	1258165	0.09	0.05	0.12
35 - 44	888222451	13222874	8012655	18492279	1.49	0.90	2.08	760768	461002	1063939	0.09	0.05	0.12
45 - 54	689115655	12184709	8254564	16163327	1.77	1.20	2.35	701038	474920	929945	0.10	0.07	0.13
55 - 64	449003044	8618737	6169089	11102067	1.92	1.37	2.47	495873	354934	638749	0.11	0.08	0.14
65 - 74	298397449	6275514	4682424	7893936	2.10	1.57	2.65	361057	269400	454172	0.12	0.09	0.15
75 - 84	146420903	3429091	2720733	4145357	2.34	1.86	2.83	197290	156535	238500	0.13	0.11	0.16
85 +	34019794	772037	634468	910165	2.27	1.86	2.68	44419	36504	52366	0.13	0.11	0.15
Total	6531703441	709003415	669563215	748789054	10.85	10.25	11.46	40791978	38522817	43081015	0.62	0.59	0.66

Table S6. AOM incidence and prevalence estimates for the year 2005, by WHO region

		incidence: cases			incidence %			prevalence: cases			prevalence %		
Age Groups	Total pop.	AOM	MIN	MAX	AOM	MIN	MAX	AOM	MIN	MAX	AOM	MIN	MAX
Asia Central	76814553	6068805	5541469	6596141	7.90	7.21	8.59	349164	318824	379504	0.45	0.42	0.49
Asia East	1366340458	53636834	44256866	63016802	3.93	3.24	4.61	3085955	2546285	3625624	0.23	0.19	0.27
Asia Pacific High Income	180467879	6769987	5977605	7562369	3.75	3.31	4.19	389506	343917	435095	0.22	0.19	0.24
Asia South	1498562845	217626134	207338454	227913814	14.52	13.84	15.21	12520956	11929062	13112850	0.84	0.80	0.88
Asia South East	573902462	46796257	42856399	50736115	8.15	7.47	8.84	2692387	2465711	2919064	0.47	0.43	0.51
Australasia	24407320	1769477	1662313	1876641	7.25	6.81	7.69	101806	95640	107971	0.42	0.39	0.44
Caribbean	38179725	3468044	3205937	3730151	9.08	8.40	9.77	199531	184451	214611	0.52	0.48	0.56
Europe Central	118749415	4317040	3501820	5132260	3.64	2.95	4.32	248378	201475	295281	0.21	0.17	0.25
Europe Eastern	211613757	8387849	6935113	9840585	3.96	3.28	4.65	482589	399007	566171	0.23	0.19	0.27
Europe Western	407712685	24108710	23963498	24409276	5.91	5.88	5.99	1387076	1378722	1404369	0.34	0.34	0.34
Latin America Andean	49517274	2666824	2485455	3006763	5.39	5.02	6.07	153434	142999	172992	0.31	0.29	0.35
Latin America Central	215171064	14597800	13120644	16074956	6.78	6.10	7.47	839873	754886	924860	0.39	0.35	0.43
Latin America Southern	58370932	2478569	2077850	2879288	4.25	3.56	4.93	142603	119548	165658	0.24	0.20	0.28
Latin America Tropical	192735096	11369047	10045913	12692181	5.90	5.21	6.59	654110	577984	730235	0.34	0.30	0.38
North Africa Middle East	410799625	35606938	32786787	38427089	8.67	7.98	9.35	2048618	1886363	2210874	0.50	0.46	0.54
North America High Income	332116959	18121701	17922615	18352301	5.46	5.40	5.53	1042618	1031164	1055886	0.31	0.31	0.32
Oceania	9003928	2571268	2509457	2633080	28.56	27.87	29.24	147936	144380	151492	1.64	1.60	1.68
Sub-Saharan Africa Central	84411831	36612439	36032948	37191930	43.37	42.69	44.06	2106469	2073129	2139810	2.50	2.46	2.53
Sub-Saharan Africa East	314207088	71678006	69520965	73835047	22.81	22.13	23.50	4123940	3999836	4248044	1.31	1.27	1.35
Sub-Saharan Africa Southern	68019317	10004695	9537739	10471651	14.71	14.02	15.40	575613	548747	602479	0.85	0.81	0.89
Sub-Saharan Africa West	300599228	130346991	128283368	132410614	43.36	42.68	44.05	7499416	7380687	7618145	2.49	2.46	2.53
Total	6531703441	709003415	669563215	748789054	10.85	10.25	11.46	40791978	38522817	43081015	0.62	0.59	0.66

Table S7. CSOM incidence and prevalence estimates for the year 2005, by age group

		incidence: cases			incidence ‰			prevalence: cases			prevalence %		
Age Groups	Total pop.	CSOM	MIN	MAX	CSOM	MIN	MAX	CSOM	MIN	MAX	CSOM	MIN	MAX
0-11m.	127842788	1968151	1895048	2041363	15.40	14.82	15.97	1968151	1895048	2041363	1.54	1.48	1.60
1 - 4	497107358	5036076	4766368	5305784	10.13	9.59	10.67	10072152	9532736	10611568	2.03	1.92	2.13
5 - 9	610073590	5067956	4742184	5393650	8.31	7.77	8.84	15203868	14226552	16180950	2.49	2.33	2.65
10 - 14	613180667	2383190	2056140	2718544	3.89	3.35	4.43	14299140	12336840	16311264	2.33	2.01	2.66
15 - 19	610164733	2053198	1757736	2348038	3.36	2.88	3.85	16425584	14061888	18784304	2.69	2.30	3.08
20 - 24	560473407	2692119	2382773	3014719	4.80	4.25	5.38	24229071	21444957	27132471	4.32	3.83	4.84
25 - 34	1007681602	3331502	2824815	3848260	3.31	2.80	3.82	33315020	28248150	38482600	3.31	2.80	3.82
35 - 44	888222451	2819138	2405255	3233168	3.17	2.71	3.64	28191380	24052550	32331680	3.17	2.71	3.64
45 - 54	689115655	2825118	2492837	3167735	4.10	3.62	4.60	28251180	24928370	31677350	4.10	3.62	4.60
55 - 64	449003044	1681031	1500800	1857028	3.74	3.34	4.14	16810310	15008000	18570280	3.74	3.34	4.14
65 - 74	298397449	736199	633790	827445	2.47	2.12	2.77	7361990	6337900	8274450	2.47	2.12	2.77
75 - 84	146420903	373503	333585	406220	2.55	2.28	2.77	3735030	3335850	4062200	2.55	2.28	2.77
85 +	34019794	92781	87161	96305	2.73	2.56	2.83	927810	871610	963050	2.73	2.56	2.83
**Total**	6531703441	31059962	27878492	34258259	4.76	4.27	5.24	200790686	176280451	225423530	3.07	2.70	3.45

Table S8. CSOM incidence and prevalence estimates for the year 2005, by WHO region

		incidence: cases			incidence ‰			prevalence: cases			prevalence %		
Age Groups	Total pop.	CSOM	MIN	MAX	CSOM	MIN	MAX	CSOM	MIN	MAX	CSOM	MIN	MAX
Asia Central	76814553	310847	271431	350263	4.05	3.53	4.56	2209932	1900943	2518921	2.88	2.47	3.28
Asia East	1366340458	5013119	4312016	5714222	3.67	3.16	4.18	40535129	34650169	46420089	2.97	2.54	3.40
Asia Pacific High Income	180467879	545552	391625	699479	3.02	2.17	3.88	5207268	3843553	6570983	2.89	2.13	3.64
Asia South	1498562845	9836102	9067155	10605049	6.56	6.05	7.08	51799831	46021184	57578478	3.46	3.07	3.84
Asia South East	573902462	2689060	2394576	2983544	4.69	4.17	5.20	17580843	15272156	19889530	3.06	2.66	3.47
Australasia	24407320	83254	62435	104073	3.41	2.56	4.26	714409	536721	892097	2.93	2.20	3.66
Caribbean	38179725	159712	140120	179304	4.18	3.67	4.70	1102727	948889	1256565	2.89	2.49	3.29
Europe Central	118749415	437757	376824	498690	3.69	3.17	4.20	3665369	3131732	4199006	3.09	2.64	3.54
Europe Eastern	211613757	793905	685322	902488	3.75	3.24	4.26	6592017	5635731	7548303	3.12	2.66	3.57
Europe Western	407712685	1381059	1321302	1457514	3.39	3.24	3.57	12081664	11839973	12445064	2.96	2.90	3.05
Latin America Andean	49517274	84183	58776	109590	1.70	1.19	2.21	491416	298846	683986	0.99	0.60	1.38
Latin America Central	215171064	842595	732186	953004	3.92	3.40	4.43	5979687	5133479	6825895	2.78	2.39	3.17
Latin America Southern	58370932	210253	180301	240205	3.60	3.09	4.12	1652507	1409778	1895236	2.83	2.42	3.25
Latin America Tropical	192735096	720547	621649	819445	3.74	3.23	4.25	5442888	4661226	6224550	2.82	2.42	3.23
North Africa Middle East	410799625	1811965	1601174	2022756	4.41	3.90	4.92	11955539	10349550	13561528	2.91	2.52	3.30
North America High Income	332116959	1016120	935974	1096397	3.06	2.82	3.30	9031131	8614810	9448370	2.72	2.59	2.84
Oceania	9003928	84357	79737	88975	9.37	8.86	9.88	369706	336520	402874	4.11	3.74	4.47
Sub-Saharan Africa Central	84411831	637899	594587	681211	7.56	7.04	8.07	2816629	2530924	3102334	3.34	3.00	3.68
Sub-Saharan Africa East	314207088	1904673	1743448	2065898	6.06	5.55	6.57	9511032	8421794	10600270	3.03	2.68	3.37
Sub-Saharan Africa Southern	68019317	325479	290575	360383	4.79	4.27	5.30	2022952	1761735	2284169	2.97	2.59	3.36
Sub-Saharan Africa West	300599228	2171524	2017279	2325769	7.22	6.71	7.74	10028010	8980738	11075282	3.34	2.99	3.68
**Total**	6531703441	31059962	27878492	34258259	4.76	4.27	5.24	200790686	176280451	225423530	3.07	2.70	3.45

Table S9. HI cases estimates for the year 2005, by age group

		25dB > HI ≤ 40dB			40dB > HI ≤ 60dB			60dB > HI ≤ 80dB			80dB > HI			TOTAL (HI >25dB)		
Age Groups	Total pop.	HI	MIN	MAX	HI	MIN	MAX	HI	MIN	MAX	HI	MIN	MAX	HI	MIN	MAX
0-11m.	127842788	63833	57076	70974	30425	29665	31214	14880	14635	15128	10234	10113	10355	119372	111489	127671
1 - 4	497107358	610107	582557	637873	290490	287432	293548	140286	139333	141245	94752	94280	95225	1135635	1103602	1167891
5 - 9	610073590	870510	836436	904584	412480	408727	416233	194091	192937	195245	126371	125823	126921	1603452	1563923	1642983
10 - 14	613180667	907927	873680	942174	426973	423201	430745	195263	194136	196394	122158	121645	122673	1652321	1612662	1691986
15 - 19	610164733	910675	876596	944754	424603	420849	428357	189507	188421	190596	114611	114141	115083	1639396	1600007	1678790
20 - 24	560473407	930559	899255	961863	431701	428254	435148	189454	188476	190433	111777	111365	112190	1663491	1627350	1699634
25 - 34	1007681602	1743148	1686867	1799429	801114	794915	807313	343471	341784	345159	196319	195646	196993	3084052	3019212	3148894
35 - 44	888222451	1670798	1621193	1720403	758379	752914	763844	315610	314202	317019	172834	172310	173358	2917621	2860619	2974624
45 - 54	689115655	1403033	1364545	1441521	634334	630095	638573	259416	258364	260467	137226	136847	137604	2434009	2389851	2478165
55 - 64	449003044	1024455	999376	1049534	461860	459099	464621	189544	188890	190203	101398	101165	101631	1777257	1748530	1805989
65 - 74	298397449	786936	770271	803601	349397	347561	351233	138385	137974	138801	69696	69559	69834	1344414	1325365	1363469
75 - 84	146420903	378453	370277	386629	163662	162764	164560	61985	61804	62168	29029	28975	29082	633129	623820	642439
85 +	34019794	78450	76549	80351	33018	32809	33227	11887	11853	11924	5013	5006	5021	128368	126217	130523
Total	6531703441	11378884	11014678	11743690	5218436	5178285	5258616	2243779	2232809	2254782	1291418	1286875	1295970	20132517	19712647	20553058

Table S10. HI estimated cases for the year 2005, by WHO region

		25dB > HI ≤ 40dB			40dB > HI ≤ 60dB			60dB > HI ≤ 80dB			80dB > HI			TOTAL (HI >25dB)		
Age Groups	Total pop.	HI	MIN	MAX	HI	MIN	MAX	HI	MIN	MAX	HI	MIN	MAX	HI	MIN	MAX
Asia Central	76814553	45552	41261	49843	15433	14959	15907	3968	3849	4087	1118	1086	1150	66071	61155	70987
Asia East	1366340458	920639	844329	996949	311776	303369	320183	73671	71685	75657	17426	16955	17897	1323512	1236338	1410686
Asia Pacific High Income	180467879	28038	18004	38118	5641	4534	6750	666	536	796	77	63	91	34422	23137	45755
Asia South	1498562845	7486453	7402760	7570146	3937306	3928089	3946523	1919588	1914992	1924184	1198377	1195417	1201337	14541724	14441258	14642190
Asia South East	573902462	560416	528363	592469	206915	203386	210444	57640	56665	58615	16886	16603	17169	841857	805017	878697
Australasia	24407320	2830	1483	4193	457	309	606	38	25	51	3	5	3	3328	1822	4853
Caribbean	38179725	19278	17145	21411	6442	6207	6677	1720	1660	1780	510	495	525	27950	25507	30393
Europe Central	118749415	45970	39337	52603	11489	10759	12219	1828	1712	1944	290	272	308	59577	52080	67074
Europe Eastern	211613757	87231	75412	99050	21265	19963	22567	3240	3042	3438	491	463	519	112227	98880	125574
Europe Western	407712685	46250	23792	69020	7549	5057	10057	691	642	772	47	42	59	54537	29533	79908
Latin America Andean	49517274	13731	10965	16497	7567	7261	7873	3419	3281	3557	1546	1486	1606	26263	22993	29533
Latin America Central	215171064	85453	73436	97470	23149	21825	24473	4205	3966	4444	774	729	819	113581	99956	127206
Latin America Southern	58370932	22986	19725	26247	5945	5585	6305	986	926	1046	165	154	176	30082	26390	33774
Latin America Tropical	192735096	73248	62484	84012	18639	17455	19823	3010	2819	3201	485	454	516	95382	83212	107552
North Africa Middle East	410799625	241104	218161	264047	85812	83284	88340	21966	21322	22610	5704	5538	5870	354586	328305	380867
North America High Income	332116959	38453	20129	57003	7496	5463	9539	856	624	1089	98	72	124	46903	26288	67755
Oceania	9003928	27473	26967	27979	12607	12553	12661	4464	4444	4484	1579	1571	1587	46123	45535	46711
Sub-Saharan Africa Central	84411831	190686	185971	195401	51757	51239	52275	10069	9967	10171	2419	2396	2442	254931	249573	260289
Sub-Saharan Africa East	314207088	612979	595428	630530	261311	259376	263246	92361	91673	93049	35884	35617	36151	1002535	982094	1022976
Sub-Saharan Africa Southern	68019317	46038	42238	49838	14112	13693	14531	2947	2861	3033	626	607	645	63723	59399	68047
Sub-Saharan Africa West	300599228	784076	767288	800864	205768	203919	207617	36446	36118	36774	6913	6850	6976	1033203	1014175	1052231
Total	6531703441	11378884	11014678	11743690	5218436	5178285	5258616	2243779	2232809	2254782	1291418	1286875	1295970	20132517	19712647	20553058

Table S11. HI prevalence estimates for the year 2005 per hundred thousand ($^{0}/_{0000}$) by age group

Age Groups	Total pop.	25dB > HI ≤ 40dB			40dB > HI ≤ 60dB			60dB > HI ≤ 80dB			80dB > HI			TOTAL (HI >25dB)		
		HI	MIN	MAX	HI	MIN	MAX	HI	MIN	MAX	HI	MIN	MAX	HI	MIN	MAX
0-11m.	127842788	49.93	44.65	55.52	23.80	23.20	24.42	11.64	11.45	11.83	8.01	7.91	8.10	93.37	87.21	99.87
1 - 4	497107358	122.73	117.19	128.32	58.44	57.82	59.05	28.22	28.03	28.41	19.06	18.97	19.16	228.45	222.00	234.94
5 - 9	610073590	142.69	137.10	148.27	67.61	67.00	68.23	31.81	31.63	32.00	20.71	20.62	20.80	262.83	256.35	269.31
10 - 14	613180667	148.07	142.48	153.65	69.63	69.02	70.25	31.84	31.66	32.03	19.92	19.84	20.01	269.47	263.00	275.94
15 - 19	610164733	149.25	143.67	154.84	69.59	68.97	70.20	31.06	30.88	31.24	18.78	18.71	18.86	268.68	262.23	275.14
20 - 24	560473407	166.03	160.45	171.62	77.02	76.41	77.64	33.80	33.63	33.98	19.94	19.87	20.02	296.80	290.35	303.25
25 - 34	1007681602	172.99	167.40	178.57	79.50	78.89	80.12	34.09	33.92	34.25	19.48	19.42	19.55	306.05	299.62	312.49
35 - 44	888222451	188.11	182.52	193.69	85.38	84.77	86.00	35.53	35.37	35.69	19.46	19.40	19.52	328.48	322.06	334.90
45 - 54	689115655	203.60	198.01	209.18	92.05	91.44	92.67	37.64	37.49	37.80	19.91	19.86	19.97	353.21	346.80	359.62
55 - 64	449003044	228.16	222.58	233.75	102.86	102.25	103.48	42.21	42.07	42.36	22.58	22.53	22.63	395.82	389.42	402.22
65 - 74	298397449	263.72	258.14	269.31	117.09	116.48	117.71	46.38	46.24	46.52	23.36	23.31	23.40	450.54	444.16	456.93
75 - 84	146420903	258.47	252.89	264.05	111.78	111.16	112.39	42.33	42.21	42.46	19.83	19.79	19.86	432.40	426.05	438.76
85 +	34019794	230.60	225.01	236.19	97.06	96.44	97.67	34.94	34.84	35.05	14.74	14.71	14.76	377.33	371.01	383.67
Total	6531703441	174.21	168.63	179.80	79.89	79.28	80.51	34.35	34.18	34.52	19.77	19.70	19.84	308.23	301.80	314.67

Table S12. HI prevalence estimates for the year 2005, per hundred thousand ($^{0}/_{0000}$) by WHO region

Age Groups	Total pop.	25dB > HI ≤ 40dB			40dB > HI ≤ 60dB			60dB > HI ≤ 80dB			80dB > HI			TOTAL (HI >25dB)		
		HI	MIN	MAX	HI	MIN	MAX	HI	MIN	MAX	HI	MIN	MAX	HI	MIN	MAX
Asia Central	76814553	59.30	53.72	64.89	20.09	19.47	20.71	5.17	5.01	5.32	1.46	1.41	1.50	86.01	79.61	92.41
Asia East	1366340458	67.38	61.79	72.96	22.82	22.20	23.43	5.39	5.25	5.54	1.28	1.24	1.31	96.87	90.49	103.25
Asia Pacific High Income	180467879	15.54	9.98	21.12	3.13	2.51	3.74	0.37	0.30	0.44	0.04	0.03	0.05	19.07	12.82	25.35
Asia South	1498562845	499.58	493.99	505.16	262.74	262.12	263.35	128.10	127.79	128.40	79.97	79.77	80.17	970.38	963.67	977.08
Asia South East	573902462	97.65	92.06	103.24	36.05	35.44	36.67	10.04	9.87	10.21	2.94	2.89	2.99	146.69	140.27	153.11
Australasia	24407320	11.59	6.08	17.18	1.87	1.27	2.48	0.16	0.10	0.21	0.01	0.02	0.01	13.64	7.46	19.88
Caribbean	38179725	50.49	44.91	56.08	16.87	16.26	17.49	4.51	4.35	4.66	1.34	1.30	1.38	73.21	66.81	79.61
Europe Central	118749415	38.71	33.13	44.30	9.67	9.06	10.29	1.54	1.44	1.64	0.24	0.23	0.26	50.17	43.86	56.48
Europe Eastern	211613757	41.22	35.64	46.81	10.05	9.43	10.66	1.53	1.44	1.62	0.23	0.22	0.25	53.03	46.73	59.34
Europe Western	407712685	11.34	5.84	16.93	1.85	1.24	2.47	0.17	0.16	0.19	0.01	0.01	0.01	13.38	7.24	19.60
Latin America Andean	49517274	27.73	22.14	33.32	15.28	14.66	15.90	6.90	6.63	7.18	3.12	3.00	3.24	53.04	46.43	59.64
Latin America Central	215171064	39.71	34.13	45.30	10.76	10.14	11.37	1.95	1.84	2.07	0.36	0.34	0.38	52.79	46.45	59.12
Latin America Southern	58370932	39.38	33.79	44.97	10.18	9.57	10.80	1.69	1.59	1.79	0.28	0.26	0.30	51.54	45.21	57.86
Latin America Tropical	192735096	38.00	32.42	43.59	9.67	9.06	10.29	1.56	1.46	1.66	0.25	0.24	0.27	49.49	43.17	55.80
North Africa Middle East	410799625	58.69	53.11	64.28	20.89	20.27	21.50	5.35	5.19	5.50	1.39	1.35	1.43	86.32	79.92	92.71
North America High Income	332116959	11.58	6.06	17.16	2.26	1.64	2.87	0.26	0.19	0.33	0.03	0.02	0.04	14.12	7.92	20.40
Oceania	9003928	305.12	299.50	310.74	140.02	139.42	140.62	49.58	49.36	49.80	17.54	17.45	17.63	512.25	505.72	518.78
Sub-Saharan Africa Central	84411831	225.90	220.31	231.49	61.31	60.70	61.93	11.93	11.81	12.05	2.87	2.84	2.89	302.01	295.66	308.36
Sub-Saharan Africa East	314207088	195.09	189.50	200.67	83.17	82.55	83.78	29.39	29.18	29.61	11.42	11.34	11.51	319.07	312.56	325.57
Sub-Saharan Africa Southern	68019317	67.68	62.10	73.27	20.75	20.13	21.36	4.33	4.21	4.46	0.92	0.89	0.95	93.68	87.33	100.04
Sub-Saharan Africa West	300599228	260.84	255.25	266.42	68.45	67.84	69.07	12.12	12.02	12.23	2.30	2.28	2.32	343.71	337.38	350.04
Total	6531703441	174.21	168.63	179.80	79.89	79.28	80.51	34.35	34.18	34.52	19.77	19.70	19.84	308.23	301.80	314.67

Table S13. Mortality estimates for the year 2005, by age group

		CASES			Prevalence $^{0}/_{000000}$		
Age Groups	Total pop.	Deaths	MIN	MAX	Deaths	MIN	MAX
0-11m.	127842788	1092	934	1088	85.42	73.06	85.10
1 - 4	497107358	4501	4298	4836	90.54	86.46	97.28
5 - 9	610073590	2371	2270	2553	38.86	37.21	41.85
10 - 14	613180667	1993	1901	2131	32.50	31.00	34.75
15 - 19	610164733	1188	1160	1292	19.47	19.01	21.17
20 - 24	560473407	769	721	832	13.72	12.86	14.84
25 - 34	1007681602	1366	1298	1483	13.56	12.88	14.72
35 - 44	888222451	1698	1601	1857	19.12	18.02	20.91
45 - 54	689115655	1021	965	1105	14.82	14.00	16.04
55 - 64	449003044	1049	994	1135	23.36	22.14	25.28
65 - 74	298397449	1470	1399	1603	49.26	46.88	53.72
75 - 84	146420903	2287	2183	2462	156.19	149.09	168.15
85 +	34019794	609	569	683	179.01	167.26	200.77
Total	6531703441	21414	20293	23060	32.78	31.07	35.30

Table S14. Mortality estimates for the year 2005, by WHO region

Age Groups	Total pop.	CASES			Prevalence $^{0}/_{000000}$		
		Deaths	MIN	MAX	Deaths	MIN	MAX
Asia Central	76814553	145	94	238	18.88	12.24	30.98
Asia East	1366340458	1274	1235	1315	9.32	9.04	9.62
Asia Pacific High Income	180467879	31	18	45	1.72	1.00	2.49
Asia South	1498562845	10322	10221	10343	68.88	68.21	69.02
Asia South East	573902462	1559	1445	1711	27.16	25.18	29.81
Australasia	24407320	9	0	24	3.69	0.00	9.83
Caribbean	38179725	71	48	301	18.60	12.57	78.84
Europe Central	118749415	59	31	191	4.97	2.61	16.08
Europe Eastern	211613757	121	100	197	5.72	4.73	9.31
Europe Western	407712685	115	103	127	2.82	2.53	3.11
Latin America Andean	49517274	63	61	63	12.72	12.32	12.72
Latin America Central	215171064	266	197	365	12.36	9.16	16.96
Latin America Southern	58370932	35	23	79	6.00	3.94	13.53
Latin America Tropical	192735096	185	170	204	9.60	8.82	10.58
North Africa Middle East	410799625	902	759	1116	21.96	18.48	27.17
North America High Income	332116959	54	41	63	1.63	1.23	1.90
Oceania	9003928	91	78	101	101.07	86.63	112.17
Sub-Saharan Africa Central	84411831	812	762	867	96.20	90.27	102.71
Sub-Saharan Africa East	314207088	2309	2146	2457	73.49	68.30	78.20
Sub-Saharan Africa Southern	68019317	231	198	297	33.96	29.11	43.66
Sub-Saharan Africa West	300599228	2760	2563	2956	91.82	85.26	98.34
Total	6531703441	21414	20293	23060	32.78	31.07	35.30

Table S15. Asia, Central: 2005 estimates of AOM and CSOM incidence, HI prevalence and mortality (cases)

Age Groups	Total pop.	AOM incidence			CSOM incidence			HI best ear: 25dB > HI ≤ 40dB			40dB > HI ≤ 60dB			60dB > HI ≤ 80dB			80dB > HI			Deaths		
		AOM	MIN	MAX	CSOM	MIN	MAX	HI	MIN	MAX	HI	MIN	MAX	HI	MIN	MAX	HI	MIN	MAX	Deaths	MIN	MAX
0-11m.	1555670	507476	496796	518156	14801	14003	15599	191	104	278	59	49	69	15	13	17	3	3	3	7	4	10
1 - 4	5511222	2330625	2292790	2368460	35254	32426	38082	1697	1389	2005	536	502	570	123	115	131	33	31	35	27	17	45
5 - 9	7085171	1180208	1131568	1228848	38328	34692	41964	2624	2228	3020	835	791	879	197	187	207	51	48	54	15	13	26
10 - 14	8414691	1037513	979746	1095280	31633	27315	35951	3393	2923	3863	1097	1045	1149	265	252	278	70	67	73	13	6	21
15 - 19	8650353	240862	181477	300247	25940	21501	30379	3787	3304	4270	1240	1187	1293	303	290	316	80	77	83	6	7	12
20 - 24	7333688	187771	137425	238117	36589	32826	40352	3677	3267	4087	1215	1170	1260	299	288	310	81	78	84	6	2	7
25 - 34	11746893	156634	75991	237277	37966	31938	43994	6639	5983	7295	2203	2131	2275	546	528	564	148	143	153	10	4	15
35 - 44	10208948	138968	68883	209053	31225	25987	36463	7105	6535	7675	2397	2334	2460	610	594	626	169	165	173	10	8	18
45 - 54	7779175	126061	72657	179465	34255	30263	38247	6240	5806	6674	2165	2117	2213	574	561	587	166	162	170	8	5	14
55 - 64	3562837	61776	37317	86235	13611	11783	15439	3477	3278	3676	1234	1212	1256	338	332	344	101	99	103	7	4	13
65 - 74	3253272	63565	41231	85899	7353	5684	9022	4066	3884	4248	1476	1456	1496	417	411	423	130	128	132	11	7	14
75 - 84	1462852	32070	22027	42113	3320	2569	4071	2228	2146	2310	821	812	830	237	234	240	74	73	75	20	14	35
85 +	249781	5276	3561	6991	572	444	700	428	414	442	155	153	157	44	44	44	12	12	12	5	3	8
Total	76814553	6068805	5541469	6596141	310847	271431	350263	45552	41261	49843	15433	14959	15907	3968	3849	4087	1118	1086	1150	145	94	238

Table S16. Asia, Central: 2005 estimates of AOM and CSOM incidence, HI prevalence and mortality (proportions)

Age Groups	Total pop.	AOM% incidence			CSOM‰ incidence			HI best ear °/°°°°												Deaths°/°°°°°°		
								25dB > HI ≤ 40dB			40dB > HI ≤ 60dB			60dB > HI ≤ 80dB			80dB > HI					
		AOM	MIN	MAX	CSOM	MIN	MAX	HI	MIN	MAX	HI	MIN	MAX	HI	MIN	MAX	HI	MIN	MAX	Deaths	MIN	MAX
0-11m.	1555670	32.62	31.93	33.31	9.51	9.00	10.03	12.28	6.69	17.87	3.79	3.15	4.44	0.96	0.84	1.09	0.19	0.19	0.19	45.00	25.71	64.28
1 - 4	5511222	42.29	41.60	42.98	6.40	5.88	6.91	30.79	25.20	36.38	9.73	9.11	10.34	2.23	2.09	2.38	0.60	0.56	0.64	48.99	30.85	81.65
5 - 9	7085171	16.66	15.97	17.34	5.41	4.90	5.92	37.04	31.45	42.62	11.79	11.16	12.41	2.78	2.64	2.92	0.72	0.68	0.76	21.17	18.35	36.70
10 - 14	8414691	12.33	11.64	13.02	3.76	3.25	4.27	40.32	34.74	45.91	13.04	12.42	13.65	3.15	2.99	3.30	0.83	0.80	0.87	15.45	7.13	24.96
15 - 19	8650353	2.78	2.10	3.47	3.00	2.49	3.51	43.78	38.19	49.36	14.33	13.72	14.95	3.50	3.35	3.65	0.92	0.89	0.96	6.94	8.09	13.87
20 - 24	7333688	2.56	1.87	3.25	4.99	4.48	5.50	50.14	44.55	55.73	16.57	15.95	17.18	4.08	3.93	4.23	1.10	1.06	1.15	8.18	2.73	9.54
25 - 34	11746893	1.33	0.65	2.02	3.23	2.72	3.75	56.52	50.93	62.10	18.75	18.14	19.37	4.65	4.49	4.80	1.26	1.22	1.30	8.51	3.41	12.77
35 - 44	10208948	1.36	0.67	2.05	3.06	2.55	3.57	69.60	64.01	75.18	23.48	22.86	24.10	5.98	5.82	6.13	1.66	1.62	1.69	9.80	7.84	17.63
45 - 54	7779175	1.62	0.93	2.31	4.40	3.89	4.92	80.21	74.64	85.79	27.83	27.21	28.45	7.38	7.21	7.55	2.13	2.08	2.19	10.28	6.43	18.00
55 - 64	3562837	1.73	1.05	2.42	3.82	3.31	4.33	97.59	92.01	103.18	34.64	34.02	35.25	9.49	9.32	9.66	2.83	2.78	2.89	19.65	11.23	36.49
65 - 74	3253272	1.95	1.27	2.64	2.26	1.75	2.77	124.98	119.39	130.58	45.37	44.75	45.98	12.82	12.63	13.00	4.00	3.93	4.06	33.81	21.52	43.03
75 - 84	1462852	2.19	1.51	2.88	2.27	1.76	2.78	152.31	146.70	157.91	56.12	55.51	56.74	16.20	16.00	16.41	5.06	4.99	5.13	136.72	95.70	239.26
85 +	249781	2.11	1.43	2.80	2.29	1.78	2.80	171.35	165.75	176.96	62.05	61.25	62.86	17.62	17.62	17.62	4.80	4.80	4.80	200.18	120.11	320.28
Total	76814553	7.90	7.21	8.59	4.05	3.53	4.56	59.30	53.72	64.89	20.09	19.47	20.71	5.17	5.01	5.32	1.46	1.41	1.50	18.88	12.24	30.98

Table S17. Asia, East: 2005 estimates of AOM and CSOM incidence, HI prevalence and mortality (cases)

Age Groups	Total pop.	AOM incidence			CSOM incidence			HI best ear: 25dB > HI ≤ 40dB			HI best ear: 40dB > HI ≤ 60dB			HI best ear: 60dB > HI ≤ 80dB			HI best ear: 80dB > HI			Deaths		
		AOM	MIN	MAX	CSOM	MIN	MAX	HI	MIN	MAX	HI	MIN	MAX	HI	MIN	MAX	HI	MIN	MAX	Deaths	MIN	MAX
0-11m.	16354761	3881584	3769308	3993860	121016	112624	129408	2169	1256	3082	734	633	835	174	150	198	41	35	47	46	43	45
1 - 4	72131762	19759747	19264560	20254934	356447	319434	393460	23901	19872	27930	8094	7650	8538	1912	1807	2017	452	427	477	221	215	228
5 - 9	99365517	11287251	10605104	11969398	412664	361677	463651	39499	33949	45049	13372	12761	13983	3159	3015	3303	747	713	781	135	129	139
10 - 14	107081684	5647536	4912417	6382655	390984	336038	445930	46110	40129	52091	15612	14953	16271	3688	3532	3844	873	836	910	71	70	75
15 - 19	120881804	2146848	1316991	2976705	339873	277846	401900	56071	49320	62822	19000	18256	19744	4492	4316	4668	1063	1021	1105	39	39	41
20 - 24	104634949	1803847	1085525	2522169	523011	469320	576702	55374	49530	61218	18748	18104	19392	4429	4277	4581	1047	1011	1083	41	39	42
25 - 34	224300518	2117449	577619	3657279	705936	590842	821030	133577	121050	146104	45244	43864	46624	10692	10366	11018	2529	2452	2606	71	69	73
35 - 44	237101384	2255291	627583	3882999	699310	577648	820972	172589	159347	185831	58464	57005	59923	13819	13474	14164	3269	3187	3351	107	104	111
45 - 54	171555611	1938341	760607	3116075	791586	703557	879615	141668	132087	151249	47990	46935	49045	11344	11095	11593	2684	2625	2743	90	87	92
55 - 64	107462330	1309559	571827	2047291	430139	374997	485281	106707	100705	112709	36121	35460	36782	8530	8374	8686	2018	1981	2055	92	89	95
65 - 74	69488336	940176	463136	1417216	159156	123500	194812	87321	83440	91202	29553	29126	29980	6979	6878	7080	1650	1626	1674	123	120	128
75 - 84	30338697	465104	256828	673380	69838	54270	85406	46006	44312	47700	15576	15389	15763	3680	3636	3724	870	860	880	185	179	191
85 +	5643105	84101	45361	122841	13159	10263	16055	9647	9332	9962	3268	3233	3303	773	765	781	183	181	185	53	52	55
Total	1366340458	53636834	44256866	63016802	5013119	4312016	5714222	920639	844329	996949	311776	303369	320183	73671	71685	75657	17426	16955	17897	1274	1235	1315

Table S18. Asia, East: 2005 estimates of AOM and CSOM incidence, HI prevalence and mortality (proportions)

								HI best ear °/°°°°														
		AOM% incidence			CSOM‰ incidence			25dB > HI ≤ 40dB			40dB > HI ≤ 60dB			60dB > HI ≤ 80dB			80dB > HI			Deaths°/°°°°°°		
Age Groups	Total pop.	AOM	MIN	MAX	CSOM	MIN	MAX	HI	MIN	MAX	HI	MIN	MAX	HI	MIN	MAX	HI	MIN	MAX	Deaths	MIN	MAX
0-11m.	16354761	23.73	23.05	24.42	7.40	6.89	7.91	13.26	7.68	18.84	4.49	3.87	5.11	1.06	0.92	1.21	0.25	0.21	0.29	28.13	26.29	27.51
1 - 4	72131762	27.39	26.71	28.08	4.94	4.43	5.45	33.14	27.55	38.72	11.22	10.61	11.84	2.65	2.51	2.80	0.63	0.59	0.66	30.64	29.81	31.61
5 - 9	99365517	11.36	10.67	12.05	4.15	3.64	4.67	39.75	34.17	45.34	13.46	12.84	14.07	3.18	3.03	3.32	0.75	0.72	0.79	13.59	12.98	13.99
10 - 14	107081684	5.27	4.59	5.96	3.65	3.14	4.16	43.06	37.48	48.65	14.58	13.96	15.19	3.44	3.30	3.59	0.82	0.78	0.85	6.63	6.54	7.00
15 - 19	120881804	1.78	1.09	2.46	2.81	2.30	3.32	46.38	40.80	51.97	15.72	15.10	16.33	3.72	3.57	3.86	0.88	0.84	0.91	3.23	3.23	3.39
20 - 24	104634949	1.72	1.04	2.41	5.00	4.49	5.51	52.92	47.34	58.51	17.92	17.30	18.53	4.23	4.09	4.38	1.00	0.97	1.04	3.92	3.73	4.01
25 - 34	224300518	0.94	0.26	1.63	3.15	2.63	3.66	59.55	53.97	65.14	20.17	19.56	20.79	4.77	4.62	4.91	1.13	1.09	1.16	3.17	3.08	3.25
35 - 44	237101384	0.95	0.26	1.64	2.95	2.44	3.46	72.79	67.21	78.38	24.66	24.04	25.27	5.83	5.68	5.97	1.38	1.34	1.41	4.51	4.39	4.68
45 - 54	171555611	1.13	0.44	1.82	4.61	4.10	5.13	82.58	76.99	88.16	27.97	27.36	28.59	6.61	6.47	6.76	1.56	1.53	1.60	5.25	5.07	5.36
55 - 64	107462330	1.22	0.53	1.91	4.00	3.49	4.52	99.30	93.71	104.88	33.61	33.00	34.23	7.94	7.79	8.08	1.88	1.84	1.91	8.56	8.28	8.84
65 - 74	69488336	1.35	0.67	2.04	2.29	1.78	2.80	125.66	120.08	131.25	42.53	41.91	43.14	10.04	9.90	10.19	2.37	2.34	2.41	17.70	17.27	18.42
75 - 84	30338697	1.53	0.85	2.22	2.30	1.79	2.82	151.64	146.06	157.22	51.34	50.72	51.96	12.13	11.98	12.27	2.87	2.83	2.90	60.98	59.00	62.96
85 +	5643105	1.49	0.80	2.18	2.33	1.82	2.85	170.95	165.37	176.53	57.91	57.29	58.53	13.70	13.56	13.84	3.24	3.21	3.28	93.92	92.15	97.46
Total	1366340458	3.93	3.24	4.61	3.67	3.16	4.18	67.38	61.79	72.96	22.82	22.20	23.43	5.39	5.25	5.54	1.28	1.24	1.31	9.32	9.04	9.62

Table S19. Asia Pacific, High Income: 2005 estimates of AOM and CSOM incidence, HI prevalence and mortality (cases)

Age Groups	Total pop.	AOM incidence			CSOM incidence			HI best ear												Deaths		
								25dB > HI ≤ 40dB			40dB > HI ≤ 60dB			60dB > HI ≤ 80dB			80dB > HI					
		AOM	MIN	MAX	CSOM	MIN	MAX	HI	MIN	MAX	HI	MIN	MAX	HI	MIN	MAX	HI	MIN	MAX	Deaths	MIN	MAX
0-11m.	1539688	330457	323697	337217	2447	1134	3760	40	0	126	7	0	16	1	0	2	0	0	0	1	0	1
1 - 4	6860549	1660968	1630845	1691091	7267	1415	13119	448	65	831	90	48	132	11	6	16	1	1	1	3	2	5
5 - 9	9446890	935140	893661	976619	8398	340	16456	740	212	1268	149	91	207	18	11	25	2	1	3	2	1	3
10 - 14	9789971	280718	237733	323703	9528	1178	17878	829	282	1376	168	108	228	20	13	27	2	1	3	1	1	1
15 - 19	10109743	215428	171039	259817	25898	17275	34521	924	359	1489	188	126	250	22	15	29	3	2	4	0	0	0
20 - 24	11572049	246173	195364	296982	19399	9529	29269	1210	564	1856	246	175	317	29	21	37	3	2	4	2	1	2
25 - 34	27231191	518719	399155	638283	52874	29648	76100	3206	1685	4727	648	480	816	77	57	97	9	7	11	1	1	1
35 - 44	25978925	499349	385283	613415	71765	49607	93923	3731	2280	5182	756	596	916	91	72	110	10	8	12	5	3	7
45 - 54	24188722	565829	459623	672035	96358	75727	116989	3949	2598	5300	798	649	947	95	77	113	11	9	13	2	1	3
55 - 64	23596068	602292	498689	705895	120678	100552	140804	4638	3320	5956	929	784	1074	109	92	126	13	11	15	4	1	5
65 - 74	17221291	493128	417514	568742	74462	59773	89151	4294	3332	5256	860	754	966	101	89	113	12	11	13	2	3	5
75 - 84	9813459	322391	279303	365479	42703	34333	51073	2964	2416	3512	591	531	651	68	61	75	8	7	9	6	2	7
85 +	3119333	99395	85699	113091	13775	11114	16436	1065	891	1239	211	192	230	24	22	26	3	3	3	2	2	5
Total	180467879	6769987	5977605	7562369	545552	391625	699479	28038	18004	38118	5641	4534	6750	666	536	796	77	63	91	31	18	45

Table S20. Asia Pacific, High Income: 2005 estimates of AOM and CSOM incidence, HI prevalence and mortality (proportions)

Age Groups	Total pop.	AOM% incidence			CSOM‰ incidence			HI best ear °/°°°° 25dB > HI ≤ 40dB			40dB > HI ≤ 60dB			60dB > HI ≤ 80dB			80dB > HI			Deaths°/°°°°°°		
		AOM	MIN	MAX	CSOM	MIN	MAX	HI	MIN	MAX	HI	MIN	MAX	HI	MIN	MAX	HI	MIN	MAX	Deaths	MIN	MAX
0-11m.	1539688	21.46	21.02	21.90	1.59	0.74	2.44	2.60	0.00	8.18	0.45	0.00	1.04	0.06	0.00	0.13	0.00	0.00	0.00	6.49	0.00	6.49
1 - 4	6860549	24.21	23.77	24.65	1.06	0.21	1.91	6.53	0.95	12.11	1.31	0.70	1.92	0.16	0.09	0.23	0.01	0.01	0.01	4.37	2.92	7.29
5 - 9	9446890	9.90	9.46	10.34	0.89	0.04	1.74	7.83	2.24	13.42	1.58	0.96	2.19	0.19	0.12	0.26	0.02	0.01	0.03	2.12	1.06	3.18
10 - 14	9789971	2.87	2.43	3.31	0.97	0.12	1.83	8.47	2.88	14.06	1.72	1.10	2.33	0.20	0.13	0.28	0.02	0.01	0.03	1.02	1.02	1.02
15 - 19	10109743	2.13	1.69	2.57	2.56	1.71	3.41	9.14	3.55	14.73	1.86	1.25	2.47	0.22	0.15	0.29	0.03	0.02	0.04	0.00	0.00	0.00
20 - 24	11572049	2.13	1.69	2.57	1.68	0.82	2.53	10.46	4.87	16.04	2.13	1.51	2.74	0.25	0.18	0.32	0.03	0.02	0.03	1.73	0.86	1.73
25 - 34	27231191	1.90	1.47	2.34	1.94	1.09	2.79	11.77	6.19	17.36	2.38	1.76	3.00	0.28	0.21	0.36	0.03	0.03	0.04	0.37	0.37	0.37
35 - 44	25978925	1.92	1.48	2.36	2.76	1.91	3.62	14.36	8.78	19.95	2.91	2.29	3.53	0.35	0.28	0.42	0.04	0.03	0.05	1.92	1.15	2.69
45 - 54	24188722	2.34	1.90	2.78	3.98	3.13	4.84	16.33	10.74	21.91	3.30	2.68	3.92	0.39	0.32	0.47	0.05	0.04	0.05	0.83	0.41	1.24
55 - 64	23596068	2.55	2.11	2.99	5.11	4.26	5.97	19.66	14.07	25.24	3.94	3.32	4.55	0.46	0.39	0.53	0.06	0.05	0.06	1.70	0.42	2.12
65 - 74	17221291	2.86	2.42	3.30	4.32	3.47	5.18	24.93	19.35	30.52	4.99	4.38	5.61	0.59	0.52	0.66	0.07	0.06	0.08	1.16	1.74	2.90
75 - 84	9813459	3.29	2.85	3.72	4.35	3.50	5.20	30.20	24.62	35.79	6.02	5.41	6.63	0.69	0.62	0.76	0.08	0.07	0.09	6.11	2.04	7.13
85 +	3119333	3.19	2.75	3.63	4.42	3.56	5.27	34.14	28.56	39.72	6.76	6.16	7.37	0.77	0.71	0.83	0.10	0.10	0.10	6.41	6.41	16.03
Total	180467879	3.75	3.31	4.19	3.02	2.17	3.88	15.54	9.98	21.12	3.13	2.51	3.74	0.37	0.30	0.44	0.04	0.03	0.05	1.72	1.00	2.49

Table S21. Asia, South: 2005 estimates of AOM and CSOM incidence, HI prevalence and mortality (cases)

Age Groups	Total pop.	AOM incidence			CSOM incidence			HI best ear												Deaths		
								25dB > HI ≤ 40dB			40dB > HI ≤ 60dB			60dB > HI ≤ 80dB			80dB > HI					
		AOM	MIN	MAX	CSOM	MIN	MAX	HI	MIN	MAX	HI	MIN	MAX	HI	MIN	MAX	HI	MIN	MAX	Deaths	MIN	MAX
0-11m.	35291481	16996795	16754518	17239072	855198	837089	873307	41090	39119	43061	22991	22774	23208	12957	12835	13079	9651	9560	9742	459	412	418
1 - 4	137735303	92438494	91492937	93384051	2223276	2152601	2293951	397358	389665	405051	220673	219826	221520	122209	121740	122678	89278	88935	89621	2191	2178	2204
5 - 9	169390240	42272227	41109358	43435096	2292116	2205198	2379034	577983	568523	587443	316211	315169	317253	169258	168700	169816	118949	118557	119341	1164	1159	1173
10 - 14	166930919	38569455	37423469	39715441	752803	667147	838459	609480	600157	618803	328520	327493	329547	169960	169429	170491	114663	114305	115021	1015	1008	1019
15 - 19	155568539	6735567	5667584	7803550	659791	579965	739617	606073	597384	614762	323569	322612	324526	163478	162994	163962	106930	106614	107246	565	563	571
20 - 24	139810927	5390656	4430850	6350462	702593	630853	774333	623006	615198	630814	329545	328685	330405	163135	162709	163561	104030	103758	104302	355	352	356
25 - 34	230585823	4472943	2889964	6055922	891915	773596	1010234	1157937	1145059	1170815	606944	605526	608362	293959	293272	294646	182042	181617	182467	624	622	628
35 - 44	179006010	3488362	2259480	4717244	681531	589679	773383	1093180	1083183	1103177	567169	566068	568270	267514	266995	268033	159322	159013	159631	776	771	782
45 - 54	134114824	3147964	2227262	4068666	417394	348576	486212	928340	920850	935830	478002	477177	478827	220291	219911	220671	126277	126059	126495	491	488	494
55 - 64	80527111	2054944	1502123	2607765	212625	171305	253945	670495	665998	674992	346240	345745	346735	160851	160621	161081	93421	93287	93555	501	498	502
65 - 74	48059063	1366262	1036335	1696189	101343	76683	126003	504459	501775	507143	257748	257452	258044	115832	115699	115965	63485	63412	63558	730	726	735
75 - 84	18266867	590457	465054	715860	38573	29200	47946	230464	229444	231484	116469	116357	116581	50549	50500	50598	25919	25894	25944	1156	1149	1163
85 +	3275738	102008	79520	124496	6944	5263	8625	46588	46405	46771	23225	23205	23245	9595	9587	9603	4410	4406	4414	295	295	298
Total	1498562845	217626134	207338454	227913814	9836102	9067155	10605049	7486453	7402760	7570146	3937306	3928089	3946523	1919588	1914992	1924184	1198377	1195417	1201337	10322	10221	10343

Table S22. Asia, South: 2005 estimates of AOM and CSOM incidence, HI prevalence and mortality (proportions)

Age Groups	Total pop.	AOM% incidence			CSOM‰ incidence			HI best ear °/°°°°												Deaths°/°°°°°°		
								25dB > HI ≤ 40dB			40dB > HI ≤ 60dB			60dB > HI ≤ 80dB			80dB > HI					
		AOM	MIN	MAX	CSOM	MIN	MAX	HI	MIN	MAX	HI	MIN	MAX	HI	MIN	MAX	HI	MIN	MAX	Deaths	MIN	MAX
0-11m.	35291481	48.16	47.47	48.85	24.23	23.72	24.75	116.43	110.85	122.02	65.15	64.53	65.76	36.71	36.37	37.06	27.35	27.09	27.60	130.06	116.74	118.44
1 - 4	137735303	67.11	66.43	67.80	16.14	15.63	16.65	288.49	282.91	294.08	160.22	159.60	160.83	88.73	88.39	89.07	64.82	64.57	65.07	159.07	158.13	160.02
5 - 9	169390240	24.96	24.27	25.64	13.53	13.02	14.04	341.21	335.63	346.80	186.68	186.06	187.29	99.92	99.59	100.25	70.22	69.99	70.45	68.72	68.42	69.25
10 - 14	166930919	23.11	22.42	23.79	4.51	4.00	5.02	365.11	359.52	370.69	196.80	196.18	197.42	101.81	101.50	102.13	68.69	68.47	68.90	60.80	60.38	61.04
15 - 19	155568539	4.33	3.64	5.02	4.24	3.73	4.75	389.59	384.00	395.17	207.99	207.38	208.61	105.08	104.77	105.40	68.73	68.53	68.94	36.32	36.19	36.70
20 - 24	139810927	3.86	3.17	4.54	5.03	4.51	5.54	445.61	440.02	451.19	235.71	235.09	236.32	116.68	116.38	116.99	74.41	74.21	74.60	25.39	25.18	25.46
25 - 34	230585823	1.94	1.25	2.63	3.87	3.35	4.38	502.17	496.59	507.76	263.22	262.60	263.83	127.48	127.19	127.78	78.95	78.76	79.13	27.06	26.97	27.23
35 - 44	179006010	1.95	1.26	2.64	3.81	3.29	4.32	610.69	605.11	616.28	316.84	316.23	317.46	149.44	149.15	149.73	89.00	88.83	89.18	43.35	43.07	43.69
45 - 54	134114824	2.35	1.66	3.03	3.11	2.60	3.63	692.20	686.61	697.78	356.41	355.80	357.03	164.26	163.97	164.54	94.16	93.99	94.32	36.61	36.39	36.83
55 - 64	80527111	2.55	1.87	3.24	2.64	2.13	3.15	832.63	827.05	838.22	429.97	429.35	430.58	199.75	199.46	200.03	116.01	115.85	116.18	62.22	61.84	62.34
65 - 74	48059063	2.84	2.16	3.53	2.11	1.60	2.62	1049.66	1044.08	1055.25	536.32	535.70	536.93	241.02	240.74	241.30	132.10	131.95	132.25	151.90	151.06	152.94
75 - 84	18266867	3.23	2.55	3.92	2.11	1.60	2.62	1261.65	1256.07	1267.23	637.60	636.98	638.21	276.73	276.46	276.99	141.89	141.75	142.03	632.84	629.01	636.67
85 +	3275738	3.11	2.43	3.80	2.12	1.61	2.63	1422.21	1416.63	1427.80	709.00	708.39	709.61	292.91	292.67	293.16	134.63	134.50	134.75	900.56	900.56	909.72
Total	1498562845	14.52	13.84	15.21	6.56	6.05	7.08	499.58	493.99	505.16	262.74	262.12	263.35	128.10	127.79	128.40	79.97	79.77	80.17	68.88	68.21	69.02

Table S23. Asia, Southeast: 2005 estimates of AOM and CSOM incidence, HI prevalence and mortality (cases)

Age Groups	Total pop.	AOM incidence			CSOM incidence			HI best ear												Deaths		
								25dB > HI ≤ 40dB			40dB > HI ≤ 60dB			60dB > HI ≤ 80dB			80dB > HI					
		AOM	MIN	MAX	CSOM	MIN	MAX	HI	MIN	MAX	HI	MIN	MAX	HI	MIN	MAX	HI	MIN	MAX	Deaths	MIN	MAX
0-11m.	11761578	3885935	3805191	3966679	162724	156689	168759	2426	1769	3083	864	792	936	230	211	249	65	60	70	68	60	70
1 - 4	44677924	19111277	18804562	19417992	412954	390029	435879	23303	20808	25798	8312	8037	8587	2225	2151	2299	628	607	649	317	291	345
5 - 9	54816595	9188533	8812215	9564851	427285	399157	455413	34656	31594	37718	12383	12046	12720	3324	3233	3415	937	911	963	165	153	184
10 - 14	56564524	7086229	6697912	7474546	225311	196286	254336	38790	35631	41949	13896	13548	14244	3734	3640	3828	1057	1031	1083	131	122	143
15 - 19	56249300	1590952	1204799	1977105	189653	160790	218516	41726	38584	44868	15081	14735	15427	4091	3997	4185	1164	1137	1191	66	63	73
20 - 24	54013759	1402776	1031970	1773582	270064	242348	297780	45739	42722	48756	16675	16343	17007	4563	4472	4654	1307	1281	1333	53	50	60
25 - 34	95041067	1291349	638889	1943809	325032	276264	373800	90768	85460	96076	33402	32817	33987	9225	9064	9386	2669	2622	2716	96	89	106
35 - 44	78531881	1068817	529693	1607941	256608	216311	296905	90173	85787	94559	33573	33090	34056	9429	9293	9565	2783	2743	2823	128	120	140
45 - 54	57448931	939211	544822	1333600	232420	202942	261898	74719	71510	77928	28114	27761	28467	8030	7929	8131	2419	2389	2449	86	79	94
55 - 64	33643301	589237	358275	820199	117806	100543	135069	51865	49986	53744	19559	19352	19766	5604	5545	5663	1691	1673	1709	90	83	97
65 - 74	21151973	414578	269369	559787	46938	36084	57792	41454	40273	42635	15614	15484	15744	4453	4416	4490	1336	1325	1347	119	111	134
75 - 84	8493824	193457	135147	251767	18884	14526	23242	20645	20171	21119	7831	7779	7883	2253	2238	2268	680	675	685	188	178	206
85 +	1507805	33906	23555	44257	3381	2607	4155	4152	4068	4236	1611	1602	1620	479	476	482	150	149	151	52	46	59
Total	573902462	46796257	42856399	50736115	2689060	2394576	2983544	560416	528363	592469	206915	203386	210444	57640	56665	58615	16886	16603	17169	1559	1445	1711

Table S24. Asia, Southeast: 2005 estimates of AOM and CSOM incidence, HI prevalence and mortality (proportions)

Age Groups	Total pop.	AOM% incidence			CSOM‰ incidence			HI best ear °/°°°°												Deaths°/°°°°°°		
								25dB > HI ≤ 40dB			40dB > HI ≤ 60dB			60dB > HI ≤ 80dB			80dB > HI					
		AOM	MIN	MAX	CSOM	MIN	MAX	HI	MIN	MAX	HI	MIN	MAX	HI	MIN	MAX	HI	MIN	MAX	Deaths	MIN	MAX
0-11m.	11761578	33.04	32.35	33.73	13.84	13.32	14.35	20.63	15.04	26.21	7.35	6.73	7.96	1.96	1.79	2.12	0.55	0.51	0.60	57.82	51.01	59.52
1 - 4	44677924	42.78	42.09	43.46	9.24	8.73	9.76	52.16	46.57	57.74	18.60	17.99	19.22	4.98	4.81	5.15	1.41	1.36	1.45	70.95	65.13	77.22
5 - 9	54816595	16.76	16.08	17.45	7.79	7.28	8.31	63.22	57.64	68.81	22.59	21.98	23.20	6.06	5.90	6.23	1.71	1.66	1.76	30.10	27.91	33.57
10 - 14	56564524	12.53	11.84	13.21	3.98	3.47	4.50	68.58	62.99	74.16	24.57	23.95	25.18	6.60	6.44	6.77	1.87	1.82	1.91	23.16	21.57	25.28
15 - 19	56249300	2.83	2.14	3.51	3.37	2.86	3.88	74.18	68.59	79.77	26.81	26.20	27.43	7.27	7.11	7.44	2.07	2.02	2.12	11.73	11.20	12.98
20 - 24	54013759	2.60	1.91	3.28	5.00	4.49	5.51	84.68	79.09	90.27	30.87	30.26	31.49	8.45	8.28	8.62	2.42	2.37	2.47	9.81	9.26	11.11
25 - 34	95041067	1.36	0.67	2.05	3.42	2.91	3.93	95.50	89.92	101.09	35.14	34.53	35.76	9.71	9.54	9.88	2.81	2.76	2.86	10.10	9.36	11.15
35 - 44	78531881	1.36	0.67	2.05	3.27	2.75	3.78	114.82	109.24	120.41	42.75	42.14	43.37	12.01	11.83	12.18	3.54	3.49	3.59	16.30	15.28	17.83
45 - 54	57448931	1.63	0.95	2.32	4.05	3.53	4.56	130.06	124.48	135.65	48.94	48.32	49.55	13.98	13.80	14.15	4.21	4.16	4.26	14.97	13.75	16.36
55 - 64	33643301	1.75	1.06	2.44	3.50	2.99	4.01	154.16	148.58	159.75	58.14	57.52	58.75	16.66	16.48	16.83	5.03	4.97	5.08	26.75	24.67	28.83
65 - 74	21151973	1.96	1.27	2.65	2.22	1.71	2.73	195.98	190.40	201.57	73.82	73.20	74.43	21.05	20.88	21.23	6.32	6.26	6.37	56.26	52.48	63.35
75 - 84	8493824	2.28	1.59	2.96	2.22	1.71	2.74	243.06	237.48	248.64	92.20	91.58	92.81	26.53	26.35	26.70	8.01	7.95	8.06	221.34	209.56	242.53
85 +	1507805	2.25	1.56	2.94	2.24	1.73	2.76	275.37	269.80	280.94	106.84	106.25	107.44	31.77	31.57	31.97	9.95	9.88	10.01	344.87	305.08	391.30
Total	573902462	8.15	7.47	8.84	4.69	4.17	5.20	97.65	92.06	103.24	36.05	35.44	36.67	10.04	9.87	10.21	2.94	2.89	2.99	27.16	25.18	29.81

Table S25. Australasia: 2005 estimates of AOM and CSOM incidence, HI prevalence and mortality (cases)

Age Groups	Total pop.	AOM incidence			CSOM incidence			HI best ear												Deaths		
								25dB > HI ≤ 40dB			40dB > HI ≤ 60dB			60dB > HI ≤ 80dB			80dB > HI					
		AOM	MIN	MAX	CSOM	MIN	MAX	HI	MIN	MAX	HI	MIN	MAX	HI	MIN	MAX	HI	MIN	MAX	Deaths	MIN	MAX
0-11m.	313912	120991	119613	122369	1570	1302	1838	6	0	24	1	0	3	0	0	0	0	0	0	0	0	1
1 - 4	1232153	636102	630692	641512	4118	3067	5169	65	0	134	11	3	19	1	0	2	0	0	0	2	0	4
5 - 9	1594578	314938	307937	321939	4477	3117	5837	99	10	188	16	6	26	1	0	2	0	0	0	1	0	2
10 - 14	1703703	279645	272165	287125	5692	4239	7145	115	20	210	19	9	29	1	0	2	0	0	0	1	0	2
15 - 19	1704687	57651	50166	65136	4458	3004	5912	124	29	219	20	10	30	1	0	2	0	0	0	0	0	1
20 - 24	1690951	51960	44536	59384	8034	6592	9476	141	47	235	22	12	32	2	1	3	0	0	0	0	0	1
25 - 34	3429314	54152	39095	69209	10188	7263	13113	322	130	514	52	31	73	5	3	7	0	1	0	0	0	1
35 - 44	3621205	57668	41768	73568	10220	7131	13309	415	213	617	67	45	89	6	4	8	1	1	1	1	0	2
45 - 54	3350769	64232	49520	78944	15826	12968	18684	436	249	623	70	49	91	6	4	8	1	1	1	0	0	1
55 - 64	2606372	54189	42745	65633	10978	8755	13201	407	261	553	66	50	82	6	5	7	1	1	1	1	0	1
65 - 74	1661026	38578	31285	45871	4032	2615	5449	329	236	422	53	43	63	5	4	6	0	1	0	1	0	2
75 - 84	1132137	29967	24996	34938	2756	1790	3722	272	209	335	44	37	51	3	3	3	0	0	0	1	0	3
85 +	366513	9404	7795	11013	905	592	1218	99	79	119	16	14	18	1	1	1	0	0	0	1	0	3
Total	24407320	1769477	1662313	1876641	83254	62435	104073	2830	1483	4193	457	309	606	38	25	51	3	5	3	9	0	24

Table S26. Australasia: 2005 estimates of AOM and CSOM incidence, HI prevalence and mortality (proportions)

Age Groups	Total pop.	AOM% incidence			CSOM‰ incidence			HI best ear °/°°°°												Deaths°/°°°°°°		
								25dB > HI ≤ 40dB			40dB > HI ≤ 60dB			60dB > HI ≤ 80dB			80dB > HI					
		AOM	MIN	MAX	CSOM	MIN	MAX	HI	MIN	MAX	HI	MIN	MAX	HI	MIN	MAX	HI	MIN	MAX	Deaths	MIN	MAX
0-11m.	313912	38.54	38.10	38.98	5.00	4.15	5.86	1.91	0.00	7.65	0.32	0.00	0.96	0.00	0.00	0.00	0.00	0.00	0.00	0.00	0.00	31.86
1 - 4	1232153	51.63	51.19	52.06	3.34	2.49	4.20	5.28	0.00	10.88	0.89	0.24	1.54	0.08	0.00	0.16	0.00	0.00	0.00	16.23	0.00	32.46
5 - 9	1594578	19.75	19.31	20.19	2.81	1.95	3.66	6.21	0.63	11.79	1.00	0.38	1.63	0.06	0.00	0.13	0.00	0.00	0.00	6.27	0.00	12.54
10 - 14	1703703	16.41	15.97	16.85	3.34	2.49	4.19	6.75	1.17	12.33	1.12	0.53	1.70	0.06	0.00	0.12	0.00	0.00	0.00	5.87	0.00	11.74
15 - 19	1704687	3.38	2.94	3.82	2.62	1.76	3.47	7.27	1.70	12.85	1.17	0.59	1.76	0.06	0.00	0.12	0.00	0.00	0.00	0.00	0.00	5.87
20 - 24	1690951	3.07	2.63	3.51	4.75	3.90	5.60	8.34	2.78	13.90	1.30	0.71	1.89	0.12	0.06	0.18	0.00	0.00	0.00	0.00	0.00	5.91
25 - 34	3429314	1.58	1.14	2.02	2.97	2.12	3.82	9.39	3.79	14.99	1.52	0.90	2.13	0.15	0.09	0.20	0.00	0.03	0.00	0.00	0.00	2.92
35 - 44	3621205	1.59	1.15	2.03	2.82	1.97	3.68	11.46	5.88	17.04	1.85	1.24	2.46	0.17	0.11	0.22	0.03	0.03	0.03	2.76	0.00	5.52
45 - 54	3350769	1.92	1.48	2.36	4.72	3.87	5.58	13.01	7.43	18.59	2.09	1.46	2.72	0.18	0.12	0.24	0.03	0.03	0.03	0.00	0.00	2.98
55 - 64	2606372	2.08	1.64	2.52	4.21	3.36	5.06	15.62	10.01	21.22	2.53	1.92	3.15	0.23	0.19	0.27	0.04	0.04	0.04	3.84	0.00	3.84
65 - 74	1661026	2.32	1.88	2.76	2.43	1.57	3.28	19.81	14.21	25.41	3.19	2.59	3.79	0.30	0.24	0.36	0.00	0.06	0.00	6.02	0.00	12.04
75 - 84	1132137	2.65	2.21	3.09	2.43	1.58	3.29	24.03	18.46	29.59	3.89	3.27	4.50	0.26	0.26	0.26	0.00	0.00	0.00	8.83	0.00	26.50
85 +	366513	2.57	2.13	3.00	2.47	1.62	3.32	27.01	21.55	32.47	4.37	3.82	4.91	0.27	0.27	0.27	0.00	0.00	0.00	27.28	0.00	81.85
Total	24407320	7.25	6.81	7.69	3.41	2.56	4.26	11.59	6.08	17.18	1.87	1.27	2.48	0.16	0.10	0.21	0.01	0.02	0.01	3.69	0.00	9.83

Table S27. Caribbean: 2005 estimates of AOM and CSOM incidence, HI prevalence and mortality (cases)

Age Groups	Total pop.	AOM incidence			CSOM incidence			HI best ear: 25dB > HI ≤ 40dB			40dB > HI ≤ 60dB			60dB > HI ≤ 80dB			80dB > HI			Deaths		
		AOM	MIN	MAX	CSOM	MIN	MAX	HI	MIN	MAX	HI	MIN	MAX	HI	MIN	MAX	HI	MIN	MAX	Deaths	MIN	MAX
0-11m.	755604	276883	271696	282070	8115	7727	8503	77	35	119	22	17	27	4	3	5	1	1	1	2	2	11
1 - 4	3004522	1457152	1436526	1477778	21508	19966	23050	791	623	959	232	214	250	51	47	55	13	12	14	13	7	70
5 - 9	3721343	688204	662657	713751	22243	20333	24153	1176	968	1384	352	329	375	78	73	83	20	19	21	8	4	35
10 - 14	3671021	532606	507404	557808	13883	11999	15767	1253	1048	1458	380	357	403	89	84	94	24	23	25	5	2	28
15 - 19	3584041	109935	85330	134540	10968	9129	12807	1323	1123	1523	407	385	429	96	91	101	26	25	27	1	2	12
20 - 24	3254861	91847	69502	114192	16125	14455	17795	1374	1192	1556	418	398	438	99	94	104	27	26	28	4	4	15
25 - 34	5749228	80191	40722	119660	18620	15670	21570	2711	2390	3032	884	849	919	233	224	242	69	66	72	6	4	16
35 - 44	5402544	72595	35506	109684	16458	13686	19230	3108	2806	3410	1082	1049	1115	305	296	314	96	93	99	3	3	19
45 - 54	3682015	59764	34487	85041	15890	14001	17779	2415	2209	2621	837	814	860	234	228	240	70	68	72	5	3	15
55 - 64	2580729	44324	26607	62041	9683	8359	11007	2028	1884	2172	720	704	736	206	201	211	65	64	66	6	5	14
65 - 74	1654901	31610	20249	42971	3698	2849	4547	1643	1551	1735	588	578	598	169	166	172	54	53	55	8	5	26
75 - 84	852824	17857	12002	23712	1915	1477	2353	1021	973	1069	381	376	386	113	111	115	33	33	33	8	7	31
85 +	266092	5076	3249	6903	606	469	743	358	343	373	139	137	141	43	42	44	12	12	12	2	0	9
Total	38179725	3468044	3205937	3730151	159712	140120	179304	19278	17145	21411	6442	6207	6677	1720	1660	1780	510	495	525	71	48	301

Table S28. Caribbean: 2005 estimates of AOM and CSOM incidence, HI prevalence and mortality (proportions)

Age Groups	Total pop.	AOM% incidence			CSOM‰ incidence			HI best ear °/°°°° 25dB > HI ≤ 40dB			40dB > HI ≤ 60dB			60dB > HI ≤ 80dB			80dB > HI			Deaths°/°°°°°°		
		AOM	MIN	MAX	CSOM	MIN	MAX	HI	MIN	MAX	HI	MIN	MAX	HI	MIN	MAX	HI	MIN	MAX	Deaths	MIN	MAX
0-11m.	755604	36.64	35.96	37.33	10.74	10.23	11.25	10.19	4.63	15.75	2.91	2.25	3.57	0.53	0.40	0.66	0.13	0.13	0.13	26.47	26.47	145.58
1 - 4	3004522	48.50	47.81	49.19	7.16	6.65	7.67	26.33	20.74	31.92	7.72	7.12	8.32	1.70	1.56	1.83	0.43	0.40	0.47	43.27	23.30	232.98
5 - 9	3721343	18.49	17.81	19.18	5.98	5.46	6.49	31.60	26.01	37.19	9.46	8.84	10.08	2.10	1.96	2.23	0.54	0.51	0.56	21.50	10.75	94.05
10 - 14	3671021	14.51	13.82	15.19	3.78	3.27	4.29	34.13	28.55	39.72	10.35	9.72	10.98	2.42	2.29	2.56	0.65	0.63	0.68	13.62	5.45	76.27
15 - 19	3584041	3.07	2.38	3.75	3.06	2.55	3.57	36.91	31.33	42.49	11.36	10.74	11.97	2.68	2.54	2.82	0.73	0.70	0.75	2.79	5.58	33.48
20 - 24	3254861	2.82	2.14	3.51	4.95	4.44	5.47	42.21	36.62	47.81	12.84	12.23	13.46	3.04	2.89	3.20	0.83	0.80	0.86	12.29	12.29	46.08
25 - 34	5749228	1.39	0.71	2.08	3.24	2.73	3.75	47.15	41.57	52.74	15.38	14.77	15.98	4.05	3.90	4.21	1.20	1.15	1.25	10.44	6.96	27.83
35 - 44	5402544	1.34	0.66	2.03	3.05	2.53	3.56	57.53	51.94	63.12	20.03	19.42	20.64	5.65	5.48	5.81	1.78	1.72	1.83	5.55	5.55	35.17
45 - 54	3682015	1.62	0.94	2.31	4.32	3.80	4.83	65.59	59.99	71.18	22.73	22.11	23.36	6.36	6.19	6.52	1.90	1.85	1.96	13.58	8.15	40.74
55 - 64	2580729	1.72	1.03	2.40	3.75	3.24	4.27	78.58	73.00	84.16	27.90	27.28	28.52	7.98	7.79	8.18	2.52	2.48	2.56	23.25	19.37	54.25
65 - 74	1654901	1.91	1.22	2.60	2.23	1.72	2.75	99.28	93.72	104.84	35.53	34.93	36.14	10.21	10.03	10.39	3.26	3.20	3.32	48.34	30.21	157.11
75 - 84	852824	2.09	1.41	2.78	2.25	1.73	2.76	119.72	114.09	125.35	44.68	44.09	45.26	13.25	13.02	13.48	3.87	3.87	3.87	93.81	82.08	363.50
85 +	266092	1.91	1.22	2.59	2.28	1.76	2.79	134.54	128.90	140.18	52.24	51.49	52.99	16.16	15.78	16.54	4.51	4.51	4.51	75.16	0.00	338.23
Total	38179725	9.08	8.40	9.77	4.18	3.67	4.70	50.49	44.91	56.08	16.87	16.26	17.49	4.51	4.35	4.66	1.34	1.30	1.38	18.60	12.57	78.84

Table S29. Europe, Central: 2005 estimates of AOM and CSOM incidence, HI prevalence and mortality (cases)

Age Groups	Total pop.	AOM incidence			CSOM incidence			HI best ear												Deaths		
								25dB > HI ≤ 40dB			40dB > HI ≤ 60dB			60dB > HI ≤ 80dB			80dB > HI					
		AOM	MIN	MAX	CSOM	MIN	MAX	HI	MIN	MAX	HI	MIN	MAX	HI	MIN	MAX	HI	MIN	MAX	Deaths	MIN	MAX
0-11m.	1187026	302138	293989	310287	9995	9386	10604	80	14	146	20	13	27	2	1	3	0	0	0	1	0	5
1 - 4	4659682	1417782	1385793	1449771	26324	23933	28715	794	534	1054	195	166	224	30	26	34	4	3	5	8	4	30
5 - 9	6238263	773318	730492	816144	29589	26388	32790	1279	931	1627	317	279	355	51	45	57	6	5	7	8	5	17
10 - 14	7336848	485346	434978	535714	27120	23355	30885	1627	1217	2037	402	357	447	65	58	72	10	9	11	3	1	11
15 - 19	8694035	172730	113045	232415	25214	20753	29675	2077	1591	2563	513	460	566	80	72	88	12	11	13	1	1	9
20 - 24	9148898	173166	110359	235973	45623	40928	50318	2484	1973	2995	617	561	673	97	88	106	16	15	17	4	1	10
25 - 34	18456661	187896	61190	314602	58718	49247	68189	5695	4664	6726	1425	1311	1539	227	209	245	36	33	39	4	3	11
35 - 44	15898773	163492	54346	272638	47594	39436	55752	5982	5094	6870	1491	1393	1589	237	221	253	37	35	39	5	4	23
45 - 54	17556060	214597	94074	335120	79214	70206	88222	7471	6490	8452	1868	1760	1976	297	280	314	49	46	52	5	1	11
55 - 64	12802095	168006	80119	255893	50157	43588	56726	6578	5863	7293	1655	1576	1734	267	254	280	43	41	45	7	3	15
65 - 74	10099570	148636	79302	217970	22956	17774	28138	6564	6000	7128	1641	1579	1703	261	251	271	43	41	45	3	2	14
75 - 84	5705896	94497	55326	133668	13023	10095	15951	4485	4166	4804	1129	1094	1164	180	174	186	29	28	30	7	4	20
85 +	965608	15436	8807	22065	2230	1735	2725	854	800	908	216	210	222	34	33	35	5	5	5	3	2	15
Total	118749415	4317040	3501820	5132260	437757	376824	498690	45970	39337	52603	11489	10759	12219	1828	1712	1944	290	272	308	59	31	191

Table S30. Europe, Central: 2005 estimates of AOM and CSOM incidence, HI prevalence and mortality (proportions)

Age Groups	Total pop.	AOM% incidence			CSOM‰ incidence			HI best ear °/°°°° 25dB > HI ≤ 40dB			HI best ear °/°°°° 40dB > HI ≤ 60dB			HI best ear °/°°°° 60dB > HI ≤ 80dB			HI best ear °/°°°° 80dB > HI			Deaths°/°°°°°°		
		AOM	MIN	MAX	CSOM	MIN	MAX	HI	MIN	MAX	HI	MIN	MAX	HI	MIN	MAX	HI	MIN	MAX	Deaths	MIN	MAX
0-11m.	1187026	25.45	24.77	26.14	8.42	7.91	8.93	6.74	1.18	12.30	1.68	1.10	2.27	0.17	0.08	0.25	0.00	0.00	0.00	8.42	0.00	42.12
1 - 4	4659682	30.43	29.74	31.11	5.65	5.14	6.16	17.04	11.46	22.62	4.18	3.56	4.81	0.64	0.56	0.73	0.09	0.06	0.11	17.17	8.58	64.38
5 - 9	6238263	12.40	11.71	13.08	4.74	4.23	5.26	20.50	14.92	26.08	5.08	4.47	5.69	0.82	0.72	0.91	0.10	0.08	0.11	12.82	8.02	27.25
10 - 14	7336848	6.62	5.93	7.30	3.70	3.18	4.21	22.18	16.59	27.76	5.48	4.87	6.09	0.89	0.79	0.98	0.14	0.12	0.15	4.09	1.36	14.99
15 - 19	8694035	1.99	1.30	2.67	2.90	2.39	3.41	23.89	18.30	29.48	5.90	5.29	6.51	0.92	0.83	1.01	0.14	0.13	0.15	1.15	1.15	10.35
20 - 24	9148898	1.89	1.21	2.58	4.99	4.47	5.50	27.15	21.57	32.74	6.74	6.13	7.36	1.06	0.96	1.16	0.17	0.16	0.19	4.37	1.09	10.93
25 - 34	18456661	1.02	0.33	1.70	3.18	2.67	3.69	30.86	25.27	36.44	7.72	7.10	8.34	1.23	1.13	1.33	0.20	0.18	0.21	2.17	1.63	5.96
35 - 44	15898773	1.03	0.34	1.71	2.99	2.48	3.51	37.63	32.04	43.21	9.38	8.76	9.99	1.49	1.39	1.59	0.23	0.22	0.25	3.14	2.52	14.47
45 - 54	17556060	1.22	0.54	1.91	4.51	4.00	5.03	42.56	36.97	48.14	10.64	10.03	11.26	1.69	1.59	1.79	0.28	0.26	0.30	2.85	0.57	6.27
55 - 64	12802095	1.31	0.63	2.00	3.92	3.40	4.43	51.38	45.80	56.97	12.93	12.31	13.54	2.09	1.98	2.19	0.34	0.32	0.35	5.47	2.34	11.72
65 - 74	10099570	1.47	0.79	2.16	2.27	1.76	2.79	64.99	59.41	70.58	16.25	15.63	16.86	2.58	2.49	2.68	0.43	0.41	0.45	2.97	1.98	13.86
75 - 84	5705896	1.66	0.97	2.34	2.28	1.77	2.80	78.60	73.01	84.19	19.79	19.17	20.40	3.15	3.05	3.26	0.51	0.49	0.53	12.27	7.01	35.05
85 +	965608	1.60	0.91	2.29	2.31	1.80	2.82	88.44	82.85	94.03	22.37	21.75	22.99	3.52	3.42	3.62	0.52	0.52	0.52	31.07	20.71	155.34
Total	118749415	3.64	2.95	4.32	3.69	3.17	4.20	38.71	33.13	44.30	9.68	9.06	10.29	1.54	1.44	1.64	0.24	0.23	0.26	4.97	2.61	16.08

Table S31. Europe, Eastern: 2005 estimates of AOM and CSOM incidence, HI prevalence and mortality (cases)

Age Groups	Total pop.	AOM incidence			CSOM incidence			HI best ear 25dB > HI ≤ 40dB			40dB > HI ≤ 60dB			60dB > HI ≤ 80dB			80dB > HI			Deaths		
		AOM	MIN	MAX	CSOM	MIN	MAX	HI	MIN	MAX	HI	MIN	MAX	HI	MIN	MAX	HI	MIN	MAX	Deaths	MIN	MAX
0-11m.	2204620	615903	600768	631038	21440	20309	22571	162	39	285	38	24	52	5	3	7	1	1	1	4	3	6
1 - 4	7836364	2685437	2631640	2739234	50800	46779	54821	1432	994	1870	350	302	398	53	46	60	7	6	8	17	15	32
5 - 9	9666111	1328217	1261859	1394575	52490	47530	57450	2118	1578	2658	517	458	576	77	68	86	13	12	14	9	7	14
10 - 14	12344962	1044548	959799	1129297	46431	40097	52765	2930	2241	3619	716	640	792	111	99	123	16	14	18	10	8	14
15 - 19	17486672	390566	270519	510613	52555	43582	61528	4476	3499	5453	1091	983	1199	166	150	182	24	22	26	5	5	10
20 - 24	17918941	377638	254624	500652	89409	80214	98604	5239	4238	6240	1276	1166	1386	193	176	210	28	26	30	7	5	10
25 - 34	31008604	349147	136272	562022	100456	84545	116367	10179	8447	11911	2479	2288	2670	378	349	407	57	53	61	7	5	14
35 - 44	30513394	346276	136801	555751	93404	77747	109061	12236	10532	13940	2979	2791	3167	454	425	483	69	65	73	13	14	23
45 - 54	32565203	440974	217413	664535	143107	126397	159817	14856	13037	16675	3618	3418	3818	550	520	580	84	79	89	8	5	11
55 - 64	19722900	289158	153760	424556	75106	64986	85226	10779	9677	11881	2631	2510	2752	403	384	422	62	59	65	9	8	15
65 - 74	18843845	307610	178246	436974	42578	32909	52247	13037	11985	14089	3181	3065	3297	486	468	504	75	72	78	10	5	14
75 - 84	9942698	184333	116076	252590	22552	17450	27654	8315	7760	8870	2030	1969	2091	310	301	319	48	47	49	13	12	20
85 +	1559443	28042	17336	38748	3577	2777	4377	1472	1385	1559	359	349	369	54	53	55	7	7	7	9	8	14
Total	211613757	8387849	6935113	9840585	793905	685322	902488	87231	75412	99050	21265	19963	22567	3240	3042	3438	491	463	519	121	100	197

Table S32. Europe, Eastern: 2005 estimates of AOM and CSOM incidence, HI prevalence and mortality (proportions)

Age Groups	Total pop.	AOM% incidence			CSOM‰ incidence			HI best ear °/°°°° 25dB > HI ≤ 40dB			HI best ear °/°°°° 40dB > HI ≤ 60dB			HI best ear °/°°°° 60dB > HI ≤ 80dB			HI best ear °/°°°° 80dB > HI			Deaths°/°°°°°°		
		AOM	MIN	MAX	CSOM	MIN	MAX	HI	MIN	MAX	HI	MIN	MAX	HI	MIN	MAX	HI	MIN	MAX	Deaths	MIN	MAX
0-11m.	2204620	27.94	27.25	28.62	9.73	9.21	10.24	7.35	1.77	12.93	1.72	1.09	2.36	0.23	0.14	0.32	0.05	0.05	0.05	18.14	13.61	27.22
1 - 4	7836364	34.27	33.58	34.96	6.48	5.97	7.00	18.27	12.68	23.86	4.47	3.85	5.08	0.68	0.59	0.77	0.09	0.08	0.10	21.69	19.14	40.84
5 - 9	9666111	13.74	13.05	14.43	5.43	4.92	5.94	21.91	16.33	27.50	5.35	4.74	5.96	0.80	0.70	0.89	0.13	0.12	0.14	9.31	7.24	14.48
10 - 14	12344962	8.46	7.77	9.15	3.76	3.25	4.27	23.73	18.15	29.32	5.80	5.18	6.42	0.90	0.80	1.00	0.13	0.11	0.15	8.10	6.48	11.34
15 - 19	17486672	2.23	1.55	2.92	3.01	2.49	3.52	25.60	20.01	31.18	6.24	5.62	6.86	0.95	0.86	1.04	0.14	0.13	0.15	2.86	2.86	5.72
20 - 24	17918941	2.11	1.42	2.79	4.99	4.48	5.50	29.24	23.65	34.82	7.12	6.51	7.73	1.08	0.98	1.17	0.16	0.15	0.17	3.91	2.79	5.58
25 - 34	31008604	1.13	0.44	1.81	3.24	2.73	3.75	32.83	27.24	38.41	7.99	7.38	8.61	1.22	1.13	1.31	0.18	0.17	0.20	2.26	1.61	4.51
35 - 44	30513394	1.13	0.45	1.82	3.06	2.55	3.57	40.10	34.52	45.68	9.76	9.15	10.38	1.49	1.39	1.58	0.23	0.21	0.24	4.26	4.59	7.54
45 - 54	32565203	1.35	0.67	2.04	4.39	3.88	4.91	45.62	40.03	51.20	11.11	10.50	11.72	1.69	1.60	1.78	0.26	0.24	0.27	2.46	1.54	3.38
55 - 64	19722900	1.47	0.78	2.15	3.81	3.29	4.32	54.65	49.06	60.24	13.34	12.73	13.95	2.04	1.95	2.14	0.31	0.30	0.33	4.56	4.06	7.61
65 - 74	18843845	1.63	0.95	2.32	2.26	1.75	2.77	69.18	63.60	74.77	16.88	16.27	17.50	2.58	2.48	2.67	0.40	0.38	0.41	5.31	2.65	7.43
75 - 84	9942698	1.85	1.17	2.54	2.27	1.76	2.78	83.63	78.05	89.21	20.42	19.80	21.03	3.12	3.03	3.21	0.48	0.47	0.49	13.07	12.07	20.12
85 +	1559443	1.80	1.11	2.48	2.29	1.78	2.81	94.39	88.81	99.97	23.02	22.38	23.66	3.46	3.40	3.53	0.45	0.45	0.45	57.71	51.30	89.78
Total	211613757	3.96	3.28	4.65	3.75	3.24	4.26	41.22	35.64	46.81	10.05	9.43	10.66	1.53	1.44	1.62	0.23	0.22	0.25	5.72	4.73	9.31

Table S33. Europe, Western: 2005 estimates of AOM and CSOM incidence, HI prevalence and mortality (cases)

Age Groups	Total pop.	AOM incidence			CSOM incidence			HI best ear												Deaths		
								25dB > HI ≤ 40dB			40dB > HI ≤ 60dB			60dB > HI ≤ 80dB			80dB > HI					
		AOM	MIN	MAX	CSOM	MIN	MAX	HI	MIN	MAX	HI	MIN	MAX	HI	MIN	MAX	HI	MIN	MAX	Deaths	MIN	MAX
0-11m.	4387085	1596834	1584680	1608224	24008	18458	29671	82	0	327	11	0	38	0	1	1	0	0	0	4	4	4
1 - 4	17321052	8332681	8256629	8408733	63395	48621	78169	818	0	1785	131	24	238	10	12	14	0	0	1	4	4	4
5 - 9	21900496	4050176	4025144	4091827	67364	51628	83001	1245	22	2468	202	67	337	19	17	21	0	0	2	0	0	0
10 - 14	22930531	3367470	3347079	3439555	73075	59211	95195	1416	135	2697	233	92	374	20	20	24	0	0	2	3	3	3
15 - 19	24178872	757211	754392	768449	64536	62863	65583	1614	264	2964	262	113	411	23	22	27	0	0	2	2	2	2
20 - 24	25222454	719883	719519	731881	112774	93991	144732	1934	525	3343	317	162	472	29	27	32	1	1	2	5	4	6
25 - 34	55730772	823490	822375	835233	161199	146336	186069	4804	1691	7917	789	446	1132	73	67	80	6	6	6	8	7	9
35 - 44	64012440	951727	952382	967290	182139	178669	185741	6802	3227	10377	1116	722	1510	103	94	113	8	7	9	28	25	31
45 - 54	55333983	989391	989624	1005464	253525	243943	273376	6651	3561	9741	1087	747	1427	102	92	111	8	7	8	7	6	8
55 - 64	46882397	909970	908950	923622	198071	207432	184520	6755	4137	9373	1104	816	1392	100	93	112	8	7	9	12	11	13
65 - 74	37417632	810549	809818	823027	96781	112249	70220	6851	4761	8941	1119	889	1349	102	95	114	8	7	9	12	11	13
75 - 84	24950333	619531	615005	625144	64680	75177	47025	5450	4057	6843	884	731	1037	83	76	92	7	6	7	22	19	25
85 +	7444638	179797	177901	180827	19512	22724	14212	1828	1412	2244	294	248	340	27	26	31	1	1	2	8	7	9
Total	407712685	24108710	23963498	24409276	1381059	1321302	1457514	46250	23792	69020	7549	5057	10057	691	642	772	47	42	59	115	103	127

Table S34. Europe, Western: 2005 estimates of AOM and CSOM incidence, HI prevalence and mortality (proportions)

Age Groups	Total pop.	AOM% incidence			CSOM‰ incidence			HI best ear °/°°°° 25dB > HI ≤ 40dB			HI best ear °/°°°° 40dB > HI ≤ 60dB			HI best ear °/°°°° 60dB > HI ≤ 80dB			HI best ear °/°°°° 80dB > HI			Deaths°/°°°°°°		
		AOM	MIN	MAX	CSOM	MIN	MAX	HI	MIN	MAX	HI	MIN	MAX	HI	MIN	MAX	HI	MIN	MAX	Deaths	MIN	MAX
0-11m.	4387085	36.40	36.12	36.66	5.47	4.21	6.76	1.87	0.00	7.45	0.25	0.00	0.87	0.00	0.02	0.02	0.00	0.00	0.00	9.12	9.12	9.12
1 - 4	17321052	48.11	47.67	48.55	3.66	2.81	4.51	4.72	0.00	10.31	0.76	0.14	1.37	0.06	0.07	0.08	0.00	0.00	0.01	2.31	2.31	2.31
5 - 9	21900496	18.49	18.38	18.68	3.08	2.36	3.79	5.68	0.10	11.27	0.92	0.31	1.54	0.09	0.08	0.10	0.00	0.00	0.01	0.00	0.00	0.00
10 - 14	22930531	14.69	14.60	15.00	3.19	2.58	4.15	6.18	0.59	11.76	1.02	0.40	1.63	0.09	0.09	0.10	0.00	0.00	0.01	1.31	1.31	1.31
15 - 19	24178872	3.13	3.12	3.18	2.67	2.60	2.71	6.68	1.09	12.26	1.08	0.47	1.70	0.10	0.09	0.11	0.00	0.00	0.01	0.83	0.83	0.83
20 - 24	25222454	2.85	2.85	2.90	4.47	3.73	5.74	7.67	2.08	13.25	1.26	0.64	1.87	0.11	0.11	0.13	0.00	0.00	0.01	1.98	1.59	2.38
25 - 34	55730772	1.48	1.48	1.50	2.89	2.63	3.34	8.62	3.03	14.21	1.42	0.80	2.03	0.13	0.12	0.14	0.01	0.01	0.01	1.44	1.26	1.61
35 - 44	64012440	1.49	1.49	1.51	2.85	2.79	2.90	10.63	5.04	16.21	1.74	1.13	2.36	0.16	0.15	0.18	0.01	0.01	0.01	4.37	3.91	4.84
45 - 54	55333983	1.79	1.79	1.82	4.58	4.41	4.94	12.02	6.44	17.60	1.96	1.35	2.58	0.18	0.17	0.20	0.01	0.01	0.01	1.27	1.08	1.45
55 - 64	46882397	1.94	1.94	1.97	4.22	4.42	3.94	14.41	8.82	19.99	2.35	1.74	2.97	0.21	0.20	0.24	0.02	0.01	0.02	2.56	2.35	2.77
65 - 74	37417632	2.17	2.16	2.20	2.59	3.00	1.88	18.31	12.72	23.90	2.99	2.38	3.61	0.27	0.25	0.30	0.02	0.02	0.02	3.21	2.94	3.47
75 - 84	24950333	2.48	2.46	2.51	2.59	3.01	1.88	21.84	16.26	27.43	3.54	2.93	4.16	0.33	0.30	0.37	0.03	0.02	0.03	8.82	7.62	10.02
85 +	7444638	2.42	2.39	2.43	2.62	3.05	1.91	24.55	18.97	30.14	3.95	3.33	4.57	0.36	0.35	0.42	0.01	0.01	0.03	10.75	9.40	12.09
Total	407712685	5.91	5.88	5.99	3.39	3.24	3.57	11.34	5.84	16.93	1.85	1.24	2.47	0.17	0.16	0.19	0.01	0.01	0.01	2.82	2.53	3.11

Table S35. Latin America, Andean: 2005 estimates of AOM and CSOM incidence, HI prevalence and mortality (cases)

Age Groups	Total pop.	AOM incidence			CSOM incidence			HI best ear: 25dB > HI ≤ 40dB			HI best ear: 40dB > HI ≤ 60dB			HI best ear: 60dB > HI ≤ 80dB			HI best ear: 80dB > HI			Deaths		
		AOM	MIN	MAX	CSOM	MIN	MAX	HI	MIN	MAX	HI	MIN	MAX	HI	MIN	MAX	HI	MIN	MAX	Deaths	MIN	MAX
0-11m.	1106885	354995	347396	362594	8252	7684	8820	68	6	130	37	30	44	17	14	20	8	7	9	4	4	4
1 - 4	4379499	1287294	1257229	1317359	20202	17955	22449	671	426	916	369	342	396	166	154	178	75	70	80	17	16	17
5 - 9	5484698	699029	661376	736682	9273	6459	12087	1008	702	1314	554	520	588	250	235	265	112	105	119	12	10	12
10 - 14	5450610	205280	167861	242699	7814	5017	10611	1084	780	1388	597	563	631	268	253	283	121	114	128	5	7	5
15 - 19	5055885	44170	9461	78879	5262	2668	7856	1080	798	1362	595	564	626	269	255	283	122	116	128	3	2	3
20 - 24	4557200	37244	5959	68529	7839	5501	10177	1112	857	1367	612	584	640	277	264	290	125	119	131	2	2	2
25 - 34	7787999	13192	12295	66657	8213	4217	12209	2136	1701	2571	1176	1128	1224	532	510	554	241	231	251	4	6	4
35 - 44	5964131	9878	9206	50822	5605	2545	8665	1996	1663	2329	1101	1064	1138	498	481	515	225	218	232	4	2	4
45 - 54	4293353	6997	6521	36471	6300	4097	8503	1633	1393	1873	901	875	927	407	395	419	185	180	190	2	2	2
55 - 64	2732158	4433	4132	23189	3503	2101	4905	1248	1095	1401	688	671	705	311	303	319	141	138	144	3	2	3
65 - 74	1719833	2765	2577	14572	1232	350	2114	994	898	1090	548	537	559	248	243	253	112	110	114	3	4	3
75 - 84	813784	1281	1194	6868	569	151	987	568	523	613	314	309	319	142	140	144	64	63	65	4	3	4
85 +	171239	266	248	1442	119	31	207	133	123	143	75	74	76	34	34	34	15	15	15	0	1	0
Total	49517274	2666824	2485455	3006763	84183	58776	109590	13731	10965	16497	7567	7261	7873	3419	3281	3557	1546	1486	1606	63	61	63

Table S36. Latin America, Andean: 2005 estimates of AOM and CSOM incidence, HI prevalence and mortality (proportions)

Age Groups	Total pop.	AOM% incidence			CSOM‰ incidence			HI best ear °/°°°°												Deaths°/°°°°°°		
								25dB > HI ≤ 40dB			40dB > HI ≤ 60dB			60dB > HI ≤ 80dB			80dB > HI					
		AOM	MIN	MAX	CSOM	MIN	MAX	HI	MIN	MAX	HI	MIN	MAX	HI	MIN	MAX	HI	MIN	MAX	Deaths	MIN	MAX
0-11m.	1106885	32.07	31.39	32.76	7.46	6.94	7.97	6.14	0.54	11.74	3.34	2.71	3.98	1.54	1.26	1.81	0.72	0.63	0.81	36.14	36.14	36.14
1 - 4	4379499	29.39	28.71	30.08	4.61	4.10	5.13	15.32	9.73	20.92	8.43	7.81	9.04	3.79	3.52	4.06	1.71	1.60	1.83	38.82	36.53	38.82
5 - 9	5484698	12.75	12.06	13.43	1.69	1.18	2.20	18.38	12.80	23.96	10.10	9.48	10.72	4.56	4.28	4.83	2.04	1.91	2.17	21.88	18.23	21.88
10 - 14	5450610	3.77	3.08	4.45	1.43	0.92	1.95	19.89	14.31	25.47	10.95	10.33	11.58	4.92	4.64	5.19	2.22	2.09	2.35	9.17	12.84	9.17
15 - 19	5055885	0.87	0.19	1.56	1.04	0.53	1.55	21.36	15.78	26.94	11.77	11.16	12.38	5.32	5.04	5.60	2.41	2.29	2.53	5.93	3.96	5.93
20 - 24	4557200	0.82	0.13	1.50	1.72	1.21	2.23	24.40	18.81	30.00	13.43	12.81	14.04	6.08	5.79	6.36	2.74	2.61	2.87	4.39	4.39	4.39
25 - 34	7787999	0.17	0.16	0.86	1.05	0.54	1.57	27.43	21.84	33.01	15.10	14.48	15.72	6.83	6.55	7.11	3.09	2.97	3.22	5.14	7.70	5.14
35 - 44	5964131	0.17	0.15	0.85	0.94	0.43	1.45	33.47	27.88	39.05	18.46	17.84	19.08	8.35	8.06	8.63	3.77	3.66	3.89	6.71	3.35	6.71
45 - 54	4293353	0.16	0.15	0.85	1.47	0.95	1.98	38.04	32.45	43.63	20.99	20.38	21.59	9.48	9.20	9.76	4.31	4.19	4.43	4.66	4.66	4.66
55 - 64	2732158	0.16	0.15	0.85	1.28	0.77	1.80	45.68	40.08	51.28	25.18	24.56	25.80	11.38	11.09	11.68	5.16	5.05	5.27	10.98	7.32	10.98
65 - 74	1719833	0.16	0.15	0.85	0.72	0.20	1.23	57.80	52.21	63.38	31.86	31.22	32.50	14.42	14.13	14.71	6.51	6.40	6.63	17.44	23.26	17.44
75 - 84	813784	0.16	0.15	0.84	0.70	0.19	1.21	69.80	64.27	75.33	38.59	37.97	39.20	17.45	17.20	17.70	7.86	7.74	7.99	49.15	36.86	49.15
85 +	171239	0.16	0.14	0.84	0.69	0.18	1.21	77.67	71.83	83.51	43.80	43.21	44.38	19.86	19.86	19.86	8.76	8.76	8.76	0.00	58.40	0.00
Total	49517274	5.39	5.02	6.07	1.70	1.19	2.21	27.73	22.14	33.32	15.28	14.66	15.90	6.90	6.63	7.18	3.12	3.00	3.24	12.72	12.32	12.72

Table S37. Latin America, Central: 2005 estimates of AOM and CSOM incidence, HI prevalence and mortality (cases)

Age Groups	Total pop.	AOM incidence			CSOM incidence			HI best ear												Deaths		
								25dB > HI ≤ 40dB			40dB > HI ≤ 60dB			60dB > HI ≤ 80dB			80dB > HI					
		AOM	MIN	MAX	CSOM	MIN	MAX	HI	MIN	MAX	HI	MIN	MAX	HI	MIN	MAX	HI	MIN	MAX	Deaths	MIN	MAX
0-11m.	4669508	1288718	1256662	1320774	38499	36103	40895	398	137	659	105	76	134	17	12	22	3	2	4	37	27	50
1 - 4	18381028	6193003	6066817	6319189	100978	91546	110410	3926	2899	4953	1039	926	1152	186	166	206	34	30	38	12	8	14
5 - 9	22956225	3099533	2941938	3257128	105436	93657	117215	5898	4616	7180	1568	1427	1709	282	257	307	51	46	56	4	5	7
10 - 14	22823346	1847582	1690899	2004265	84042	72331	95753	6370	5095	7645	1702	1562	1842	307	282	332	56	51	61	4	4	6
15 - 19	21053425	459593	315061	604125	60481	49678	71284	6329	5153	7505	1696	1566	1826	305	282	328	55	51	59	4	4	8
20 - 24	19408444	399622	266382	532862	96766	86807	106725	6685	5601	7769	1799	1680	1918	324	302	346	59	55	63	11	6	16
25 - 34	35038602	384517	143976	625058	110992	93013	128971	13653	11696	15610	3709	3493	3925	674	635	713	125	118	132	31	23	42
35 - 44	27421514	301954	113704	490204	81555	67484	95626	13126	11595	14657	3588	3419	3757	658	627	689	123	117	129	37	28	48
45 - 54	19768455	259413	123702	395124	89852	79708	99996	10762	9658	11866	2947	2825	3069	539	517	561	101	97	105	26	18	37
55 - 64	12073437	171814	88929	254699	47568	41373	53763	7865	7191	8539	2144	2070	2218	392	378	406	73	70	76	21	17	30
65 - 74	7107713	112511	63716	161306	16182	12535	19829	5858	5461	6255	1596	1552	1640	291	283	299	54	53	55	30	23	42
75 - 84	3606145	64619	39863	89375	8245	6395	10095	3603	3402	3804	985	963	1007	181	177	185	32	31	33	43	30	56
85 +	863222	14921	8995	20847	1999	1556	2442	980	932	1028	271	266	276	49	48	50	8	8	8	6	4	9
Total	215171064	14597800	13120644	16074956	842595	732186	953004	85453	73436	97470	23149	21825	24473	4205	3966	4444	774	729	819	266	197	365

Table S38. Latin America, Central: 2005 estimates of AOM and CSOM incidence, HI prevalence and mortality (proportions)

Age Groups	Total pop.	AOM% incidence			CSOM‰ incidence			HI best ear °/°°°°												Deaths°/°°°°°°		
								25dB > HI ≤ 40dB			40dB > HI ≤ 60dB			60dB > HI ≤ 80dB			80dB > HI					
		AOM	MIN	MAX	CSOM	MIN	MAX	HI	MIN	MAX	HI	MIN	MAX	HI	MIN	MAX	HI	MIN	MAX	Deaths	MIN	MAX
0-11m.	4669508	27.60	26.91	28.29	8.24	7.73	8.76	8.52	2.93	14.11	2.25	1.63	2.87	0.36	0.26	0.47	0.06	0.04	0.09	79.24	57.82	107.08
1 - 4	18381028	33.69	33.01	34.38	5.49	4.98	6.01	21.36	15.77	26.95	5.65	5.04	6.27	1.01	0.90	1.12	0.18	0.16	0.21	6.53	4.35	7.62
5 - 9	22956225	13.50	12.82	14.19	4.59	4.08	5.11	25.69	20.11	31.28	6.83	6.22	7.44	1.23	1.12	1.34	0.22	0.20	0.24	1.74	2.18	3.05
10 - 14	22823346	8.10	7.41	8.78	3.68	3.17	4.20	27.91	22.32	33.50	7.46	6.84	8.07	1.35	1.24	1.45	0.25	0.22	0.27	1.75	1.75	2.63
15 - 19	21053425	2.18	1.50	2.87	2.87	2.36	3.39	30.06	24.48	35.65	8.06	7.44	8.67	1.45	1.34	1.56	0.26	0.24	0.28	1.90	1.90	3.80
20 - 24	19408444	2.06	1.37	2.75	4.99	4.47	5.50	34.44	28.86	40.03	9.27	8.66	9.88	1.67	1.56	1.78	0.30	0.28	0.32	5.67	3.09	8.24
25 - 34	35038602	1.10	0.41	1.78	3.17	2.65	3.68	38.97	33.38	44.55	10.59	9.97	11.20	1.92	1.81	2.03	0.36	0.34	0.38	8.85	6.56	11.99
35 - 44	27421514	1.10	0.41	1.79	2.97	2.46	3.49	47.87	42.28	53.45	13.08	12.47	13.70	2.40	2.29	2.51	0.45	0.43	0.47	13.49	10.21	17.50
45 - 54	19768455	1.31	0.63	2.00	4.55	4.03	5.06	54.44	48.86	60.02	14.91	14.29	15.52	2.73	2.62	2.84	0.51	0.49	0.53	13.15	9.11	18.72
55 - 64	12073437	1.42	0.74	2.11	3.94	3.43	4.45	65.14	59.56	70.73	17.76	17.15	18.37	3.25	3.13	3.36	0.60	0.58	0.63	17.39	14.08	24.85
65 - 74	7107713	1.58	0.90	2.27	2.28	1.76	2.79	82.42	76.83	88.00	22.45	21.84	23.07	4.09	3.98	4.21	0.76	0.75	0.77	42.21	32.36	59.09
75 - 84	3606145	1.79	1.11	2.48	2.29	1.77	2.80	99.91	94.34	105.49	27.31	26.70	27.92	5.02	4.91	5.13	0.89	0.86	0.92	119.24	83.19	155.29
85 +	863222	1.73	1.04	2.42	2.32	1.80	2.83	113.53	107.97	119.09	31.39	30.81	31.97	5.68	5.56	5.79	0.93	0.93	0.93	69.51	46.34	104.26
Total	215171064	6.78	6.10	7.47	3.92	3.40	4.43	39.71	34.13	45.30	10.76	10.14	11.37	1.95	1.84	2.07	0.36	0.34	0.38	12.36	9.16	16.96

Table S39. Latin America, Southern: 2005 estimates of AOM and CSOM incidence, HI prevalence and mortality (cases)

Age Groups	Total pop.	AOM incidence			CSOM incidence			HI best ear												Deaths		
								25dB > HI ≤ 40dB			40dB > HI ≤ 60dB			60dB > HI ≤ 80dB			80dB > HI					
		AOM	MIN	MAX	CSOM	MIN	MAX	HI	MIN	MAX	HI	MIN	MAX	HI	MIN	MAX	HI	MIN	MAX	Deaths	MIN	MAX
0-11m.	964398	217791	211170	224412	6369	5874	6864	74	20	128	19	13	25	3	2	4	0	0	0	1	1	3
1 - 4	3870150	989341	962772	1015910	17081	15095	19067	746	530	962	192	168	216	31	27	35	5	4	6	7	4	15
5 - 9	5022850	537031	502549	571513	18578	16001	21155	1162	881	1443	300	269	331	49	44	54	8	7	9	5	4	9
10 - 14	5225632	227360	191486	263234	18619	15938	21300	1312	1020	1604	340	308	372	57	52	62	10	9	11	1	0	4
15 - 19	5090054	83588	48645	118531	13940	11328	16552	1377	1093	1661	356	325	387	59	54	64	10	9	11	1	2	3
20 - 24	4796116	77285	44360	110210	23652	21191	26113	1481	1213	1749	382	352	412	64	59	69	11	10	12	1	2	1
25 - 34	8909889	79456	18289	140623	27526	22954	32098	3095	2597	3593	799	744	854	132	123	141	22	20	24	2	1	4
35 - 44	7597126	67437	15282	119592	22009	18111	25907	3249	2825	3673	847	800	894	143	135	151	25	24	26	2	1	6
45 - 54	6421096	67818	23737	111899	29882	26587	33177	3111	2752	3470	808	768	848	135	128	142	23	22	24	2	2	3
55 - 64	4755163	54498	21854	87142	19323	16883	21763	2756	2490	3022	712	683	741	118	113	123	20	19	21	1	0	6
65 - 74	3285010	41956	19404	64508	7605	5919	9291	2406	2223	2589	620	600	640	102	99	105	17	16	18	3	2	8
75 - 84	1901238	27550	14498	40602	4416	3440	5392	1683	1577	1789	432	420	444	71	69	73	11	11	11	7	4	11
85 +	532210	7458	3804	11112	1253	980	1526	534	504	564	138	135	141	22	21	23	3	3	3	2	0	6
Total	58370932	2478569	2077850	2879288	210253	180301	240205	22986	19725	26247	5945	5585	6305	986	926	1046	165	154	176	35	23	79

Table S40. Latin America, Southern: 2005 estimates of AOM and CSOM incidence, HI prevalence and mortality (proportions)

Age Groups	Total pop.	AOM% incidence			CSOM‰ incidence			HI best ear °/°°°° 25dB > HI ≤ 40dB			40dB > HI ≤ 60dB			60dB > HI ≤ 80dB			80dB > HI			Deaths°/°°°°°°		
		AOM	MIN	MAX	CSOM	MIN	MAX	HI	MIN	MAX	HI	MIN	MAX	HI	MIN	MAX	HI	MIN	MAX	Deaths	MIN	MAX
0-11m.	964398	22.58	21.90	23.27	6.60	6.09	7.12	7.67	2.07	13.27	1.97	1.35	2.59	0.31	0.21	0.41	0.00	0.00	0.00	10.37	10.37	31.11
1 - 4	3870150	25.56	24.88	26.25	4.41	3.90	4.93	19.28	13.69	24.86	4.96	4.34	5.58	0.80	0.70	0.90	0.13	0.10	0.16	18.09	10.34	38.76
5 - 9	5022850	10.69	10.01	11.38	3.70	3.19	4.21	23.13	17.54	28.73	5.97	5.36	6.59	0.98	0.88	1.08	0.16	0.14	0.18	9.95	7.96	17.92
10 - 14	5225632	4.35	3.66	5.04	3.56	3.05	4.08	25.11	19.52	30.69	6.51	5.89	7.12	1.09	1.00	1.19	0.19	0.17	0.21	1.91	0.00	7.65
15 - 19	5090054	1.64	0.96	2.33	2.74	2.23	3.25	27.05	21.47	32.63	6.99	6.39	7.60	1.16	1.06	1.26	0.20	0.18	0.22	1.96	3.93	5.89
20 - 24	4796116	1.61	0.92	2.30	4.93	4.42	5.44	30.88	25.29	36.47	7.96	7.34	8.59	1.33	1.23	1.44	0.23	0.21	0.25	2.09	4.17	2.09
25 - 34	8909889	0.89	0.21	1.58	3.09	2.58	3.60	34.74	29.15	40.33	8.97	8.35	9.58	1.48	1.38	1.58	0.25	0.22	0.27	2.24	1.12	4.49
35 - 44	7597126	0.89	0.20	1.57	2.90	2.38	3.41	42.77	37.19	48.35	11.15	10.53	11.77	1.88	1.78	1.99	0.33	0.32	0.34	2.63	1.32	7.90
45 - 54	6421096	1.06	0.37	1.74	4.65	4.14	5.17	48.45	42.86	54.04	12.58	11.96	13.21	2.10	1.99	2.21	0.36	0.34	0.37	3.11	3.11	4.67
55 - 64	4755163	1.15	0.46	1.83	4.06	3.55	4.58	57.96	52.36	63.55	14.97	14.36	15.58	2.48	2.38	2.59	0.42	0.40	0.44	2.10	0.00	12.62
65 - 74	3285010	1.28	0.59	1.96	2.32	1.80	2.83	73.24	67.67	78.81	18.87	18.26	19.48	3.11	3.01	3.20	0.52	0.49	0.55	9.13	6.09	24.35
75 - 84	1901238	1.45	0.76	2.14	2.32	1.81	2.84	88.52	82.95	94.10	22.72	22.09	23.35	3.73	3.63	3.84	0.58	0.58	0.58	36.82	21.04	57.86
85 +	532210	1.40	0.71	2.09	2.35	1.84	2.87	100.34	94.70	105.97	25.93	25.37	26.49	4.13	3.95	4.32	0.56	0.56	0.56	37.58	0.00	112.74
Total	58370932	4.25	3.56	4.93	3.60	3.09	4.12	39.38	33.79	44.97	10.18	9.57	10.80	1.69	1.59	1.79	0.28	0.26	0.30	6.00	3.94	13.53

Table S41. Latin America, Tropical: 2005 estimates of AOM and CSOM incidence, HI prevalence and mortality (cases)

Age Groups	Total pop.	AOM incidence			CSOM incidence			HI best ear												Deaths		
								25dB > HI ≤ 40dB			40dB > HI ≤ 60dB			60dB > HI ≤ 80dB			80dB > HI					
		AOM	MIN	MAX	CSOM	MIN	MAX	HI	MIN	MAX	HI	MIN	MAX	HI	MIN	MAX	HI	MIN	MAX	Deaths	MIN	MAX
0-11m.	3758292	996378	970577	1022179	26828	24900	28756	301	91	511	76	53	99	12	8	16	2	1	3	42	39	47
1 - 4	15013023	4795146	4692081	4898211	71497	63793	79201	3000	2162	3838	762	670	854	123	108	138	20	18	22	8	8	9
5 - 9	18049065	2333122	2209215	2457029	72192	62931	81453	4328	3320	5336	1099	988	1210	177	159	195	28	25	31	8	8	9
10 - 14	17289186	1275084	1156393	1393775	62749	53878	71620	4490	3524	5456	1141	1035	1247	184	167	201	29	26	32	15	13	16
15 - 19	18037636	374765	250936	498594	50224	40968	59480	5047	4040	6054	1283	1172	1394	207	189	225	33	30	36	11	10	12
20 - 24	18535201	365624	238379	492869	92366	82855	101877	5931	4896	6966	1508	1394	1622	243	225	261	40	37	43	15	13	16
25 - 34	31433208	333393	117603	549183	98291	82162	114420	11319	9563	13075	2881	2688	3074	466	435	497	75	70	80	15	13	16
35 - 44	26733415	285665	102139	469191	78258	64540	91976	11769	10276	13262	2998	2834	3162	484	457	511	79	75	83	21	19	24
45 - 54	19964525	254189	117132	391246	92323	82079	102567	9988	8873	11103	2543	2420	2666	411	391	431	67	64	70	13	12	14
55 - 64	12181312	167557	83932	251182	48906	42655	55157	7312	6632	7992	1863	1788	1938	301	289	313	48	46	50	19	17	21
65 - 74	7268508	111116	61217	161015	16619	12889	20349	5528	5122	5934	1407	1362	1452	228	221	235	36	35	37	8	8	9
75 - 84	3484282	60408	36488	84328	7999	6211	9787	3207	3012	3402	816	795	837	132	129	135	21	20	22	8	8	9
85 +	987443	16600	9821	23379	2295	1788	2802	1028	973	1083	262	256	268	42	41	43	7	7	7	2	2	2
Total	192735096	11369047	10045913	12692181	720547	621649	819445	73248	62484	84012	18639	17455	19823	3010	2819	3201	485	454	516	185	170	204

Table S42. Latin America, Tropical: 2005 estimates of AOM and CSOM incidence, HI prevalence and mortality (proportions)

Age Groups	Total pop.	AOM% incidence			CSOM‰ incidence			HI best ear °/°°°°												Deaths°/°°°°°°		
								25dB > HI ≤ 40dB			40dB > HI ≤ 60dB			60dB > HI ≤ 80dB			80dB > HI					
		AOM	MIN	MAX	CSOM	MIN	MAX	HI	MIN	MAX	HI	MIN	MAX	HI	MIN	MAX	HI	MIN	MAX	Deaths	MIN	MAX
0-11m.	3758292	26.51	25.82	27.20	7.14	6.63	7.65	8.01	2.42	13.60	2.02	1.41	2.63	0.32	0.21	0.43	0.05	0.03	0.08	111.75	103.77	125.06
1 - 4	15013023	31.94	31.25	32.63	4.76	4.25	5.28	19.98	14.40	25.56	5.08	4.46	5.69	0.82	0.72	0.92	0.13	0.12	0.15	5.33	5.33	5.99
5 - 9	18049065	12.93	12.24	13.61	4.00	3.49	4.51	23.98	18.39	29.56	6.09	5.47	6.70	0.98	0.88	1.08	0.16	0.14	0.17	4.43	4.43	4.99
10 - 14	17289186	7.38	6.69	8.06	3.63	3.12	4.14	25.97	20.38	31.56	6.60	5.99	7.21	1.06	0.97	1.16	0.17	0.15	0.19	8.68	7.52	9.25
15 - 19	18037636	2.08	1.39	2.76	2.78	2.27	3.30	27.98	22.40	33.56	7.11	6.50	7.73	1.15	1.05	1.25	0.18	0.17	0.20	6.10	5.54	6.65
20 - 24	18535201	1.97	1.29	2.66	4.98	4.47	5.50	32.00	26.41	37.58	8.14	7.52	8.75	1.31	1.21	1.41	0.22	0.20	0.23	8.09	7.01	8.63
25 - 34	31433208	1.06	0.37	1.75	3.13	2.61	3.64	36.01	30.42	41.60	9.17	8.55	9.78	1.48	1.38	1.58	0.24	0.22	0.25	4.77	4.14	5.09
35 - 44	26733415	1.07	0.38	1.76	2.93	2.41	3.44	44.02	38.44	49.61	11.21	10.60	11.83	1.81	1.71	1.91	0.30	0.28	0.31	7.86	7.11	8.98
45 - 54	19964525	1.27	0.59	1.96	4.62	4.11	5.14	50.03	44.44	55.61	12.74	12.12	13.35	2.06	1.96	2.16	0.34	0.32	0.35	6.51	6.01	7.01
55 - 64	12181312	1.38	0.69	2.06	4.01	3.50	4.53	60.03	54.44	65.61	15.29	14.68	15.91	2.47	2.37	2.57	0.39	0.38	0.41	15.60	13.96	17.24
65 - 74	7268508	1.53	0.84	2.22	2.29	1.77	2.80	76.05	70.47	81.64	19.36	18.74	19.98	3.14	3.04	3.23	0.50	0.48	0.51	11.01	11.01	12.38
75 - 84	3484282	1.73	1.05	2.42	2.30	1.78	2.81	92.04	86.45	97.64	23.42	22.82	24.02	3.79	3.70	3.87	0.60	0.57	0.63	22.96	22.96	25.83
85 +	987443	1.68	0.99	2.37	2.32	1.81	2.84	104.11	98.54	109.68	26.53	25.93	27.14	4.25	4.15	4.35	0.71	0.71	0.71	20.25	20.25	20.25
Total	192735096	5.90	5.21	6.59	3.74	3.23	4.25	38.00	32.42	43.59	9.67	9.06	10.29	1.56	1.46	1.66	0.25	0.24	0.27	9.60	8.82	10.58

Table S43. North Africa / Middle East: 2005 estimates of AOM and CSOM incidence, HI prevalence and mortality (cases)

Age Groups	Total pop.	AOM incidence			CSOM incidence			HI best ear												Deaths		
								25dB > HI ≤ 40dB			40dB > HI ≤ 60dB			60dB > HI ≤ 80dB			80dB > HI					
		AOM	MIN	MAX	CSOM	MIN	MAX	HI	MIN	MAX	HI	MIN	MAX	HI	MIN	MAX	HI	MIN	MAX	Deaths	MIN	MAX
0-11m.	9398021	3116453	3051935	3180971	106953	102131	111775	1248	723	1773	436	378	494	109	95	123	26	23	29	44	33	50
1 - 4	35268179	15058582	14816465	15300699	267090	248993	285187	11682	9712	13652	4090	3873	4307	1028	973	1083	260	246	274	187	160	234
5 - 9	43009232	7117225	6821965	7412485	270369	248300	292438	17079	14677	19481	5996	5731	6261	1508	1441	1575	386	369	403	101	85	127
10 - 14	44695141	5347379	5040545	5654213	171166	148232	194100	19248	16752	21744	6785	6510	7060	1715	1646	1784	441	423	459	77	65	94
15 - 19	44611815	1203204	896943	1509465	138011	115120	160902	20652	18160	23144	7304	7030	7578	1856	1786	1926	480	462	498	43	38	53
20 - 24	42379881	1045123	754184	1336062	211545	189799	233291	22343	19976	24710	7935	7674	8196	2024	1958	2090	524	507	541	35	27	43
25 - 34	68471388	875722	405664	1345780	223540	188406	258674	40268	36444	44092	14320	13899	14741	3667	3559	3775	953	925	981	63	54	77
35 - 44	50105849	641633	297655	985611	154496	128785	180207	35991	33193	38789	12835	12527	13143	3297	3218	3376	859	838	880	78	67	94
45 - 54	35110764	534639	293603	775675	153353	135337	171369	28883	26922	30844	10371	10155	10587	2682	2626	2738	702	687	717	43	38	56
55 - 64	19759048	325370	189724	461016	74756	64617	84895	19543	18439	20647	7024	6902	7146	1818	1787	1849	477	469	485	46	41	60
65 - 74	12374629	225797	140845	310749	27942	21592	34292	15523	14832	16214	5596	5520	5672	1452	1432	1472	383	378	388	62	50	77
75 - 84	4998098	103196	68884	137508	11332	8767	13897	7583	7304	7862	2738	2707	2769	712	704	720	187	185	189	94	81	118
85 +	617580	12615	8375	16855	1412	1095	1729	1061	1027	1095	382	378	386	98	97	99	26	26	26	29	20	33
Total	410799625	35606938	32786787	38427089	1811965	1601174	2022756	241104	218161	264047	85812	83284	88340	21966	21322	22610	5704	5538	5870	902	759	1116

Table S44. North Africa / Middle East: 2005 estimates of AOM and CSOM incidence, HI prevalence and mortality (proportions)

Age Groups	Total pop.	AOM% incidence			CSOM‰ incidence			HI best ear °/°°°°												Deaths°/°°°°°°		
								25dB > HI ≤ 40dB			40dB > HI ≤ 60dB			60dB > HI ≤ 80dB			80dB > HI					
		AOM	MIN	MAX	CSOM	MIN	MAX	HI	MIN	MAX	HI	MIN	MAX	HI	MIN	MAX	HI	MIN	MAX	Deaths	MIN	MAX
0-11m.	9398021	33.16	32.47	33.85	11.38	10.87	11.89	13.28	7.69	18.87	4.64	4.02	5.26	1.16	1.01	1.31	0.28	0.24	0.31	46.82	35.11	53.20
1 - 4	35268179	42.70	42.01	43.38	7.57	7.06	8.09	33.12	27.54	38.71	11.60	10.98	12.21	2.91	2.76	3.07	0.74	0.70	0.78	53.02	45.37	66.35
5 - 9	43009232	16.55	15.86	17.23	6.29	5.77	6.80	39.71	34.13	45.29	13.94	13.33	14.56	3.51	3.35	3.66	0.90	0.86	0.94	23.48	19.76	29.53
10 - 14	44695141	11.96	11.28	12.65	3.83	3.32	4.34	43.07	37.48	48.65	15.18	14.57	15.80	3.84	3.68	3.99	0.99	0.95	1.03	17.23	14.54	21.03
15 - 19	44611815	2.70	2.01	3.38	3.09	2.58	3.61	46.29	40.71	51.88	16.37	15.76	16.99	4.16	4.00	4.32	1.08	1.04	1.12	9.64	8.52	11.88
20 - 24	42379881	2.47	1.78	3.15	4.99	4.48	5.50	52.72	47.14	58.31	18.72	18.11	19.34	4.78	4.62	4.93	1.24	1.20	1.28	8.26	6.37	10.15
25 - 34	68471388	1.28	0.59	1.97	3.26	2.75	3.78	58.81	53.23	64.39	20.91	20.30	21.53	5.36	5.20	5.51	1.39	1.35	1.43	9.20	7.89	11.25
35 - 44	50105849	1.28	0.59	1.97	3.08	2.57	3.60	71.83	66.25	77.41	25.62	25.00	26.23	6.58	6.42	6.74	1.71	1.67	1.76	15.57	13.37	18.76
45 - 54	35110764	1.52	0.84	2.21	4.37	3.85	4.88	82.26	76.68	87.85	29.54	28.92	30.15	7.64	7.48	7.80	2.00	1.96	2.04	12.25	10.82	15.95
55 - 64	19759048	1.65	0.96	2.33	3.78	3.27	4.30	98.91	93.32	104.49	35.55	34.93	36.17	9.20	9.04	9.36	2.41	2.37	2.45	23.28	20.75	30.37
65 - 74	12374629	1.82	1.14	2.51	2.26	1.74	2.77	125.44	119.86	131.03	45.22	44.61	45.84	11.73	11.57	11.90	3.10	3.05	3.14	50.10	40.41	62.22
75 - 84	4998098	2.06	1.38	2.75	2.27	1.75	2.78	151.72	146.14	157.30	54.78	54.16	55.40	14.25	14.09	14.41	3.74	3.70	3.78	188.07	162.06	236.09
85 +	617580	2.04	1.36	2.73	2.29	1.77	2.80	171.80	166.29	177.30	61.85	61.21	62.50	15.87	15.71	16.03	4.21	4.21	4.21	469.57	323.84	534.34
Total	410799625	8.67	7.98	9.35	4.41	3.90	4.92	58.69	53.11	64.28	20.89	20.27	21.50	5.35	5.19	5.50	1.39	1.35	1.43	21.96	18.48	27.17

Table S45. North America, High Income: 2005 estimates of AOM and CSOM incidence, HI prevalence and mortality (cases)

Age Groups	Total pop.	AOM incidence			CSOM incidence			HI best ear												Deaths		
								25dB > HI ≤ 40dB			40dB > HI ≤ 60dB			60dB > HI ≤ 80dB			80dB > HI					
		AOM	MIN	MAX	CSOM	MIN	MAX	HI	MIN	MAX	HI	MIN	MAX	HI	MIN	MAX	HI	MIN	MAX	Deaths	MIN	MAX
0-11m.	4673395	1411741	1399018	1424098	15589	9615	21560	98	0	359	19	0	48	2	0	5	0	0	0	9	9	13
1 - 4	17635275	6698845	6621414	6776276	39240	24198	54282	922	0	1907	179	71	287	20	8	32	2	1	3	3	2	4
5 - 9	22102033	3318235	3286505	3353801	41291	25470	57132	1388	154	2622	270	134	406	31	15	47	4	2	6	3	1	1
10 - 14	23699731	2400083	2357690	2453268	48486	29915	67105	1614	290	2938	314	168	460	36	19	53	4	2	6	2	1	1
15 - 19	23871979	590989	584853	598731	61147	59806	62492	1751	418	3084	340	193	487	38	22	54	5	3	7	1	1	1
20 - 24	23226793	536211	531373	542757	70573	47249	93976	1948	651	3245	379	236	522	43	27	59	5	3	7	1	2	2
25 - 34	44886176	547191	542975	553332	106910	90942	122943	4237	1730	6744	825	549	1101	94	63	125	10	7	13	4	3	5
35 - 44	49268828	605156	600767	612241	135653	132939	138382	5689	2937	8441	1110	807	1413	127	92	162	14	10	18	6	4	6
45 - 54	47877462	703555	698286	711992	202349	189649	215116	6281	3607	8955	1226	931	1521	141	107	175	16	12	20	6	3	5
55 - 64	33890309	538814	534808	545414	155621	163880	147318	5337	3444	7230	1042	834	1250	119	95	143	14	11	17	2	2	4
65 - 74	20924698	370439	367729	375116	70825	82542	59039	4175	3006	5344	816	687	945	94	79	109	11	9	13	4	3	5
75 - 84	14534112	292469	290176	296082	49408	57586	41187	3508	2696	4320	684	595	773	78	68	88	9	8	10	6	5	7
85 +	5526168	107973	107021	109193	19028	22183	15865	1505	1196	1814	292	258	326	33	29	37	4	4	4	7	5	9
Total	332116959	18121701	17922615	18352301	1016120	935974	1096397	38453	20129	57003	7496	5463	9539	856	624	1089	98	72	124	54	41	63

Table S46. North America, High Income: 2005 estimates of AOM and CSOM incidence, HI prevalence and mortality (proportions)

Age Groups	Total pop.	AOM% incidence			CSOM‰ incidence			HI best ear °/°°°° 25dB > HI ≤ 40dB			40dB > HI ≤ 60dB			60dB > HI ≤ 80dB			80dB > HI			Deaths°/°°°°°°		
		AOM	MIN	MAX	CSOM	MIN	MAX	HI	MIN	MAX	HI	MIN	MAX	HI	MIN	MAX	HI	MIN	MAX	Deaths	MIN	MAX
0-11m.	4673395	30.21	29.94	30.47	3.34	2.06	4.61	2.10	0.00	7.68	0.41	0.00	1.03	0.04	0.00	0.11	0.00	0.00	0.00	19.26	19.26	27.82
1 - 4	17635275	37.99	37.55	38.42	2.23	1.37	3.08	5.23	0.00	10.81	1.02	0.40	1.63	0.11	0.05	0.18	0.01	0.01	0.02	1.70	1.13	2.27
5 - 9	22102033	15.01	14.87	15.17	1.87	1.15	2.58	6.28	0.70	11.86	1.22	0.61	1.84	0.14	0.07	0.21	0.02	0.01	0.03	1.36	0.45	0.45
10 - 14	23699731	10.13	9.95	10.35	2.05	1.26	2.83	6.81	1.22	12.40	1.32	0.71	1.94	0.15	0.08	0.22	0.02	0.01	0.03	0.84	0.42	0.42
15 - 19	23871979	2.48	2.45	2.51	2.56	2.51	2.62	7.33	1.75	12.92	1.42	0.81	2.04	0.16	0.09	0.23	0.02	0.01	0.03	0.42	0.42	0.42
20 - 24	23226793	2.31	2.29	2.34	3.04	2.03	4.05	8.39	2.80	13.97	1.63	1.02	2.25	0.19	0.12	0.25	0.02	0.01	0.03	0.43	0.86	0.86
25 - 34	44886176	1.22	1.21	1.23	2.38	2.03	2.74	9.44	3.85	15.02	1.84	1.22	2.45	0.21	0.14	0.28	0.02	0.02	0.03	0.89	0.67	1.11
35 - 44	49268828	1.23	1.22	1.24	2.75	2.70	2.81	11.55	5.96	17.13	2.25	1.64	2.87	0.26	0.19	0.33	0.03	0.02	0.04	1.22	0.81	1.22
45 - 54	47877462	1.47	1.46	1.49	4.23	3.96	4.49	13.12	7.53	18.70	2.56	1.94	3.18	0.29	0.22	0.37	0.03	0.03	0.04	1.25	0.63	1.04
55 - 64	33890309	1.59	1.58	1.61	4.59	4.84	4.35	15.75	10.16	21.33	3.07	2.46	3.69	0.35	0.28	0.42	0.04	0.03	0.05	0.59	0.59	1.18
65 - 74	20924698	1.77	1.76	1.79	3.38	3.94	2.82	19.95	14.37	25.54	3.90	3.28	4.52	0.45	0.38	0.52	0.05	0.04	0.06	1.91	1.43	2.39
75 - 84	14534112	2.01	2.00	2.04	3.40	3.96	2.83	24.14	18.55	29.72	4.71	4.09	5.32	0.54	0.47	0.61	0.06	0.06	0.07	4.13	3.44	4.82
85 +	5526168	1.95	1.94	1.98	3.44	4.01	2.87	27.23	21.64	32.83	5.28	4.67	5.90	0.60	0.52	0.67	0.07	0.07	0.07	12.67	9.05	16.29
Total	332116959	5.46	5.40	5.53	3.06	2.82	3.30	11.58	6.06	17.16	2.26	1.64	2.87	0.26	0.19	0.33	0.03	0.02	0.04	1.63	1.23	1.90

Table S47. Oceania: 2005 estimates of AOM and CSOM incidence, HI prevalence and mortality (cases)

Age Groups	Total pop.	AOM incidence			CSOM incidence			HI best ear 25dB > HI ≤ 40dB			HI best ear 40dB > HI ≤ 60dB			HI best ear 60dB > HI ≤ 80dB			HI best ear 80dB > HI			Deaths		
		AOM	MIN	MAX	CSOM	MIN	MAX	HI	MIN	MAX	HI	MIN	MAX	HI	MIN	MAX	HI	MIN	MAX	Deaths	MIN	MAX
0-11m.	246899	190747	189052	192442	8878	8751	9004	178	164	192	81	79	83	29	28	30	10	10	10	4	3	4
1 - 4	998043	1147543	1140691	1154394	23945	23433	24457	1794	1738	1850	823	817	829	291	289	293	103	102	104	20	18	23
5 - 9	1184095	493983	485854	502112	23857	23250	24465	2554	2488	2620	1172	1165	1179	415	412	418	147	146	148	11	9	12
10 - 14	1048699	477256	470056	484455	5372	4834	5910	2451	2392	2510	1125	1119	1131	398	396	400	141	140	142	10	8	11
15 - 19	929367	70411	64031	76791	4888	4411	5365	2339	2287	2391	1073	1067	1079	380	378	382	134	133	135	6	5	7
20 - 24	818911	54126	48505	59748	4139	3719	4559	2355	2309	2401	1081	1076	1086	383	381	385	135	134	136	3	3	4
25 - 34	1353931	43924	34630	53219	5922	5227	6616	4381	4305	4457	2010	2002	2018	712	709	715	252	251	253	6	5	6
35 - 44	1066020	34891	27573	42209	4697	4150	5244	4216	4156	4276	1935	1928	1942	685	683	687	242	241	243	7	6	8
45 - 54	695347	27694	22921	32468	1451	1094	1808	3125	3086	3164	1434	1430	1438	508	506	510	180	179	181	4	4	5
55 - 64	391944	17001	14311	19692	670	469	871	2114	2092	2136	970	968	972	343	342	344	122	122	122	4	3	4
65 - 74	196399	9565	8216	10913	391	290	491	1342	1331	1353	616	615	617	218	218	218	77	77	77	6	5	6
75 - 84	64490	3598	3156	4041	128	95	161	533	529	537	245	245	245	87	87	87	31	31	31	8	7	9
85 +	9783	529	461	596	19	14	24	91	90	92	42	42	42	15	15	15	5	5	5	2	2	2
Total	9003928	2571268	2509457	2633080	84357	79737	88975	27473	26967	27979	12607	12553	12661	4464	4444	4484	1579	1571	1587	91	78	101

Table S48. Oceania: 2005 estimates of AOM and CSOM incidence, HI prevalence and mortality (proportions)

Age Groups	Total pop.	AOM% incidence			CSOM‰ incidence			HI best ear °/°°°°												Deaths°/°°°°°°		
								25dB > HI ≤ 40dB			40dB > HI ≤ 60dB			60dB > HI ≤ 80dB			80dB > HI					
		AOM	MIN	MAX	CSOM	MIN	MAX	HI	MIN	MAX	HI	MIN	MAX	HI	MIN	MAX	HI	MIN	MAX	Deaths	MIN	MAX
0-11m.	246899	77.26	76.57	77.94	35.96	35.44	36.47	72.09	66.42	77.76	32.81	32.00	33.62	11.75	11.34	12.15	4.05	4.05	4.05	162.01	121.51	162.01
1 - 4	998043	114.98	114.29	115.67	23.99	23.48	24.50	179.75	174.14	185.36	82.46	81.86	83.06	29.16	28.96	29.36	10.32	10.22	10.42	200.39	180.35	230.45
5 - 9	1184095	41.72	41.03	42.40	20.15	19.64	20.66	215.69	210.12	221.27	98.98	98.39	99.57	35.05	34.79	35.30	12.41	12.33	12.50	92.90	76.01	101.34
10 - 14	1048699	45.51	44.82	46.20	5.12	4.61	5.64	233.72	228.09	239.34	107.28	106.70	107.85	37.95	37.76	38.14	13.45	13.35	13.54	95.36	76.29	104.89
15 - 19	929367	7.58	6.89	8.26	5.26	4.75	5.77	251.68	246.08	257.27	115.45	114.81	116.10	40.89	40.67	41.10	14.42	14.31	14.53	64.56	53.80	75.32
20 - 24	818911	6.61	5.92	7.30	5.05	4.54	5.57	287.58	281.96	293.19	132.00	131.39	132.62	46.77	46.53	47.01	16.49	16.36	16.61	36.63	36.63	48.85
25 - 34	1353931	3.24	2.56	3.93	4.37	3.86	4.89	323.58	317.96	329.19	148.46	147.87	149.05	52.59	52.37	52.81	18.61	18.54	18.69	44.32	36.93	44.32
35 - 44	1066020	3.27	2.59	3.96	4.41	3.89	4.92	395.49	389.86	401.12	181.52	180.86	182.17	64.26	64.07	64.45	22.70	22.61	22.80	65.66	56.28	75.05
45 - 54	695347	3.98	3.30	4.67	2.09	1.57	2.60	449.42	443.81	455.02	206.23	205.65	206.80	73.06	72.77	73.34	25.89	25.74	26.03	57.53	57.53	71.91
55 - 64	391944	4.34	3.65	5.02	1.71	1.20	2.22	539.36	533.75	544.98	247.48	246.97	247.99	87.51	87.26	87.77	31.13	31.13	31.13	102.06	76.54	102.06
65 - 74	196399	4.87	4.18	5.56	1.99	1.48	2.50	683.30	677.70	688.90	313.65	313.14	314.16	111.00	111.00	111.00	39.21	39.21	39.21	305.50	254.58	305.50
75 - 84	64490	5.58	4.89	6.27	1.98	1.47	2.50	826.48	820.28	832.69	379.90	379.90	379.90	134.90	134.90	134.90	48.07	48.07	48.07	1240.50	1085.44	1395.57
85 +	9783	5.41	4.71	6.09	1.94	1.43	2.45	930.19	919.96	940.41	429.32	429.32	429.32	153.33	153.33	153.33	51.11	51.11	51.11	2044.36	2044.36	2044.36
Total	9003928	28.56	27.87	29.24	9.37	8.86	9.88	305.12	299.50	310.74	140.02	139.42	140.62	49.58	49.36	49.80	17.54	17.45	17.63	101.07	86.63	112.17

Table S49. Sub-Saharan Africa, Central: 2005 estimates of AOM and CSOM incidence, HI prevalence and mortality (cases)

Age Groups	Total pop.	AOM incidence			CSOM incidence			HI best ear												Deaths		
								25dB > HI ≤ 40dB			40dB > HI ≤ 60dB			60dB > HI ≤ 80dB			80dB > HI					
		AOM	MIN	MAX	CSOM	MIN	MAX	HI	MIN	MAX	HI	MIN	MAX	HI	MIN	MAX	HI	MIN	MAX	Deaths	MIN	MAX
0-11m.	3565164	3387604	3363129	3412079	79227	77398	81056	2062	1863	2261	549	527	571	101	97	105	22	21	23	46	38	41
1 - 4	12341919	17755936	17671208	17840664	182613	176280	188946	17798	17109	18487	4764	4688	4840	891	877	905	200	197	203	202	190	217
5 - 9	12546854	6472985	6386850	6559120	155385	148947	161823	21605	20904	22306	5814	5737	5891	1104	1089	1119	254	251	257	99	93	107
10 - 14	10699381	6247166	6173714	6320618	47098	41608	52588	19884	19286	20482	5366	5300	5432	1028	1015	1041	241	238	244	84	78	90
15 - 19	9105152	857923	795416	920430	36988	32316	41660	18186	17677	18695	4921	4865	4977	950	939	961	224	221	227	61	61	68
20 - 24	7639611	623525	571079	675971	38351	34431	42271	17412	16985	17839	4719	4672	4766	914	905	923	216	214	218	29	28	31
25 - 34	11287589	448222	370732	525712	42536	36744	48328	28879	28249	29509	7844	7775	7913	1530	1516	1544	368	365	371	51	48	55
35 - 44	7203601	287991	238538	337444	26558	22862	30254	22475	22073	22877	6115	6071	6159	1201	1192	1210	295	293	297	57	53	61
45 - 54	4723467	229887	197460	262314	15707	13283	18131	16696	16432	16960	4566	4537	4595	908	902	914	227	226	228	29	27	31
55 - 64	3019720	160513	139783	181243	8563	7014	10112	12815	12646	12984	3513	3494	3532	700	696	704	175	174	176	27	27	29
65 - 74	1651448	98352	87015	109689	3525	2678	4372	8824	8732	8916	2439	2429	2449	496	494	498	127	126	128	44	41	48
75 - 84	558760	37850	34014	41686	1198	911	1485	3562	3531	3593	1005	1002	1008	214	213	215	60	60	60	68	64	72
85 +	69165	4485	4010	4960	150	115	185	488	484	492	142	142	142	32	32	32	10	10	10	15	14	17
Total	84411831	36612439	36032948	37191930	637899	594587	681211	190686	185971	195401	51757	51239	52275	10069	9967	10171	2419	2396	2442	812	762	867

Table S50. Sub-Saharan Africa, Central: 2005 estimates of AOM and CSOM incidence, HI prevalence and mortality (proportions)

Age Groups	Total pop.	AOM% incidence			CSOM‰ incidence			HI best ear °/°°°°												Deaths°/°°°°°°		
								25dB > HI ≤ 40dB			40dB > HI ≤ 60dB			60dB > HI ≤ 80dB			80dB > HI					
		AOM	MIN	MAX	CSOM	MIN	MAX	HI	MIN	MAX	HI	MIN	MAX	HI	MIN	MAX	HI	MIN	MAX	Deaths	MIN	MAX
0-11m.	3565164	95.02	94.33	95.71	22.22	21.71	22.74	57.84	52.26	63.42	15.40	14.78	16.02	2.83	2.72	2.95	0.62	0.59	0.65	129.03	106.59	115.00
1 - 4	12341919	143.87	143.18	144.55	14.80	14.28	15.31	144.21	138.63	149.79	38.60	37.98	39.22	7.22	7.11	7.33	1.62	1.60	1.64	163.67	153.95	175.82
5 - 9	12546854	51.59	50.90	52.28	12.38	11.87	12.90	172.19	166.61	177.78	46.34	45.72	46.95	8.80	8.68	8.92	2.02	2.00	2.05	78.90	74.12	85.28
10 - 14	10699381	58.39	57.70	59.07	4.40	3.89	4.92	185.84	180.25	191.43	50.15	49.54	50.77	9.61	9.49	9.73	2.25	2.22	2.28	78.51	72.90	84.12
15 - 19	9105152	9.42	8.74	10.11	4.06	3.55	4.58	199.73	194.14	205.32	54.05	53.43	54.66	10.43	10.31	10.55	2.46	2.43	2.49	67.00	67.00	74.68
20 - 24	7639611	8.16	7.48	8.85	5.02	4.51	5.53	227.92	222.33	233.51	61.77	61.15	62.39	11.96	11.85	12.08	2.83	2.80	2.85	37.96	36.65	40.58
25 - 34	11287589	3.97	3.28	4.66	3.77	3.26	4.28	255.85	250.27	261.43	69.49	68.88	70.10	13.55	13.43	13.68	3.26	3.23	3.29	45.18	42.52	48.73
35 - 44	7203601	4.00	3.31	4.68	3.69	3.17	4.20	312.00	306.42	317.58	84.89	84.28	85.50	16.67	16.55	16.80	4.10	4.07	4.12	79.13	73.57	84.68
45 - 54	4723467	4.87	4.18	5.55	3.33	2.81	3.84	353.47	347.88	359.06	96.67	96.05	97.28	19.22	19.10	19.35	4.81	4.78	4.83	61.40	57.16	65.63
55 - 64	3019720	5.32	4.63	6.00	2.84	2.32	3.35	424.38	418.78	429.97	116.34	115.71	116.96	23.18	23.05	23.31	5.80	5.76	5.83	89.41	89.41	96.04
65 - 74	1651448	5.96	5.27	6.64	2.13	1.62	2.65	534.32	528.75	539.89	147.69	147.08	148.29	30.03	29.91	30.16	7.69	7.63	7.75	266.43	248.27	290.65
75 - 84	558760	6.77	6.09	7.46	2.14	1.63	2.66	637.48	631.94	643.03	179.86	179.33	180.40	38.30	38.12	38.48	10.74	10.74	10.74	1216.98	1145.39	1288.57
85 +	69165	6.48	5.80	7.17	2.17	1.66	2.67	705.56	699.78	711.34	205.31	205.31	205.31	46.27	46.27	46.27	14.46	14.46	14.46	2168.73	2024.15	2457.89
Total	84411831	43.37	42.69	44.06	7.56	7.04	8.07	225.90	220.31	231.49	61.31	60.70	61.93	11.93	11.81	12.05	2.87	2.84	2.89	96.20	90.27	102.71

Table S51. Sub-Saharan Africa, East: 2005 estimates of AOM and CSOM incidence, HI prevalence and mortality (cases)

Age Groups	Total pop.	AOM incidence			CSOM incidence			HI best ear												Deaths		
								25dB > HI ≤ 40dB			40dB > HI ≤ 60dB			60dB > HI ≤ 80dB			80dB > HI					
		AOM	MIN	MAX	CSOM	MIN	MAX	HI	MIN	MAX	HI	MIN	MAX	HI	MIN	MAX	HI	MIN	MAX	Deaths	MIN	MAX
0-11m.	11489126	6441660	6362787	6520533	190623	184728	196518	5381	4739	6023	2345	2274	2416	848	822	874	335	325	345	124	100	115
1 - 4	42177845	33860845	33571293	34150397	469341	447699	490983	49753	47397	52109	21533	21274	21792	7730	7637	7823	3033	2996	3070	558	520	595
5 - 9	45740160	13556489	13242481	13870497	429864	406394	453334	65361	62806	67916	28037	27756	28318	9959	9859	10059	3875	3836	3914	279	263	300
10 - 14	39756385	11707070	11434141	11979999	164173	143773	184573	61786	59566	64006	26440	26195	26685	9373	9286	9460	3648	3614	3682	226	211	243
15 - 19	34872616	1840452	1601050	2079854	125929	108035	143823	58284	56336	60232	25123	24908	25338	9006	8929	9083	3544	3514	3574	158	151	171
20 - 24	29511023	1379426	1176832	1582020	147765	132622	162908	56573	54925	58221	24358	24176	24540	8728	8663	8793	3436	3410	3462	85	78	90
25 - 34	43484427	1016655	718133	1315177	154515	132202	176828	94757	92328	97186	40412	40144	40680	14306	14211	14401	5564	5527	5601	153	145	163
35 - 44	27803844	655660	464786	846534	95702	81435	109969	75115	73562	76668	31737	31566	31908	11100	11040	11160	4278	4255	4301	171	157	186
45 - 54	18526673	529501	402315	656687	69259	59753	78765	56952	55917	57987	24024	23910	24138	8388	8348	8428	3231	3216	3246	88	84	90
55 - 64	11666490	360087	279996	440178	37438	31452	43424	43286	42634	43938	18051	17979	18123	6210	6185	6235	2358	2349	2367	88	83	94
65 - 74	6494499	224614	180029	269199	14179	10847	17511	30408	30045	30771	12758	12718	12798	4429	4415	4443	1694	1689	1699	133	122	145
75 - 84	2368565	93452	77192	109712	5188	3973	6403	13313	13181	13445	5637	5622	5652	1982	1977	1987	769	767	771	199	187	211
85 +	315435	12095	9930	14260	697	535	859	2010	1992	2028	856	854	858	302	301	303	119	119	119	47	45	54
Total	314207088	71678006	69520965	73835047	1904673	1743448	2065898	612979	595428	630530	261311	259376	263246	92361	91673	93049	35884	35617	36151	2309	2146	2457

Table S52. Sub-Saharan Africa, East: 2005 estimates of AOM and CSOM incidence, HI prevalence and mortality (proportions)

Age Groups	Total pop.	AOM% incidence			CSOM‰ incidence			HI best ear °/°°°°												Deaths°/°°°°°°		
								25dB > HI ≤ 40dB			40dB > HI ≤ 60dB			60dB > HI ≤ 80dB			80dB > HI					
		AOM	MIN	MAX	CSOM	MIN	MAX	HI	MIN	MAX	HI	MIN	MAX	HI	MIN	MAX	HI	MIN	MAX	Deaths	MIN	MAX
0-11m.	11489126	56.07	55.38	56.75	16.59	16.08	17.10	46.84	41.25	52.42	20.41	19.79	21.03	7.38	7.15	7.61	2.92	2.83	3.00	107.93	87.04	100.09
1 - 4	42177845	80.28	79.59	80.97	11.13	10.61	11.64	117.96	112.37	123.55	51.05	50.44	51.67	18.33	18.11	18.55	7.19	7.10	7.28	132.30	123.29	141.07
5 - 9	45740160	29.64	28.95	30.32	9.40	8.88	9.91	142.90	137.31	148.48	61.30	60.68	61.91	21.77	21.55	21.99	8.47	8.39	8.56	61.00	57.50	65.59
10 - 14	39756385	29.45	28.76	30.13	4.13	3.62	4.64	155.41	149.83	161.00	66.51	65.89	67.12	23.58	23.36	23.79	9.18	9.09	9.26	56.85	53.07	61.12
15 - 19	34872616	5.28	4.59	5.96	3.61	3.10	4.12	167.13	161.55	172.72	72.04	71.43	72.66	25.83	25.60	26.05	10.16	10.08	10.25	45.31	43.30	49.04
20 - 24	29511023	4.67	3.99	5.36	5.01	4.49	5.52	191.70	186.12	197.29	82.54	81.92	83.16	29.58	29.36	29.80	11.64	11.56	11.73	28.80	26.43	30.50
25 - 34	43484427	2.34	1.65	3.02	3.55	3.04	4.07	217.91	212.32	223.50	92.93	92.32	93.55	32.90	32.68	33.12	12.80	12.71	12.88	35.19	33.35	37.48
35 - 44	27803844	2.36	1.67	3.04	3.44	2.93	3.96	270.16	264.57	275.75	114.15	113.53	114.76	39.92	39.71	40.14	15.39	15.30	15.47	61.50	56.47	66.90
45 - 54	18526673	2.86	2.17	3.54	3.74	3.23	4.25	307.41	301.82	312.99	129.67	129.06	130.29	45.28	45.06	45.49	17.44	17.36	17.52	47.50	45.34	48.58
55 - 64	11666490	3.09	2.40	3.77	3.21	2.70	3.72	371.03	365.44	376.62	154.73	154.11	155.34	53.23	53.02	53.44	20.21	20.13	20.29	75.43	71.14	80.57
65 - 74	6494499	3.46	2.77	4.15	2.18	1.67	2.70	468.21	462.62	473.80	196.44	195.83	197.06	68.20	67.98	68.41	26.08	26.01	26.16	204.79	187.85	223.27
75 - 84	2368565	3.95	3.26	4.63	2.19	1.68	2.70	562.07	556.50	567.64	237.99	237.36	238.63	83.68	83.47	83.89	32.47	32.38	32.55	840.17	789.51	890.83
85 +	315435	3.83	3.15	4.52	2.21	1.70	2.72	637.22	631.51	642.92	271.37	270.74	272.01	95.74	95.42	96.06	37.73	37.73	37.73	1490.01	1426.60	1711.92
Total	314207088	22.81	22.13	23.50	6.06	5.55	6.57	195.09	189.50	200.67	83.17	82.55	83.78	29.39	29.18	29.61	11.42	11.34	11.51	73.49	68.30	78.20

Table S53. Sub-Saharan Africa, Southern: 2005 estimates of AOM and CSOM incidence, HI prevalence and mortality (cases)

Age Groups	Total pop.	AOM incidence			CSOM incidence			HI best ear												Deaths		
								25dB > HI ≤ 40dB			40dB > HI ≤ 60dB			60dB > HI ≤ 80dB			80dB > HI					
		AOM	MIN	MAX	CSOM	MIN	MAX	HI	MIN	MAX	HI	MIN	MAX	HI	MIN	MAX	HI	MIN	MAX	Deaths	MIN	MAX
0-11m.	1595755	766472	755517	777427	20976	20157	21795	246	157	335	74	64	84	15	13	17	3	3	3	44	38	57
1 - 4	6259236	4203076	4160106	4246046	54899	51687	58111	2404	2054	2754	732	693	771	152	144	160	32	30	34	20	16	25
5 - 9	7730088	1942395	1889328	1995462	56942	52976	60908	3562	3130	3994	1081	1033	1129	225	215	235	47	45	49	17	17	22
10 - 14	7687307	1809016	1756242	1861790	30294	26349	34239	3838	3409	4267	1163	1116	1210	240	230	250	51	49	53	20	15	25
15 - 19	7404630	326621	275788	377454	24435	20636	28234	3976	3562	4390	1206	1160	1252	248	239	257	52	50	54	5	4	7
20 - 24	7047021	277732	229354	326110	35222	31606	38838	4324	3930	4718	1318	1275	1361	273	264	282	58	56	60	10	10	11
25 - 34	10881383	216102	141401	290803	36873	31289	42457	7533	6925	8141	2311	2244	2378	483	469	497	103	100	106	38	34	51
35 - 44	7617891	152515	100218	204812	24679	20770	28588	6458	6033	6883	1997	1950	2044	420	410	430	90	88	92	53	44	67
45 - 54	5567421	134876	96655	173097	22768	19911	25625	5360	5049	5671	1659	1625	1693	351	344	358	75	73	77	7	6	8
55 - 64	3473830	91396	67548	115244	12258	10475	14041	4018	3824	4212	1245	1224	1266	262	258	266	56	55	57	8	5	8
65 - 74	1918702	56531	43359	69703	4266	3281	5251	2808	2701	2915	865	853	877	183	181	185	39	38	40	1	2	4
75 - 84	711848	23927	19040	28814	1588	1223	1953	1261	1221	1301	385	381	389	80	79	81	17	17	17	2	2	2
85 +	124205	4036	3183	4889	279	215	343	250	243	257	76	75	77	15	15	15	3	3	3	6	5	10
Total	68019317	10004695	9537739	10471651	325479	290575	360383	46038	42238	49838	14112	13693	14531	2947	2861	3033	626	607	645	231	198	297

Table S54. Sub-Saharan Africa, Southern: 2005 estimates of AOM and CSOM incidence, HI prevalence and mortality (proportions)

Age Groups	Total pop.	AOM% incidence			CSOM‰ incidence			HI best ear °/°°°°												Deaths°/°°°°°°		
								25dB > HI ≤ 40dB			40dB > HI ≤ 60dB			60dB > HI ≤ 80dB			80dB > HI					
		AOM	MIN	MAX	CSOM	MIN	MAX	HI	MIN	MAX	HI	MIN	MAX	HI	MIN	MAX	HI	MIN	MAX	Deaths	MIN	MAX
0-11m.	1595755	48.03	47.35	48.72	13.14	12.63	13.66	15.42	9.84	20.99	4.64	4.01	5.26	0.94	0.81	1.07	0.19	0.19	0.19	275.73	238.13	357.20
1 - 4	6259236	67.15	66.46	67.84	8.77	8.26	9.28	38.41	32.82	44.00	11.69	11.07	12.32	2.43	2.30	2.56	0.51	0.48	0.54	31.95	25.56	39.94
5 - 9	7730088	25.13	24.44	25.81	7.37	6.85	7.88	46.08	40.49	51.67	13.98	13.36	14.61	2.91	2.78	3.04	0.61	0.58	0.63	21.99	21.99	28.46
10 - 14	7687307	23.53	22.85	24.22	3.94	3.43	4.45	49.93	44.35	55.51	15.13	14.52	15.74	3.12	2.99	3.25	0.66	0.64	0.69	26.02	19.51	32.52
15 - 19	7404630	4.41	3.72	5.10	3.30	2.79	3.81	53.70	48.11	59.29	16.29	15.67	16.91	3.35	3.23	3.47	0.70	0.68	0.73	6.75	5.40	9.45
20 - 24	7047021	3.94	3.25	4.63	5.00	4.49	5.51	61.36	55.77	66.95	18.70	18.09	19.31	3.87	3.75	4.00	0.82	0.79	0.85	14.19	14.19	15.61
25 - 34	10881383	1.99	1.30	2.67	3.39	2.88	3.90	69.23	63.64	74.82	21.24	20.62	21.85	4.44	4.31	4.57	0.95	0.92	0.97	34.92	31.25	46.87
35 - 44	7617891	2.00	1.32	2.69	3.24	2.73	3.75	84.77	79.20	90.35	26.21	25.60	26.83	5.51	5.38	5.64	1.18	1.16	1.21	69.57	57.76	87.95
45 - 54	5567421	2.42	1.74	3.11	4.09	3.58	4.60	96.27	90.69	101.86	29.80	29.19	30.41	6.30	6.18	6.43	1.35	1.31	1.38	12.57	10.78	14.37
55 - 64	3473830	2.63	1.94	3.32	3.53	3.02	4.04	115.66	110.08	121.25	35.84	35.23	36.44	7.54	7.43	7.66	1.61	1.58	1.64	23.03	14.39	23.03
65 - 74	1918702	2.95	2.26	3.63	2.22	1.71	2.74	146.35	140.77	151.93	45.08	44.46	45.71	9.54	9.43	9.64	2.03	1.98	2.08	5.21	10.42	20.85
75 - 84	711848	3.36	2.67	4.05	2.23	1.72	2.74	177.14	171.53	182.76	54.08	53.52	54.65	11.24	11.10	11.38	2.39	2.39	2.39	28.10	28.10	28.10
85 +	124205	3.25	2.56	3.94	2.25	1.73	2.76	201.28	195.64	206.92	61.19	60.38	61.99	12.08	12.08	12.08	2.42	2.42	2.42	483.07	402.56	805.12
Total	68019317	14.71	14.02	15.40	4.79	4.27	5.30	67.68	62.10	73.27	20.75	20.13	21.36	4.33	4.21	4.46	0.92	0.89	0.95	33.96	29.11	43.66

Table S55. Sub-Saharan Africa, West: 2005 estimates of AOM and CSOM incidence, HI prevalence and mortality (cases)

Age Groups	Total pop.	AOM incidence			CSOM incidence			HI best ear												Deaths		
								25dB > HI ≤ 40dB			40dB > HI ≤ 60dB			60dB > HI ≤ 80dB			80dB > HI					
		AOM	MIN	MAX	CSOM	MIN	MAX	HI	MIN	MAX	HI	MIN	MAX	HI	MIN	MAX	HI	MIN	MAX	Deaths	MIN	MAX
0-11m.	11023920	11199755	11124075	11275435	244643	238986	250300	7456	6840	8072	1937	1869	2005	339	327	351	63	61	65	145	114	133
1 - 4	39812628	61360314	61086999	61633629	587847	567418	608276	66804	64580	69028	17383	17138	17628	3043	3000	3086	572	564	580	664	623	716
5 - 9	43023086	23561235	23265880	23856590	529815	507739	551891	85146	82743	87549	22235	21970	22500	3909	3862	3956	734	725	743	325	304	354
10 - 14	38036395	23576950	23315829	23838071	166917	147400	186434	79907	77783	82031	20957	20723	21191	3704	3663	3745	701	693	709	296	278	319
15 - 19	33024128	3255069	3028357	3481781	133007	116062	149952	73459	71615	75303	19335	19132	19538	3437	3401	3473	650	643	657	210	200	228
20 - 24	27951729	2366952	2175063	2558841	140280	125937	154623	70217	68656	71778	18531	18359	18703	3306	3275	3337	628	622	634	100	92	107
25 - 34	40866939	1688721	1408168	1969274	153280	132310	174250	117052	114770	119334	30757	30506	31008	5460	5415	5505	1041	1032	1050	172	162	186
35 - 44	27164728	1137549	951062	1324036	99672	85733	113611	95393	93876	96910	25022	24855	25189	4429	4399	4459	840	834	846	186	168	197
45 - 54	18591799	949776	822143	1077409	62299	52759	71839	73497	72459	74535	19306	19192	19420	3423	3403	3443	650	646	654	99	94	111
55 - 64	11673493	643799	563660	723938	33571	27581	39561	54432	53780	55084	14349	14277	14421	2556	2543	2569	491	489	493	101	95	106
65 - 74	6605101	407176	361832	452520	14136	10747	17525	39052	38683	39421	10303	10262	10344	1839	1832	1846	353	352	354	157	149	171
75 - 84	2419994	171077	154464	187690	5188	3946	6430	17782	17647	17917	4665	4650	4680	830	827	833	160	159	161	242	228	262
85 +	405288	28618	25836	31400	869	661	1077	3879	3856	3902	988	986	990	171	171	171	30	30	30	63	56	66
Total	300599228	130346991	128283368	132410614	2171524	2017279	2325769	784076	767288	800864	205768	203919	207617	36446	36118	36774	6913	6850	6976	2760	2563	2956

Table S56. Sub-Saharan Africa, West: 2005 estimates of AOM and CSOM incidence, HI prevalence and mortality (proportions)

Age Groups	Total pop.	AOM% incidence			CSOM‰ incidence			HI best ear °/°°°° 25dB > HI ≤ 40dB			40dB > HI ≤ 60dB			60dB > HI ≤ 80dB			80dB > HI			Deaths°/°°°°°°		
		AOM	MIN	MAX	CSOM	MIN	MAX	HI	MIN	MAX	HI	MIN	MAX	HI	MIN	MAX	HI	MIN	MAX	Deaths	MIN	MAX
0-11m.	11023920	101.60	100.91	102.28	22.19	21.68	22.71	67.63	62.05	73.22	17.57	16.95	18.19	3.08	2.97	3.18	0.57	0.55	0.59	131.53	103.41	120.65
1 - 4	39812628	154.12	153.44	154.81	14.77	14.25	15.28	167.80	162.21	173.38	43.66	43.05	44.28	7.64	7.54	7.75	1.44	1.42	1.46	166.78	156.48	179.84
5 - 9	43023086	54.76	54.08	55.45	12.31	11.80	12.83	197.91	192.32	203.49	51.68	51.07	52.30	9.09	8.98	9.20	1.71	1.69	1.73	75.54	70.66	82.28
10 - 14	38036395	61.99	61.30	62.67	4.39	3.88	4.90	210.08	204.50	215.66	55.10	54.48	55.71	9.74	9.63	9.85	1.84	1.82	1.86	77.82	73.09	83.87
15 - 19	33024128	9.86	9.17	10.54	4.03	3.51	4.54	222.44	216.86	228.02	58.55	57.93	59.16	10.41	10.30	10.52	1.97	1.95	1.99	63.59	60.56	69.04
20 - 24	27951729	8.47	7.78	9.15	5.02	4.51	5.53	251.21	245.62	256.79	66.30	65.68	66.91	11.83	11.72	11.94	2.25	2.23	2.27	35.78	32.91	38.28
25 - 34	40866939	4.13	3.45	4.82	3.75	3.24	4.26	286.42	280.84	292.01	75.26	74.65	75.88	13.36	13.25	13.47	2.55	2.53	2.57	42.09	39.64	45.51
35 - 44	27164728	4.19	3.50	4.87	3.67	3.16	4.18	351.16	345.58	356.75	92.11	91.50	92.73	16.30	16.19	16.41	3.09	3.07	3.11	68.47	61.84	72.52
45 - 54	18591799	5.11	4.42	5.80	3.35	2.84	3.86	395.32	389.74	400.90	103.84	103.23	104.45	18.41	18.30	18.52	3.50	3.47	3.52	53.25	50.56	59.70
55 - 64	11673493	5.52	4.83	6.20	2.88	2.36	3.39	466.29	460.70	471.87	122.92	122.30	123.54	21.90	21.78	22.01	4.21	4.19	4.22	86.52	81.38	90.80
65 - 74	6605101	6.16	5.48	6.85	2.14	1.63	2.65	591.24	585.65	596.83	155.99	155.36	156.61	27.84	27.74	27.95	5.34	5.33	5.36	237.70	225.58	258.89
75 - 84	2419994	7.07	6.38	7.76	2.14	1.63	2.66	734.80	729.22	740.37	192.77	192.15	193.39	34.30	34.17	34.42	6.61	6.57	6.65	1000.00	942.15	1082.65
85 +	405288	7.06	6.37	7.75	2.14	1.63	2.66	957.10	951.42	962.77	243.78	243.28	244.27	42.19	42.19	42.19	7.40	7.40	7.40	1554.45	1381.73	1628.47
Total	300599228	43.36	42.68	44.05	7.22	6.71	7.74	260.84	255.25	266.42	68.45	67.84	69.07	12.12	12.02	12.23	2.30	2.28	2.32	91.82	85.26	98.34
